# [^11^C]metomidate PET-CT versus adrenal vein sampling for diagnosing surgically curable primary aldosteronism: a prospective, within-patient trial

**DOI:** 10.1038/s41591-022-02114-5

**Published:** 2023-01-16

**Authors:** Xilin Wu, Russell Senanayake, Emily Goodchild, Waiel A. Bashari, Jackie Salsbury, Claudia P. Cabrera, Giulia Argentesi, Samuel M. O’Toole, Matthew Matson, Brendan Koo, Laila Parvanta, Nick Hilliard, Vasilis Kosmoliaptsis, Alison Marker, Daniel M. Berney, Wilson Tan, Roger Foo, Charles A. Mein, Eva Wozniak, Emmanuel Savage, Anju Sahdev, Nicholas Bird, Kate Laycock, Istvan Boros, Stefan Hader, Victoria Warnes, Daniel Gillett, Anne Dawnay, Elizabeth Adeyeye, Alessandro Prete, Angela E. Taylor, Wiebke Arlt, Anish N. Bhuva, Franklin Aigbirhio, Charlotte Manisty, Alasdair McIntosh, Alexander McConnachie, J. Kennedy Cruickshank, Heok Cheow, Mark Gurnell, William M. Drake, Morris J. Brown

**Affiliations:** 1 Endocrine Hypertension, Department of Clinical Pharmacology, William Harvey Research Institute, Queen Mary University of London, London, United Kingdom; 2 NIHR Barts Cardiovascular Biomedical Research Centre, Barts and The London School of Medicine and Dentistry, Queen Mary University of London, London, United Kingdom; 3 Department of Endocrinology, St Bartholomew’s Hospital, Barts Health NHS Trust, London, United Kingdom; 4 Metabolic Research Laboratories, Wellcome–MRC Institute of Metabolic Science, University of Cambridge, Cambridge, United Kingdom; 5 NIHR Cambridge Biomedical Research Centre, Addenbrooke’s Hospital, Cambridge University Hospitals NHS Foundation Trust, Cambridge, United Kingdom; 6 Department of Diabetes and Endocrinology, Addenbrooke’s Hospital, Cambridge University Hospitals NHS Foundation Trust, Cambridge, United Kingdom; 7 Centre for Translational Bioinformatics, William Harvey Research Institute, Queen Mary University of London, London, United Kingdom; 8 Department of Endocrinology, Royal Hallamshire Hospital, Sheffield, United Kingdom; 9 Department of Radiology, St Bartholomew’s Hospital, Barts Health NHS Trust, London, United Kingdom; 10 Department of Radiology, Addenbrooke’s Hospital, Cambridge University Hospitals NHS Foundation Trust, Cambridge, United Kingdom; 11 Department of Surgery, Addenbrooke’s Hospital, Cambridge University Hospitals NHS Foundation Trust, Cambridge, United Kingdom; 12 Department of Histopathology, Addenbrooke’s Hospital, Cambridge University Hospitals NHS Foundation Trust, Cambridge, United Kingdom; 13 Department of Histopathology, St Bartholomew’s Hospital, Barts Health NHS Trust, London, United Kingdom; 14 Cardiovascular Research Institute, National University of Singapore, Singapore, Singapore; 15 Barts and the London Genome Centre, School of Medicine and Dentistry, Blizard Institute, London, United Kingdom; 16 Wolfson Brain Imaging Centre, University of Cambridge, Cambridge, United Kingdom; 17 Department of Nuclear Medicine, Addenbrooke’s Hospital, Cambridge University Hospitals NHS Foundation Trust, Cambridge, United Kingdom; 18 Department of Clinical Biochemistry, St Bartholomew’s Hospital, Barts Health NHS Trust, London, United Kingdom; 19 Department of Cardiovascular Medicine/Diabetes, Guy’s and St Thomas’ NHS Foundation Trust, London, United Kingdom; 20 Institute of Metabolism and Systems Research, University of Birmingham, Birmingham, United Kingdom; 21 NIHR Birmingham Biomedical Research Centre, University Hospitals Birmingham NHS Foundation Trust and University of Birmingham, Birmingham, UK; 22 Department of Cardiology, St Bartholomew’s Hospital, Barts Health NHS Trust, London, United Kingdom; 23 Robertson Centre for Biostatistics, University of Glasgow, Glasgow, United Kingdom; 24 School of Life Course/Nutritional Sciences, King’s College London, London, United Kingdom

**Keywords:** Adrenal gland diseases, Hypertension

## Abstract

Primary aldosteronism (PA) due to a unilateral aldosterone-producing adenoma is a common cause of hypertension. This can be cured, or greatly improved, by adrenal surgery. However, the invasive nature of the standard pre-surgical investigation contributes to fewer than 1% of patients with PA being offered the chance of a cure. The primary objective of our prospective study of 143 patients with PA (NCT02945904) was to compare the accuracy of a non-invasive test, [^11^C]metomidate positron emission tomography computed tomography (MTO) scanning, with adrenal vein sampling (AVS) in predicting the biochemical remission of PA and the resolution of hypertension after surgery. A total of 128 patients reached 6- to 9-month follow-up, with 78 (61%) treated surgically and 50 (39%) managed medically. Of the 78 patients receiving surgery, 77 achieved one or more PA surgical outcome criterion for success. The accuracies of MTO at predicting biochemical and clinical success following adrenalectomy were, respectively, 72.7 and 65.4%. For AVS, the accuracies were 63.6 and 61.5%. MTO was not significantly superior, but the differences of 9.1% (95% confidence interval = −6.5 to 24.1%) and 3.8% (95% confidence interval = −11.9 to 9.4) lay within the pre-specified −17% margin for non-inferiority (*P* = 0.00055 and *P* = 0.0077, respectively). Of 24 serious adverse events, none was considered related to either investigation and 22 were fully resolved. MTO enables non-invasive diagnosis of unilateral PA.

## Abstract (web summary)

Comparison of non-invasive [^11^C]metomidate PET-CT with adrenal vein sampling for predicting biochemical remission of primary aldosteronism showed non-superiority, suggesting that the non-invasive method is suitable for the diagnosis of unilateral primary aldosteronism.

## Main

Hypertension is a major cause of deaths from stroke, coronary heart disease, heart failure and renal failure. The most common single cause of hypertension is primary aldosteronism (PA), accounting for 5–14% of all cases and 20–25% of treatment-resistant hypertension^1–4^. The cardiometabolic consequences of hypertension are approximately twice as likely in individuals with PA compared with age- and sex-matched individuals with essential hypertension^5–8^. Traditionally, patients with PA are divided equally into those in whom aldosterone excess is due to a unilateral aldosterone-producing adrenal adenoma (APA), in whom surgical cure is possible, and those with bilateral production (often termed idiopathic hyperaldosteronism (IHA)), who require long-term medical therapy. Although recent studies point to a continuum of disease between unilateral and bilateral subtypes, their distinction underpins the binary clinical decision of whether to remove one adrenal gland, and drives the investigations required to make this decision^9,10^. Current consensus is that patients with unilateral APAs should be offered laparoscopic surgical removal of that adrenal gland and those with IHA should be treated with aldosterone antagonist drugs^11^.

Despite the prevalence and penalty of PA, currently, fewer than 1% of patients are identified and fully investigated^12^. Many reasons exist for this shortfall in clinical care, including: low levels of clinical suspicion; time-consuming and imperfect confirmatory biochemical tests that require discontinuation of many commonly prescribed antihypertensive drugs; difficulties with patient selection for surgical intervention; and uncertainty about outcomes in patients hoping for surgical cure. The paradoxical consequence is patient and physician reluctance to embark on a diagnostic and therapeutic process with a high likelihood of major clinical benefit, and that often sub-optimal medical treatment remains the default for the overwhelming majority of patients^7,11^.

Molecular imaging has the potential to increase the number of patients with PA who can be fully investigated. The current criterion standard used to distinguish patients with APA from those with IHA is adrenal vein sampling (AVS)—an invasive, technically demanding procedure with restricted availability. Metomidate—a methyl analog of the anesthetic agent etomidate—is a potent inhibitor of CYP11B1 (11β-hydroxylase) and CYP11B2 (aldosterone synthase), the final two enzymes involved in cortisol and aldosterone synthesis, respectively. Metomidate can be ^11^CH_3_ labeled and used as a positron emission tomography (PET) radiotracer in combination with high-resolution computed tomography (CT) to detect adrenocortical tumors expressing these enzymes. A previous proof-of-concept study indicated that pre-treatment with dexamethasone for 3 d before [^11^C]metomidate PET-CT (MTO) scanning suppresses adrenal CYP11B1 (but not CYP11B2) protein expression, thus achieving in vivo selectivity^13^. This selectivity facilitates the detection of focal adrenal lesions with high [^11^C]metomidate uptake due to CYP11B2 expression and separates them from normal adrenal tissue. International guidelines for the management of PA acknowledge the potential for molecular imaging in the subtyping of PA^11^, but prospective outcomes-based data are needed to influence clinical practice.

**Fig. 1:**
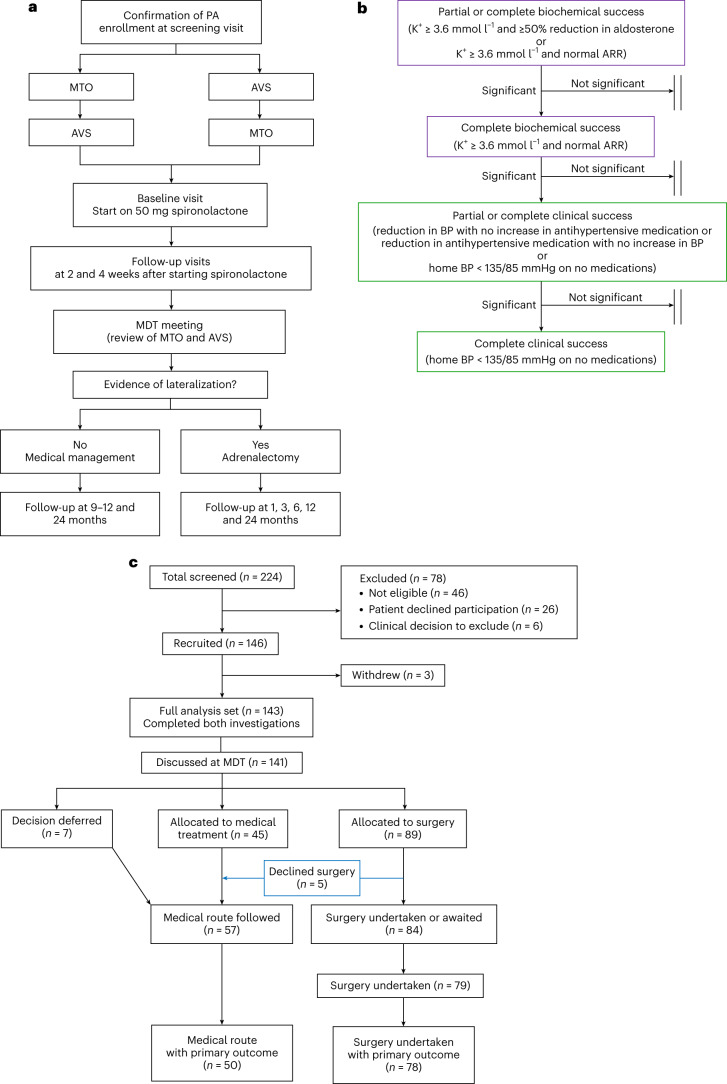
Study outline, hierarchical co-primary endpoints and CONSORT diagram. **a**, Study outline. Patients with confirmed PA according to the Endocrine Society consensus guidelines underwent both AVS and MTO in random order. All patients were subsequently reviewed at a baseline visit and started on 50 mg spironolactone, which was up-titrated to 100 mg after 2 weeks. The blood pressure response to spironolactone was recorded at 2 and 4 weeks from the initiation of spironolactone. Eplerenone was used in those previously intolerant of spironolactone. Concurrently, the results of both investigations were reviewed at multidisciplinary team (MDT) meetings where MTO was always reviewed first, followed by AVS. A score of high, intermediate or low probability of unilateral PA was assigned to each investigation. Recommendations for unilateral adrenalectomy were made if either investigation indicated a high probability of unilateral PA or, in a small number of patients, where both investigations indicated intermediate probability and there was a clinical indication for surgery (for example, because of drug intolerance or uncontrolled blood pressure). Note that the blood pressure response to spironolactone was not used by the MDT in the decision-making process for recommending adrenalectomy. **b**, Hierarchical co-primary endpoints. The table shows definitions of partial and complete biochemical or clinical success, as defined by the PASO consensus (Supplementary Table 1)^14^, and the hierarchical order in which each definition of cure post-adrenalectomy was assessed. On an individual basis, a successful outcome was not dependent on success at the previous limb of the hierarchy. BP, blood pressure; K^+^, potassium. **c**, CONSORT diagram showing the disposition of study patients. Adrenalectomy was undertaken in 79 patients, of whom 78 had evaluable primary outcome data (assessed at 6 months post-surgery or after 9–12 months of medical therapy with spironolactone). Note that both biochemical and clinical primary outcome data were available in 77/78 patients. Only clinical primary outcome data were available for the remaining participant.

The primary aim of this study, which we have termed MATCH (Is Metomidate superior to AVS in predicting ouTComes from adrenalectomy in primary Hyperaldosteronism), was to compare the accuracy of AVS and MTO in predicting the outcome from adrenalectomy in patients with PA (Fig. 1a). The diagnosis of PA, the protocol for and interpretation of AVS, and the biochemical and clinical outcomes after surgery were all assessed and conducted according to international consensus guidelines and/or statements (Fig. 1b and Supplementary Table 1)^11,14,15^. MTO scanning was performed as previously described^13^, and interpreted and graded before, and in ignorance of, the information provided by AVS. The design of the study also permitted the inclusion of a number of mechanistic secondary endpoints that have the potential to refine patient selection for surgery. These mechanistic endpoints included: a trial of aldosterone antagonist therapy (spironolactone or eplerenone) in predicting surgical cure; the influence of ethnicity and tumor genotype on surgical outcome; and the consequences of surgical and medical intervention on indices of cardiac injury and function, as determined by changes in amino (N)-terminal prohormone of brain natriuretic peptide (NT-pro BNP) levels and cardiac chamber dimensions on MRI.

## Results

A total of 146 patients were recruited from endocrinology or hypertension clinics in three centers in the United Kingdom between 2 December 2016 and 11 September 2020. The final study visit was completed on 19 February 2021 and the data were censored on 26 February 2021 when the required number of patients (>75) had primary outcome measurements following surgery. Further details on the study design and inclusion and exclusion criteria are provided in the Methods. Patient flow is shown in the CONSORT diagram (Fig. 1c). Because of the interruptions to elective surgery during 2020 as a consequence of the COVID-19 pandemic, primary outcomes were outstanding in six patients allocated to surgery in whom surgery and 6-month follow-up had not been completed by February 2021. The results are therefore presented for the 128 patients in the analysis set who had either completed primary follow-up from surgery (*n* = 78) or enrolled for and been allocated to medical treatment in the same time period as the 78 patients who received surgery. The baseline data are shown in Table 1. Of the 128 participants, 87 (68%) were male and 41 (32%) were female. The median age was 52 years (interquartile range = 43–60 years). Participants self-identified as one of the following ethnicities: 58% White, 30% Black, 11% Asian and 1% other. The proportions of individuals in the 128-patient analysis set (Table 1) were similar to those of the entire cohort, which includes those without primary outcomes (Extended Data Table 1). In total, 57 of the 128 patients (45%) met the Endocrine Society trinity of plasma aldosterone >550 pmol l^−1^, suppressed renin and spontaneous hypokalemia^11^ at recruitment (information on spontaneous hypokalemia was ascertained by measurement at referral or by searching previous records).

**Table 1:** Demographics and baseline characteristics of participants in the analysis set

	All participants^a^ (*n* = 128)	Surgery (*n* = 78)	Medical (*n* = 50)	*P* value for differences between treatment groups
Sex				0.703
Male	87 (68.0%)	54 (69.2%)	33 (66.0%)	
Female	41 (32.0%)	24 (30.8%)	17 (34.0%)	
Median age (years)	52 (42.5–60.0)	52 (43–60)	52.5 (41–61)	0.874
Median BMI (kg m^−2^)	29.7 (26.7–34.0)	30.5 (26.3–34.8)	29.1 (26.9–33.2)	0.336
Ethnicity				0.007
White	75 (58.6%)	53 (67.9%)	22 (44%)	
Black^b^	38 (29.7%)	15 (19.2%)	23 (46%)	
Asian	14 (10.9%)	9 (11.5%)	5 (10%)	
Other	1 (0.8%)	1 (1.3%)	0 (0%)	
Clinic BP at screening				
SBP median (mmHg)	148 (138–166)	149 (138–166)	145 (138–166)	0.705
DBP median (mmHg)	91 (82–103)	91 (81–102)	89 (84–104)	0.667
Median NDC	2 (2–3)	2 (2–3)	2 (2–3)	0.929
Median DDD	3.5 (2.1–6.0)	4.0 (2.3–6.5)	3.1 (2.0–6.0)	0.199

^a^The demographics and baseline characteristics of patients in the analysis set (that is, those who had completed their allocated treatment and follow-up (6 months post-adrenalectomy or after 9–12 months of medical therapy) at the time the study were censored). Data for patients managed with surgery or medical therapy are also shown.

^b^Black patients comprised 40% of 108 patients recruited in London hospitals, where 14% of the local population is Black.

Numbers in brackets indicate percentages, whereas ranges in brackets indicate first and third quartiles. For categorical data, the Fisher’s exact test was used. For continuous data, the Mann–Whitney–Wilcoxon test was used.

BMI, body mass index; BP, blood pressure; DDD, defined daily dose of antihypertensive medication; DBP, diastolic blood pressure; NDC, number of different classes of antihypertensive medication.

### Scoring of investigations

**Fig. 2:**
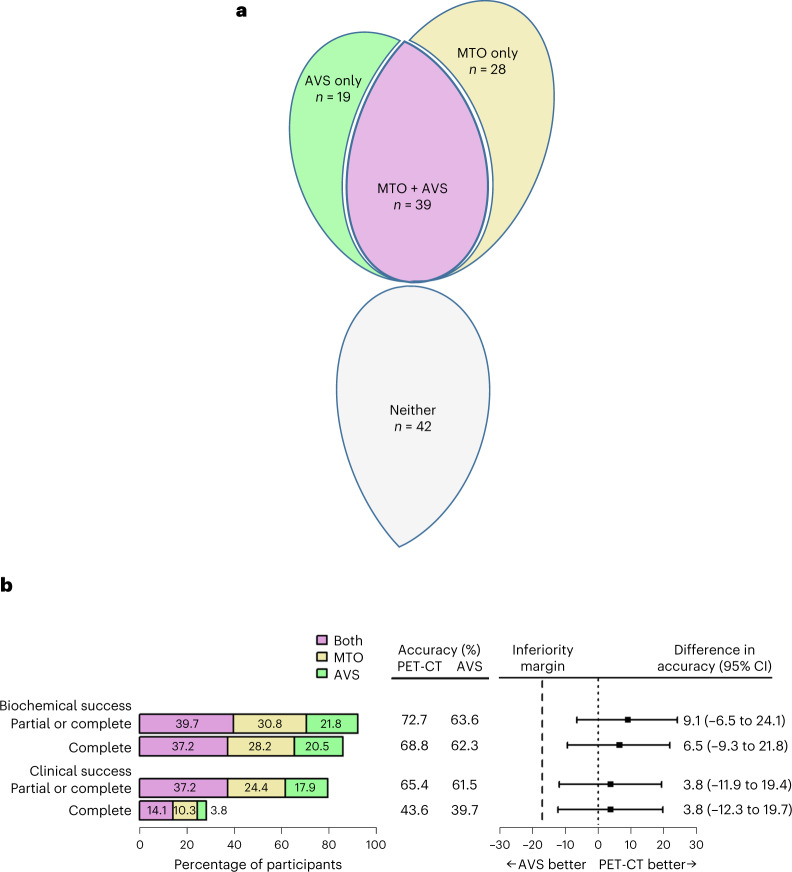
Primary outcomes. **a**, Allocations to adrenalectomy and concordance of MTO and AVS. The number of patients scored as having a high probability of unilateral PA is shown for each independently scored investigation. The Venn diagram shows whether a high-probability score was achieved by one or both tests and includes the concordance between the investigations. 'Neither' indicates the number of patients who did not score as having a high probability of unilateral PA by either investigation and who were therefore recommended for medical therapy (total *n* = 128). **b**, Comparison of the accuracy of MTO and AVS in predicting a successful outcome from adrenalectomy. The four hierarchically analyzed measures of success (as outlined by the PASO consensus) are shown. The horizontal bars in the left panel are color coded as in **a**, reporting the proportion of high-probability scores for MTO or AVS, together or alone, that correctly predicted success. The middle panel shows the accuracy of each investigation, expressed as a percentage. The accuracy for each investigation was calculated as the number of patients who were scored as having a high probability of unilateral PA by that investigation (purple and gold bars for MTO and purple and green bars for AVS) and who achieved success, minus the number of patients in whom the investigation failed to predict a cure. The percentages exclude from the denominator the three patients who underwent adrenalectomy based on two medium-probability scores, on the basis of clinical grounds. The right panel shows the difference between the accuracies of MTO and AVS and the 95% CI around the difference, plotted for each outcome. All four sets of CI intervals cross zero, indicating that neither investigation was superior to the other. None of the lower bounds of the CI intervals cross the pre-specified margin of −17%, indicating that MTO is not inferior to AVS. The center of each error bar is the estimated difference in accuracy (%). *P* values for non-inferiority, from top to bottom, were: *P* = 0.00055, *P* = 0.0024, *P* = 0.0077 and *P* = 0.0091. The BinomDiffCI() function from R was used to calculate the 95% CI^61^ and to estimate the *P* values using a two-sided test (*n* = 78 for clinical success and *n* = 77 for biochemical success as post-operative biochemical data were not available for one patient; complete clinical success was achieved in this patient).

**Extended Data Fig. 1:**
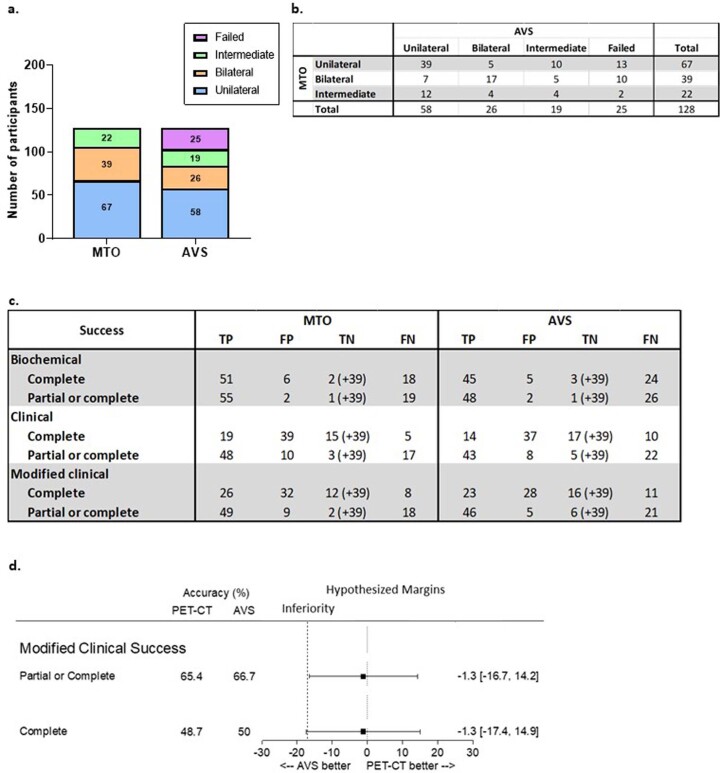
Results from MTO and AVS. **a**. Number of patients in whom MTO or AVS identified unilateral or bilateral primary aldosteronism (PA). ‘Intermediate’ suggests some asymmetry in the adrenals but not meeting criteria for unilateral disease. ‘Failed’ indicates unsuccessful cannulation of adrenal vein(s) and therefore inability to interpret AVS result. n = 128, all patients who reached the primary endpoint time-point (6 or 9-12 months post surgery or medical therapy respectively). **b**. Cross tabulation table showing results from MTO and AVS, indicating relative contribution of each investigation. n = 128. **c**. Accuracy of each investigation in predicting biochemical and clinical outcomes post surgery. The table assumes the 39 patients found to have bilateral disease on both investigations and did not go for surgery, indicated by brackets, were true negatives. This assumption (both investigations were accurate) allows calculation of the false positive rates but does not differentiate between the relative performance of each investigation, which is determined by cases where the investigation outcomes disagreed. *n = 116 for biochemical success, n = 117 for clinical success, based on a total of 78 patients who went underwent adrenalectomy and 39 who were thought to have bilateral disease based on both investigations. TP, true positive; FP, false positive; TN, true negative; FN, false negative. **d**. Modified clinical success outcomes. Forest plot of modified clinical success outcomes, determined by change in number of classes of antihypertensive medications (for definitions please refer to Supplementary Table 3). Column 2 and 3 show percentage of success accurately predicted by MTO and AVS respectively. Right hand column show percentage difference. The centre for the error bars is the Estimated Difference in Accuracy in (%). It, along with the 95% CI (shown in square brackets), is given in the figure’s last 3 columns. Non-inferiority p values: partial or complete modified clinical success p = 0.045; complete modified clinical success p = 0.057. The BinomDiffCI() function from R was used to calculate the 95% CI^61^ and to estimate the p-values using a 2-sided test. n = 78.

MTO and AVS graded 67/128 (52%) and 58/128 (45%) patients, respectively, as having a high probability of unilateral PA (indicating surgery). MTO and AVS were concordant in the diagnosis of unilateral disease in 39/128 (30%) patients, whereas 47/128 patients (37%) were diagnosed by one of MTO or AVS alone (Fig. 2a and Extended Data Fig. 1a,b). In sum, 86/128 patients (67%) were scored as having a high probability of unilateral PA. It is apparent that this higher than usual proportion of patients was mainly due to the number in whom MTO was positive, over and above those diagnosed by AVS^11^. However, two of the 86 patients lateralized to opposite sides on MTO and AVS. Referrals for adrenalectomy were, per protocol, deferred in these two patients, and in four others with medical contraindications.

Five patients with a high-probability score did not proceed to surgery because of personal or practical objections. Conversely, three patients proceeded to surgery on a clinical indication—usually intractable hypertension and intermediate scores on both MTO and AVS. These were the only patients in whom it was the combination of both investigations that led to a surgical referral. In subsequent analyses of outcome, both MTO and AVS were scored as negative in these three patients and, being concordant, neither investigation was favored in the analysis.

### Primary outcomes

In total, 78 patients had evaluable data 6 months after surgery, of whom 77 achieved at least one of the hierarchical primary outcomes. Of the 78 patients with evaluable data, 69 (88%) achieved complete biochemical success and 24 (31%) achieved complete clinical success (Fig. 2b). All four PA surgical outcome (PASO) measures of success were as likely to be achieved whether patients were diagnosed by one or both of the investigations. This was apparent from comparing the proportion of patients predicted to have unilateral disease with the proportion of patients achieving success. For the primary analysis of the accuracy of each investigation, one point was assigned to one or both investigations if they correctly predicted success and to the other or neither investigation if they wrongly predicted success (Extended Data Fig. 1c). The Forest plot for the four sequentially analyzed primary outcomes (Fig. 2b) shows the differences in accuracy to favor MTO over AVS in correctly predicting biochemical and clinical success. The 95% confidence intervals (CIs) for each difference crossed zero, excluding significant superiority for MTO. However, the lower bounds did not cross −17%. This was the pre-specified margin for recognizing non-inferiority, indicating that MTO is at least as accurate as AVS in predicting each of the biochemical and clinical outcomes from adrenalectomy.

Accuracy was influenced mainly by the proportion of true to false positive diagnoses, since adrenal glands were not removed from the patients in the medical group to validate negative diagnoses. Indeed, for estimation of false positive rates, we assumed that all patients assigned to medical therapy were true negatives (Extended Data Fig. 1c). Using the first of the four hierarchical outcomes (namely, complete or partial biochemical success), the true positive rate (sensitivity) was 55/74 (74.3% (95% CI = 62.8 to 83.8%)) for MTO and 48/74 (64.9%; 95% CI = 52.9 to 75.6%) for AVS. The false positive rates (1 − specificity) were 2/42 (4.8% (95% CI = 0.6 to 16.2%)) for both MTO and AVS.

An additional analysis found a small impact of AVS failures on the relative accuracy of MTO and AVS in predicting partial or complete biochemical success, but not on the other three PASO outcomes (Supplementary Table 2). The modified clinical outcomes (Supplementary Table 3) are shown in Extended Data Fig. 1d.

### Secondary outcomes

The quantitative data at baseline and primary follow-up used to calculate the binary success outcomes in Fig. 2 are shown in Extended Data Table 2, together with the comparable data for patients in the group that was treated medically. The non-randomized comparison of outcomes after surgical or medical treatment is an analysis of how these two groups responded to the removal of aldosterone excess or mineralocorticoid receptor blockade, respectively.

**Fig. 3:**
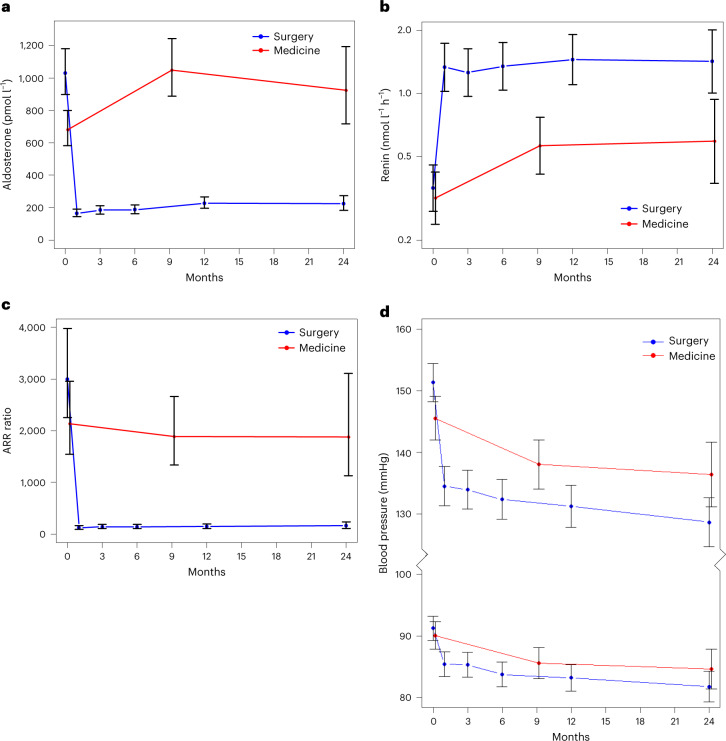
Secondary (pre- and post-intervention) outcomes. **a**, Change in plasma aldosterone levels. For the surgical group, *n* = 80 at 0 months, *n* = 66 at 6 months, *n* = 57 at 12 months and *n* = 28 at 24 months. For the medical group, *n* = 59 at 0 months, *n* = 50 at 9 months and *n* = 18 at 24 months. **b**, Change in plasma renin activity. For the surgical group, *n* = 74 at 0 months, *n* = 69 at 6 months, *n* = 57 at 12 months and *n* = 29 at 24 months. For the medical group, *n* = 60 at 0 months, *n* = 48 at 9 months and *n* = 17 at 24 months. **c**, Change in the ARR. For the surgical group, *n* = 74 at 0 months, *n* = 66 at 6 months, *n* = 55 at 12 months and *n* = 28 at 24 months. For the medical group, *n* = 58 at 0 months, *n* = 48 at 9 months and *n* = 16 at 24 months. Reduction in aldosterone, de-suppression of renin and consequent reduction in ARR were seen post-surgery. ARR remained unchanged with medical therapy. **d**, Reduction in systolic and diastolic home blood pressure post-treatment. All effects seen were sustained for at least 2 years. For the surgical group, *n* = 76 at 0 months, *n* = 67 at 6 months, *n* = 56 at 12 months and *n* = 33 at 24 months. For the medical group, *n* = 59 at 0 months, *n* = 41 at 9 months and *n* = 19 at 24 months. The data represent least-squares means from mixed-effects models, adjusted for baseline covariates. Error bars show 95% CIs. 0 months indicates the baseline visit. Reference ranges were 150–550 pmol l^−1^ for aldosterone, 0.5–3.0 nmol l^−1^ h^−1^ for renin activity and <1,000 pmol l^−1^ (nmol l^−1^ h^−1^) for ARR (activity).

**Extended Data Fig. 2:**
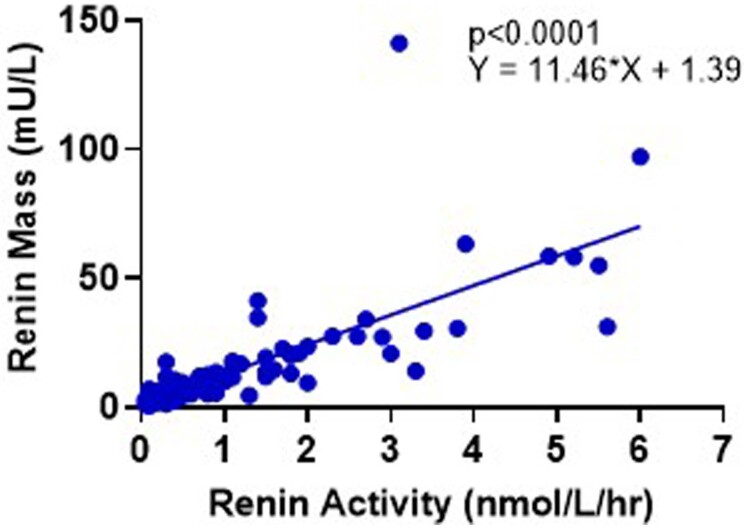
Conversion between renin activity and mass. Different assays were used for the measurement of renin at the three sites. CUH and GSTT measured renin mass (mU/L), while SBH measured renin activity (nmol/L/hr). A renin mass level (pg/ml) was also sent off at selected (baseline and 6-9 month) visits at the SBH site to allow within-patient comparisons between renin mass and activity. A conversion of 1.67 was used to convert between renin mass measured in pg/ml to mU/L. The scatter plot above shows correlation between renin activity and mass within the SBH cohort. Pearson’s correlation *r* = 0.7851 (95% CI 0.7107 – 0.8422), p < 0.0001 (two-tailed test). For the study a conversion factor of 10.989 was used for conversion from renin activity (nmol/L/hr) to mass (mU/L), taken from the ARR thresholds for diagnosing PA: ARRactivity 1000, ARRmass 91 (1000/91 = 10.989). n = 136. CUH, Cambridge University Hospital; SBH, St Bartholomew’s Hospital; GSTT, Guy’s and St Thomas’ Hospital; ARR, aldosterone renin ratio; PA primary aldosteronism.

**Extended Data Fig. 3:**
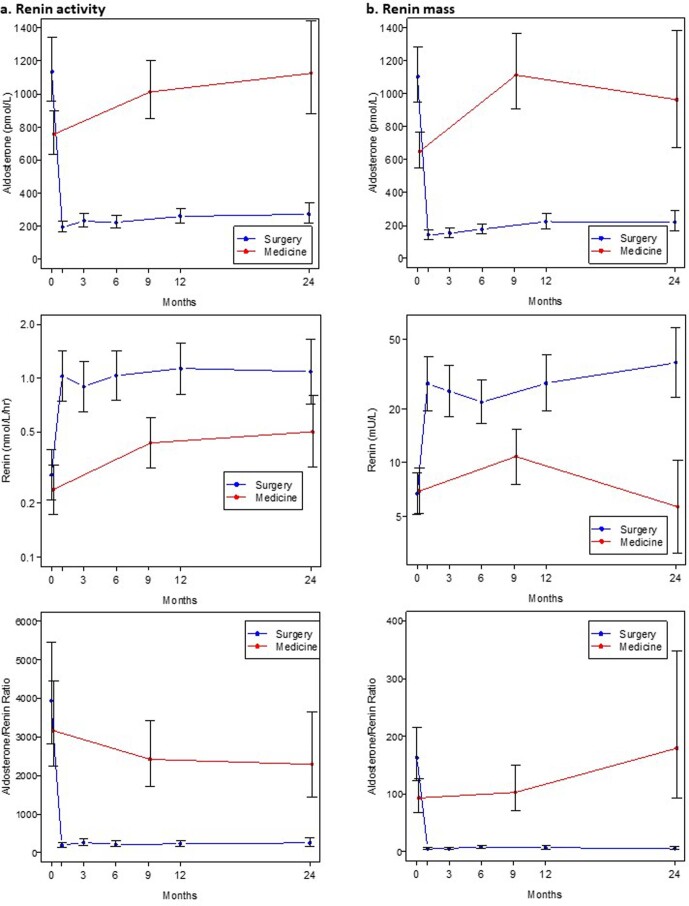
Pre- and post-intervention outcomes. Change in aldosterone, renin and ARR observed at baseline (time 0) and after surgery and medical therapy, divided into cohorts where **a. Renin activity** (SBH) and **b. Renin mass** was measured (GSTT and CUH). A similar trend is observed in both cohorts. Data are least squares means from mixed effects models, adjusted for baseline covariates. Error bars show 95% CI. 0 months indicate baseline visit. For aldosterone levels with renin mass: Surgical group: n = 64 at 0 months, n = 51 at 6 months, n = 27 at 12 months, n = 15 at 24 months. Medical group: n = 50 at 0 months, n = 32 at 9 months, n = 9 at 24 months. For renin as renin mass: Surgical group: n = 64 at 0 months, n = 53 at 6 months, n = 28 at 12 months, n = 16 at 24 months. Medical group: n = 51 at 0 months, n = 32 at 9 months, n = 10 at 24 months. For ARR calculated using renin mass: Surgical group: n = 64 at 0 months, n = 51 at 6 months, n = 27 at 12 months, n = 15 at 24 months. Medical group: n = 50 at 0 months, n = 32 at 9 months, n = 9 at 24 months. For aldosterone levels with renin activity: Surgical group: n = 41 at 0 months, n = 40 at 6 months, n = 35 at 12 months, n = 18 at 24 months. Medical group: n = 40 at 0 months, n = 40 at 9 months, n = 16 at 24 months. For renin as renin activity: Surgical group: n = 41 at 0 months, n = 41 at 6 months, n = 36 at 12 months, n = 18 at 24 months. Medical group: n = 42 at 0 months, n = 40 at 9 months, n = 16 at 24 months. For ARR calculated using renin activity: Surgical group: n = 41 at 0 months, n = 40 at 6 months, n = 35 at 12 months, n = 18 at 24 months. Medical group: n = 40 at 0 months, n = 40 at 9 months, n = 16 at 24 months. SBH, St Bartholomew’s Hospital; GSTT, Guy’s and St Thomas’ Hospital; CUH, Cambridge University Hospital.

Biochemically, there were large and sustained changes in aldosterone, renin and the aldosterone-to-renin ratio (ARR) (Fig. 3a–c). Plasma aldosterone was reduced in all patients after surgery, by a mean of 83%, but increased in the medically treated group (Extended Data Table 2). While renin was de-suppressed in both groups, the increase was greater in the patients receiving surgery than in the patients treated with medicine. The conversion between renin activity and renin mass is shown in Extended Data Fig. 2, while Extended Data Fig. 3 shows the change in biochemistry grouped by renin mass and activity separately.

Blood pressures in the surgical group were higher at baseline and lower at follow-up than in the medical group (Extended Data Table 2 and Fig. 3d). These analyses did not include the initial blood pressure responses to 100 mg spironolactone daily, which are reported below.

### Safety

There were a total of 28 serious adverse events (SAEs). None were considered related to either investigation and 26 were fully resolved (Table 2).

**Table 2:** SAEs

SAE number	Diagnosis	SAE related to study	Outcome
1	Hypokalemia following AVS	No	Resolved
2	Hospital admission for headache	No	Resolved
3	Hospital admission for influenza	No	Resolved
4	Pregnancy	No	Resolved
5	Inpatient admission for headache with dizziness (labrynthitis)	No	Resolved
6	Pulmonary embolism	No	Resolved
7	Inpatient admission for hyperkalemia (at 1 month post-adrenalectomy)	No	Resolved
8	Inpatient admission for hyperkalemia (medically treated)	No	Resolved
9	Inpatient admission for abdominal pain	No	Resolved
10	Bile leak secondary to gallbladder perforation, with subsequent cholecystecyomy	No	Resolved
11	Inpatient admission for hyperkalemia	No	Resolved
12	Post-operative hypoadrenalism	No	Resolved
13	Breast cancer	No	Recovering
14	Inpatient admission for trismus and oropharyngeal infection	No	Resolved
15	Inpatient admission of COVID-19 pneumonitis	No	Resolved
16	Inpatient admission for supraventricular tachycardia	No	Resolved
17	Inpatient admission for severe hypertension	No	Resolved
18	Inpatient admission for hyperkalemia and worsening renal failure	No	Resolved
19	Inpatient admission for seizure	No	Resolved
20	COVID-19 pneumonia	No	Resolved
21	Inpatient admission for worsening renal failure	No	Resolved
22	Squamous cell carcinoma of the tongue	No	Resolved
23	Incidental finding of high troponin from study bloods	No	Resolved
24	Deterioration in renal function	No	Ongoing
25	Post-operative hospital-acquired pneumonia	No	Resolved
26	Angioedema secondary to ramipril	No	Resolved
27	Deep-vein thrombosis	No	Resolved
28	Fractured humerus	No	Resolved

### Effect of spironolactone on MTO lateralization

AVS and MTO were performed with patients taking medications that did not interfere with the renin–angiotensin system. We also wanted to establish whether leaving patients on spironolactone at the time of their MTO would prevent the detection of unilateral disease. Six patients underwent MTO scanning on two separate occasions: once having discontinued spironolactone (as part of the standard protocol) and on a separate occasion while taking at least 50 mg spironolactone. The dose of spironolactone was not intended to achieve complete aldosterone blockade, but to reflect typical doses used in clinical practice. In five out of six patients, spironolactone did not change the scoring of MTO. In the sixth case, the MTO scan without spironolactone (the core protocol scan and the one used for analysis) was graded as a low probability of unilateral disease, whereas the scan performed while taking spironolactone indicated a high probability. AVS was concordant with the MTO results on spironolactone, as it too indicated unilateral PA. Post-adrenalectomy, this patient achieved complete biochemical and partial clinical success. Overall, there was no evidence that MTO performed while on spironolactone reduced the probability of detecting unilateral PA.

### NT-pro BNP and CMR sub-study

**Extended Data Fig. 4:**
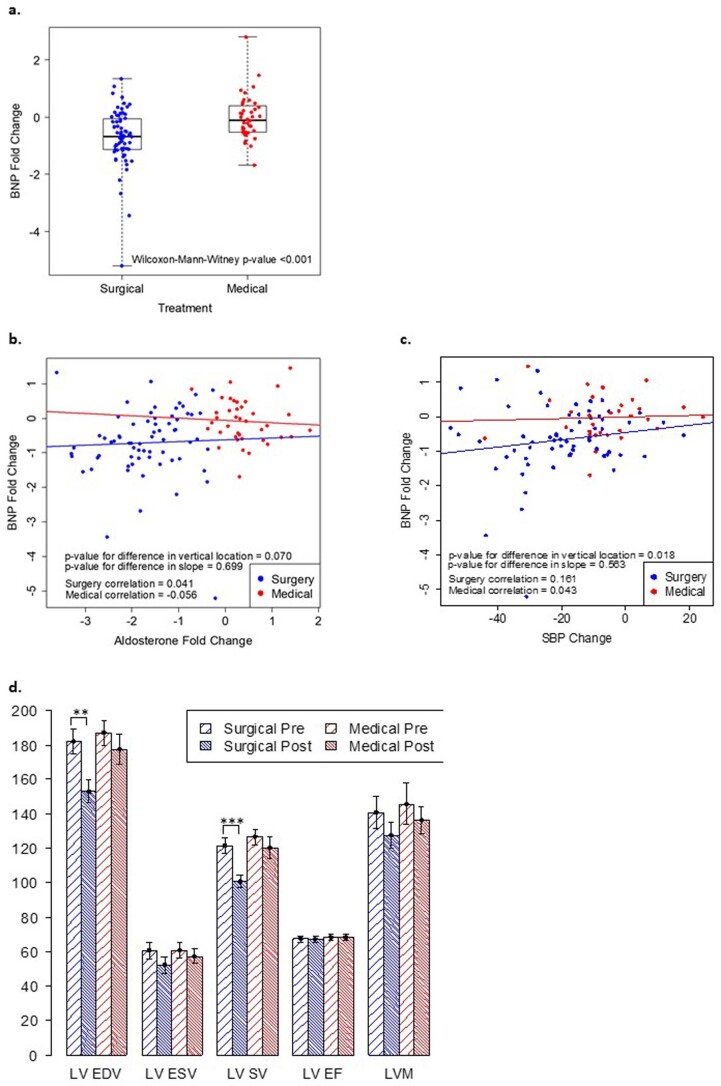
Cardiovascular Outcomes. **a**. Fold change in plasma BNP levels between baseline and 6 months post surgery or after 9-12 months of medical therapy. Surgery median = -0.69 (IQR -1.14,-0.06), Medical median = -0.10 (IQR -0.53,0.40), p = 0.00013. n = 68 surgical group, n = 40 medical group. The centre line of the box represents the median, the upper and lower boundaries of the box represent the inter-quartile range and the upper and lower whiskers represent the maximum and minimum values in the range respectively. **b**. Correlation between fold change in plasma aldosterone levels and plasma BNP levels, pre and post-treatment. **c**. Correlation between fold change in systolic BP and plasma BNP levels pre and post treatment. **For b. and c**., the p-value for the vertical location comes from the t value for the treatment variable in a linear model where BNP fold change is the dependent variable and treatment and **b**. aldosterone fold change or **c**. SBP are explanatory variables. A two-sided T-test was used. The p-value for the difference in slope comes from the t value for the treatment: **b**. aldosterone fold change or **c**. SBP change interaction variable in a linear model where BNP fold change is the dependent variable and treatment, **b**. aldosterone fold change and treatment: aldosterone fold change interaction or **c**. SBP change and treatment: SBP change interaction are explanatory variables. A two sided t-test was used. **d**. Left ventricular volumes, ejection fraction and mass were measured using CMR at baseline and at 6 months post surgery, or after 9-12 months of medical therapy. Y axis shows volume in mililiters for LV EDV, ESV and SV; % for LV EF and grams for LVM. Pre and post-intervention measurements are shown for each treatment group. 15.1% reduction in LV EDV and 15.8% reduction in LV SV is seen post surgery, while 5.6% reduction in LV EDV and 5.0% reduction in LV SV is seen after medical therapy. Two-sided Mann-Whitney-Wilcoxon tests: **p = 0.00186 ***p = 0.00037. n = 25. The bars represent the mean and error bars represent + /- 1 standard error from that mean. IQR, interquartile range; BNP, N-terminal pro-B-type natriuretic peptide. LV, left ventricular; EDV, end diastolic volume; ESV, end systolic volume; SV, stroke volume; EF, ejection fraction; LVM, left ventricular mass.

Plasma troponin (T and I) did not change following adrenalectomy (Extended Data Table 2). In contrast, plasma NT-pro BNP decreased by a median of 31% (Extended Data Table 2 and Extended Data Fig. 4a–c). This finding was consistent with the cardiovascular magnetic resonance imaging (CMR) sub-study, in which the end diastolic volume decreased by 15% following surgery, but only by 6% on medical therapy (Extended Data Fig. 4d). In contrast, the left ventricular mass decreased in both interventions.

### Predictors of outcome from surgery

**Fig. 4:**
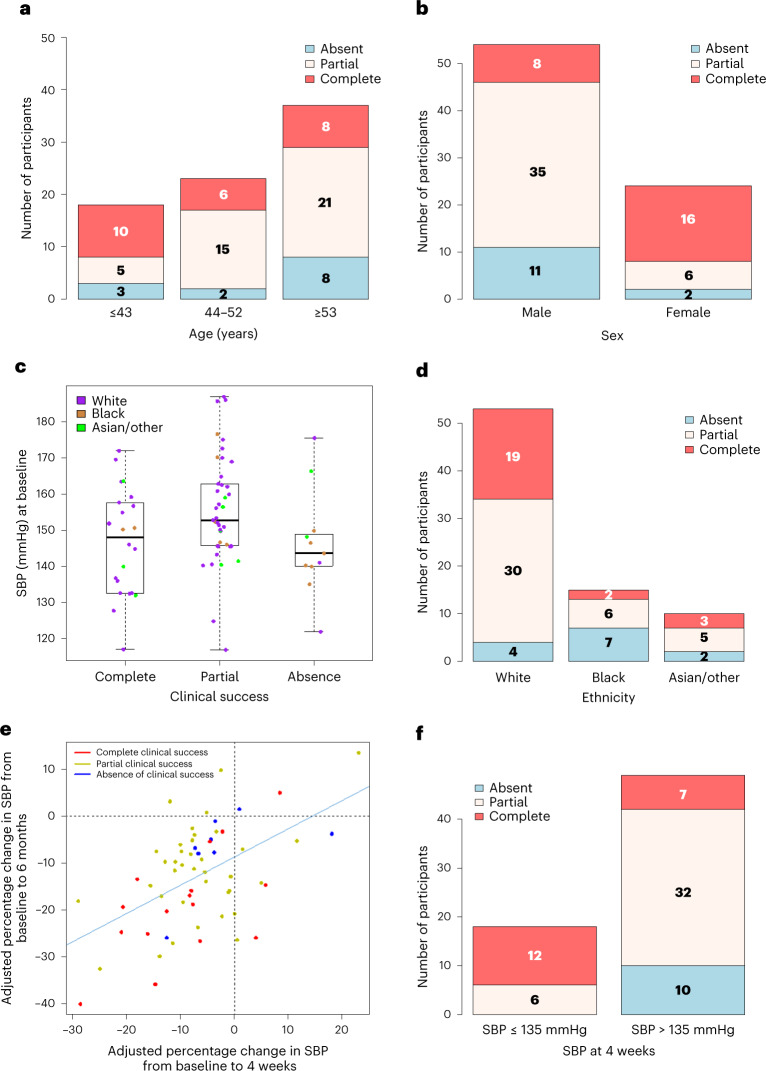
Predictors of clinical outcome from surgery. **a**–**d**, Influence of age (**a**), sex (**b**), baseline SBP (**c**) and ethnicity (**d**) on clinical outcomes. As seen from **a** and **b**, age and female sex are associated with a higher likelihood of complete clinical success. For age treated as categorical groups, statistical significance was determined by Fisher’s exact test (*P* = 0.065). For age treated as continuous, in univariate proportional odds logistical regression, the odds ratio was 0.43 (95% CI = 0.27 to 0.67) for an improvement of one level of outcome per 10 years of age (*P* = 0.00022). For sex, statistical significance was determined by Fisher’s exact test (*P* = 0.000036). For female versus male in a univariate proportional odds model, the odds ratio of going up one level of outcome was 9.078 (95% CI = 3.07 to 26.81) (*P* = 0.000065). *n* = 78. In **c**, the baseline SBP was lower in patients achieving complete or absent clinical success than in those achieving partial success. Each dot represents a single patient and is color coded to indicate the individual’s ethnicity. The center line of the box represents the median, the upper and lower boundaries of the box represent the interquartile range and the upper and lower whiskers represent the maximum and minimum values in the range, respectively. *n* = 78. For **d**, the absence of clinical success (shown in blue) was significantly more frequent in patients who were Black compared with other ethnicities. In a univariate proportional odds model, the odds ratio of going up one level was 0.14 (95% CI = 0.042 to 0.49). Statistical significance was determined by two-sided *t*-test (*P* = 0.002). **e**, Correlation between the decrease in SBP after 4 weeks of spironolactone therapy and the decrease in SBP at 6 months post-surgery (*r* = 0.53 (95% CI = 0.32 to 0.69) and *P* = 0.000012). The *P* value comes from a correlation test whose test statistic follows a *t* distribution. SBP is shown as an adjusted percentage change, which was calculated with Oldham’s correction^16^ (corrected change in blood pressure (%) = 100× the actual change in blood pressure divided by the average of the baseline and post-treatment pressure). *n* = 67. **f**, SBP after 4 weeks of spironolactone predicts clinical success. All patients whose SBP dropped to ≤135 mmHg after 4 weeks of spironolactone achieved either partial or complete clinical success. SBP ≤ 135 mmHg after 1 month of spironolactone was the best univariate predictor of complete clinical success with an odds ratio of 13.0 (3.72 to 45.24). Statistical significance was determined by two-sided *t*-test (*P* = 0.000057). *n* = 67.

Univariate analysis confirmed that young age, female sex and a lower starting blood pressure predicted patients who achieved complete clinical success (Fig. 4a–c)^14^. However, for systolic blood pressure (SBP), we observed that the starting pressure was similar in those with complete or absent success, but higher in patients with partial success (Fig. 4c). This paradox is probably due to the PASO definition of complete success being an absolute blood pressure (<135 mmHg), which is easier to reach from a lower starting point, whereas partial success is defined as a decrease in blood pressure (of ≥20/10 mmHg), which is easier to achieve from a higher blood pressure. Absence of clinical success occurred in 13 patients, the majority of whom (7/13; 54%) were Black and treated with a calcium channel blocker (Fig. 4c,d). Similarly, 21 of the 39 participants (54%) with absent biochemical success were Black patients.

### Spironolactone test

Two analyses were planned. The first quantified the correlation between a decrease in blood pressure from baseline to 4 weeks on spironolactone (aldosterone blockade) and the decrease in blood pressure from baseline to 6 months after adrenalectomy (Fig. 4e). The second evaluated a single measurement—SBP on spironolactone—as a binary predictor of whether surgical cure of hypertension would be achieved. In the first, quantitative analysis, we performed Oldham corrections of the two sets of blood pressure changes; this correction removes the intrinsic correlation between two changes from a common starting value^16^. The decrease in blood pressure after spironolactone correlated with a reduction in blood pressure post-surgery (*r* = 0.53 (95% CI 0.32 to 0.69); *P* < 0.001). A more useful clinical measure than this correlation value is the binary analysis (Fig. 4f) showing that patients whose SBP decreased to ≤135 mmHg on spironolactone always achieved clinical success, partial or complete, with 12/18 (67%) of these patients able to discontinue all antihypertensive medications. The blood pressure achieved on spironolactone reflects both the pre-spironolactone blood pressure (previously reported as a prediction of success) and the decrease during spironolactone therapy.

### Subtypes of APA identified by somatic genotype and IHC

Genotyping was performed in 70 CYP11B2-expressing adenomas. Bulk RNA sequencing (RNA-seq) of APAs and the paired adjacent adrenal tissue allowed the detection of known somatic driver mutations in most samples (Supplementary Table 4)^17^. The remainder were detected either by whole-exome sequencing (WES) or targeted sequencing of known mutation hotspots. In total, 62/70 (89%) samples had a mutation in the known hotspots of *KCNJ5*, *CACNA1D*, *ATP1A1*, *ATP2B3*, *CTNNB1, GNAQ* or *CLCN2*. Some of the *ATP1A1* deletions spanned different bases from those previously described^18^. The single *CLCN2* mutation detected was a deletion in four residues upstream of the three deleted in one of the germline families^19,20^. In two APAs without a known functional mutation, RNA-seq detected somatic mutations predicted to delete the function (SIFT score = 0) of, respectively, *VAPA* and *DPYSL2* (*CRMP2*), and showed both genes to be abundantly expressed in all samples of adjacent adrenal cortex. Both mutations were confirmed on genomic DNA analysis.

**Extended Data Fig. 5:**
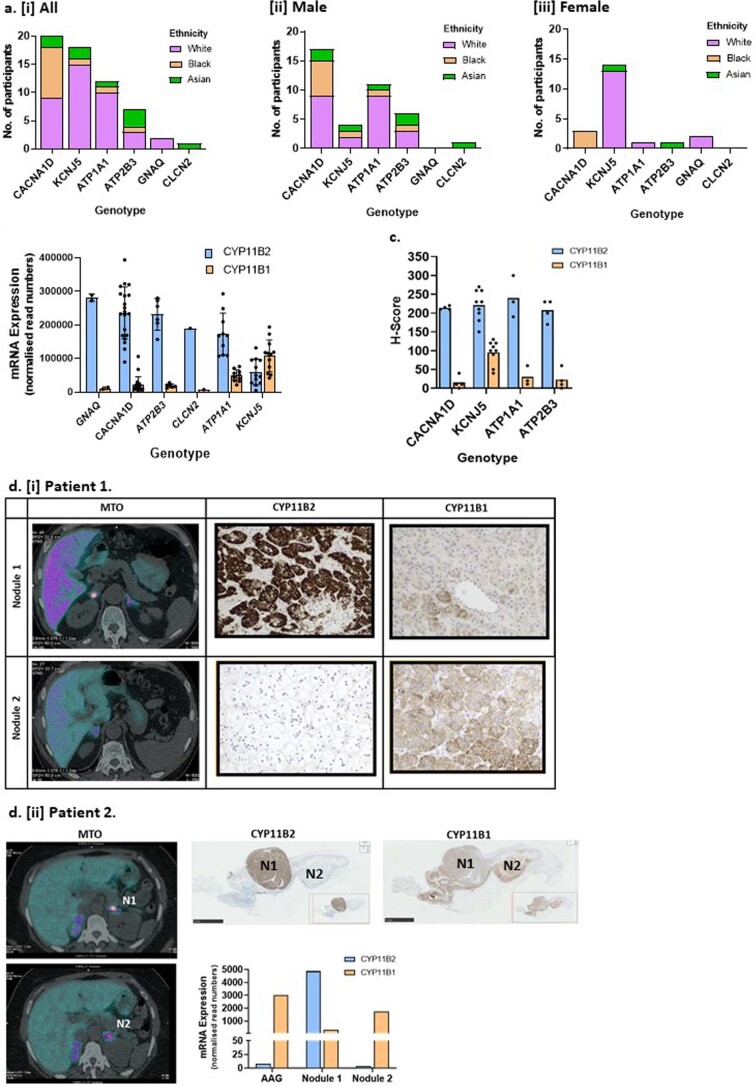
Sub-types of APA, phenotype-genotype correlations and immunohistochemistry (IHC). **a. Frequency of APAs harbouring each of the known somatic gene mutations**. **[i]** Each genotype was sub-divided to show variation in ethnicity, demonstrating significant differences between genotypes. n = 60. Fisher’s Exact test p = 0.0312. Genotyping by sex is shown in **[ii]** male, n = 39 and **[iii]** female, n = 21 patients. A correlation between ethnicity and genotype was seen in female (p = 0.0014) but not male patients (p = 0.3261). **b**. ***CYP11B1*** **and** ***CYP11B2*** **mRNA expression by APA genotype**. mRNA expression of APAs is the normalised read count on RNA sequencing. The bar represents mean and error bars show +/− 1 standard deviation from mean. For *CYP11B2* expression, *KCNJ5 v* other genotypes, Kruskal-Wallis test, p = 2.95×10^-7^; For *CYP11B1* expression, *KCNJ5 v* other genotypes, Kruskal-Wallis test, p = 2.0×10^-5^. Total with genotype and gene expression, n = 51. Numbers of each genotype are smaller than in Extended Data Fig. 5a, because not all adrenals had an RNAlater sample collected at the time of surgery for RNA sequencing. **c. Intensity of CYP11B1 and CYP11B2 expression of APAs on IHC staining was quantified using the H-score system**^69^. The average H-score for each genotype showed differences in CYP11B1 expression between *KCNJ5*-mutant and other APAs. ANOVA p = 0.0002; *KCNJ5 v CACNA1D* p = 0.0009, *KCNJ5 v ATP1A1* p = 0.0128, *KCNJ5 v ATP2B3* p = 0.0022. No differences in CYP11B2 expression on IHC was detected between genotypes. ANOVA p = 0.937. Paired comparisons were made using Tukey’s honestly significant difference test. Note small sample size within each group in above analysis (n = 3-9), although these did pass the Shapiro-Wilk test for normality. H-scoring only performed on APAs of known gene mutations from Cambridge University Hospital, total. n = 30. **d. Two illustrative cases demonstrating selective** **[**^**11**^**C]-metomidate uptake in patients with two or more adrenal adenomas. [i]** Patient 1 has two adenomas on their adrenal, shown in separate axial MTO images. The first nodule is [^11^C]-metomidate avid while the second is ‘cold’. IHC staining of the two nodules show strong positive staining for CYP11B2 in nodule 1 while nodule 2 is negative for CYP11B2. IHC staining for CYP11B1 is positive in both nodules. **[ii]** Patient 2 had multiple adrenal nodules, only one of which (nodule 1) demonstrated high [^11^C]-metomidate uptake on PET-CT (left hand panels). Top middle and top right hand panels show high power views of patient 2’s adrenal, with CYP11B2 and CYP11B1 IHC staining respectively. Bottom middle panel: RNA sequencing results showing increased mRNA expression for *CYP11B1* (compared to AAG) in both nodules, and increase mRNA expression for *CYP11B2* in nodule 1 only. Scale bar represents 5 mm. APA, aldosterone producing adenoma; AAG, adjacent adrenal gland; IHC, immunohistochemistry; NFA, non-functioning adenoma.

Overall, the numbers of *KCNJ5* and *CACNA1D* mutations were similar to each other, but there were differences in frequency between people of different ethnic groups (Extended Data Fig. 5a). *CACNA1D* mutations were much more common than *KCNJ5* in the APAs of Black patients, in agreement with previously reported findings^21^. A difference (from these findings) in our cohort was the rarity of *KCNJ5* mutations in both male and female Black patients (Extended Data Fig. 5a). Fourteen out of 18 patients with *KCNJ5* mutations and both patients with *GNAQ* mutations were women. All other mutations were found mainly in men.

The expression of CYP11B2 was slightly lower, and CYP11B1 substantially higher, in *KCNJ5*-mutant than other APAs, as previously noted^18,22–25^. This was apparent at the RNA level in 51 adenomas with somatic mutation of one of the five commonly mutated genes (Extended Data Fig. 5b). Immunohistochemistry (IHC) analysis showed similar differences, quantified as *H* scores, in a subset of APAs stained for both CYP11B1 and CYP11B2 (Extended Data Fig. 5c). In patients where MTO predicted a mixture of functional and non-functional adenomas, with respectively high and low [^11^C]metomidate uptake, IHC confirmed large differences in CYP11B2 expression between adjacent adenomas. This is illustrated for a pair of patients for whom CYP11B1 IHC was also available (Extended Data Fig. 5d).

**Extended Data Fig. 6:**
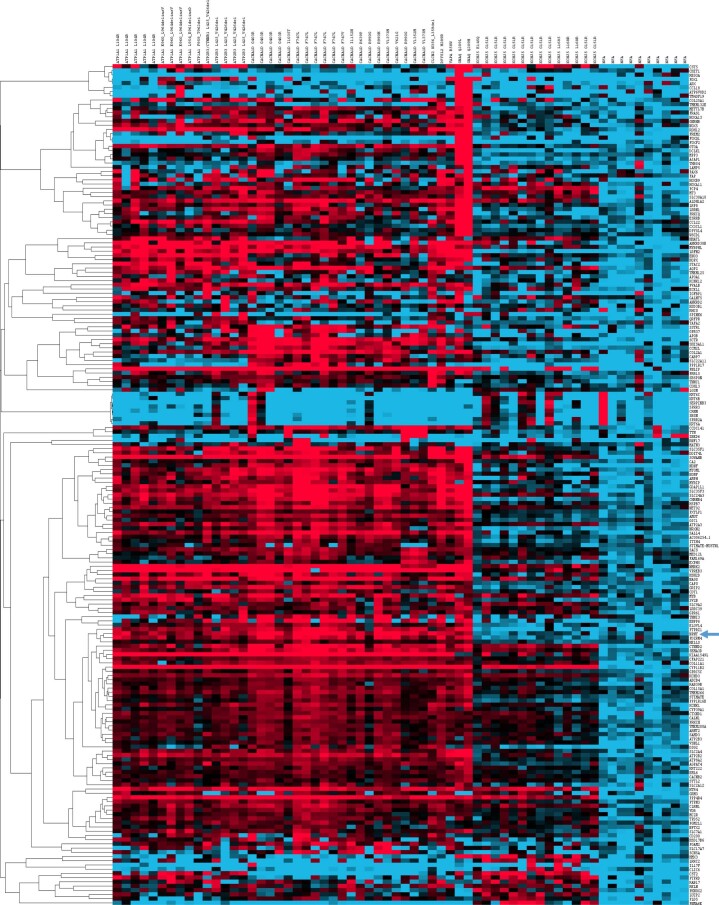
Gene expression profiles in aldosterone producing adenomas (APAs) and non-functioning adenomas (NFAs). Heat map representation of 200 most differentially expressed genes between adrenal adenomas and adjacent adrenal. Each column represents the expression profile of the APA or NFA. The unsupervised cluster analysis of samples separated the expression profile of APAs from NFAs, and between different genotypes. Red represents upregulation and blue represents downregulation of genes. Arrow shows the gene *nephronectin, NPNT* (*All text is legible by increasing zoom/magnification of file)*.

**Extended Data Fig. 7:**
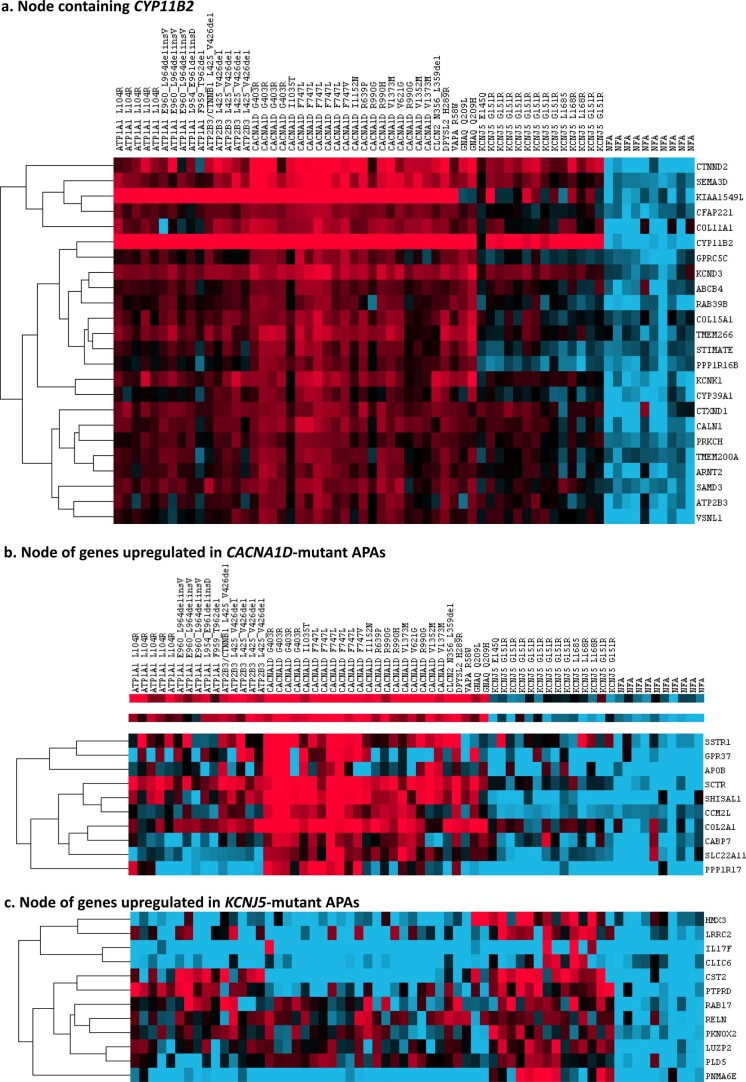
Heat map representation of differentially expressed genes between APAs and NFAs, and between genotypes. Each column represents the expression profile of the APA or NFA. Both genes and individual APAs are hierarchically clustered. Three nodes of interest are shown: **a**. node containing *CYP11B2*; **b**. node showing most upregulated genes in *CACNA1D*-mutant APAs. **c**. node showing most upregulated genes in *KCNJ5*-mutant APAs. The *CYP11B2* nodes includes several genes previously noted to be upregulated in APAs, for example *VSNL1, CALN1*. Genes which are significantly upregulated in APMs are also seen in this node: *TMEM266 (TMEM266 (C15ORF27), PPP1R16B, SEMA3D, KIAA1549L (C11ORF41), CFAP221 (PCDP1)*. Red represents upregulation and blue represents downregulation of genes. APA, aldosterone producing adenoma; AAG, adjacent adrenal gland; IHC, immunohistochemistry; NFA, non-functioning adenoma; APM, aldosterone producing micronodule (previously known and aldosterone producing cell clusters, APCCs).

RNA-seq enabled whole transcriptomes and genotypes to be directly correlated in the same samples and the results are shown for 200 genes that were the most upregulated in APAs (by at least 2.5-fold) compared with the adjacent adrenal. The results for all 200 genes are shown in Extended Data Fig. 6 and nodes of interest are shown in Extended Data Fig. 7.

The first part of the heatmap (Extended Data Fig. 7a), the *CYP11B2* node, includes several genes previously noted to be upregulated in APAs (for example, *VSNL1* and *CALN1*; refs. 22,26–29). Of additional interest are genes that have previously been reported as being substantially upregulated in aldosterone-producing micronodules (APMs; formally known as aldosterone-producing cell clusters) compared with the zona glomerulosa (ZG)^30^. The second part of the heatmap (Extended Data Fig. 7b,c) illustrates genes that we found to be selectively upregulated in APAs of some somatic genotypes compared with others. Most of the genes previously noted to differentiate *KCNJ5*-mutant from other, zona glomerulosa-like APAs, again vary many-fold in their expression between the two types. In particular, the expression of *nephronectin* (*NPNT*; Extended Data Fig. 6)—a gene that is selectively expressed in the normal zona glomerulosa compared with the zona fasciculata^18,24,31^—was low in *KCNJ5*-mutated APAs as they are usually composed of cells with zona fasciculata-like characteristics. It was also twice as abundant in *CACNA1D-* than *ATP1A1*- or *ATP2B3*-mutant APAs.

### Multivariate prediction, including genotype and urine steroid profile data

**Extended Data Fig. 8:**
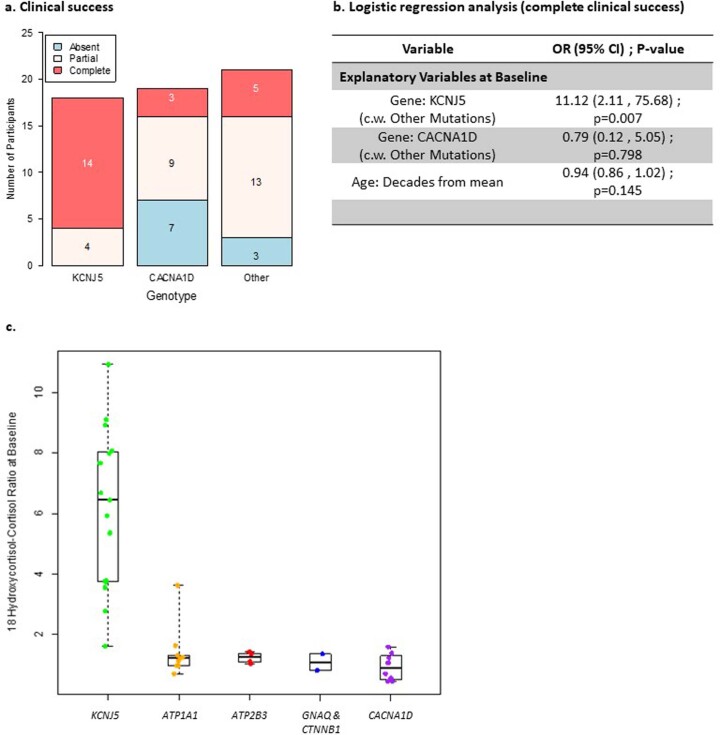
Predictions of clinical success. **a. Genotypes and clinical success associations**. Number of patients achieving each definition of clinical success by genotype. n = 58. Fisher Exact test p = 0.00021. *KCNJ5* v Other: Univariate Proportional Odds Model, Odds Ratio of going up 1 level 10.37 (95% CI (2.50, 42.99)) p = 0.00123. *CACNA1D* v Other: Univariate Proportional Odds Model, Odds Ratio of going up 1 level 0.38 (95% CI (0.11, 1.36)) p = 0.13703. **b. Predictors of complete clinical success**. Results from logistic regression analysis of variables predicting complete clinical success. Note the two patients with double *GNAQ/CTNNB1* mutations were excluded from this analysis since 16/16 patients with this double mutation were previously reported to be clinically cured^51^. p-values based on the t values from the proportional odds logistic regression c.w., compared with. **c. Urine steroid profile and genotype associations**. 18-hydroxycortisol/cortisol ratio collected at baseline are shown by genotype. A difference in 18-hydroxycortisol/cortisol ratio was seen between APA genotypes: Kruskal Wallis test p < 0.0001; *KCNJ5 v ATP2B3* p = 0.071, *KCNJ5 v ATPA1A* p = 0.003, *CACNA1D p* < *0.0001*. Paired comparisons were made using Bonferroni p-value correction. The center line of the box represents the median, the upper and lower boundaries of the box represent the inter-quartile range, and the upper and lower whiskers represent the maximum and minimum values in the range respectively. Due to small sample size *GNAQ* and *CLCN2* were excluded from the above analysis. n = 37.

All 18 patients with *KCNJ5* mutations achieved clinical success, which was complete in 14/18 (78%) patients—an odds ratio of 10.4 compared with other patients (Extended Data Fig. 8a). Biochemical success was complete in 17 patients, the other one having the rare p.Glu145Gln mutation. In contrast, the odds ratio for complete clinical success in patients with *CACNA1D* mutations, which occurred in only 3/19 (16%), was 0.38, while they contributed to 7/10 (70%) absences of clinical success and 3/5 (60%) instances of only partial biochemical success.

These results posed the question of whether the driver of these opposing outcomes for the two most common genotypes was a difference in the syndrome of PA caused by the genotype, or the differences in age, sex and ethnicity associated with the genotype. In the planned (binary) logistic regression of complete versus partial or absent clinical success on genotype, age, sex and ancestry, *KCNJ5* was the major dependent variable, with an odds ratio of 11.12 (Extended Data Fig. 8b). Conversely, *CACNA1D* genotype and ethnicity were difficult to separate as primary determinants of an absence of success. In a proportional odds model, in which all three clinical outcomes (complete, partial and absent) were considered against genotype, age, sex and ethnicity, only age was weakly significant, but the highest odds ratio, 7.84 (95% CI = 1.24 to 55.96) was for the post-hoc comparison of *KCNJ5* versus *CACNA1D* genotype (Supplementary Table 5).

Previous studies have shown that *KCNJ5*-mutant APAs are associated with high serum levels of hybrid steroids, which require a combination of enzymatic steps typically restricted to the zona glomerulosa (CYP11B2) and zona fasciculata (CYP11B1 and CYP17A1)^32–35^. In our study, urine multi-steroid profiling identified significantly higher ratios of the hybrid steroid 18-hydroxycortisol to cortisol in patients harboring *KCNJ5* mutations, allowing for an almost complete separation from other PA cases (Extended Data Fig. 8c). The only borderline-high result among the non-*KCNJ5* mutant group was from a woman with an *ATP1A1*-mutant APA, who achieved complete clinical success. Conversely, the lowest 18-hydroxycortisol-to-cortisol ratio in the *KCNJ5*-mutant group was in a woman with a p.Glu145Lys mutation, who did not achieve complete clinical success.

## Discussion

Our principal finding is that MTO following pre-treatment with dexamethasone enables non-invasive detection of unilateral APAs and is at least as accurate in the prediction of outcomes from adrenalectomy as the standard, invasive investigation, AVS. The high proportion (almost two-thirds) of patients in whom we found and cured unilateral PA highlights the underdiagnosis in usual practice of unilateral disease. However, only 23/78 surgical patients achieved home blood pressure readings of <135/85 mmHg off all treatment. The mechanistic studies within MATCH have indicated how complete clinical success may, in the future, be predicted in advance of surgery, and why in the remaining patients an intervention to remove the source of autonomous aldosterone production may still be preferable to chronic medical treatment.

In the 3–4 years between the end of our proof-of-concept study^13^ and the design of MATCH, experience of MTO in patients whose AVS had been non-diagnostic led to a hypothesis of superiority of MTO over AVS. However, clinical experience and retrospective analyses are susceptible to ascertainment and other biases. These potential study biases are avoided by conducting a prospective, within-patient comparison of both interventions, and MATCH also identified the probable source of clinical bias. More than one-third of patients in whom each investigation found unilateral PA were not diagnosed by the other investigation (Fig. 2b). This finding was unexpected and suggests that MTO and AVS might each be found to be falsely superior overall if tested in patients previously negative on the other test. In MATCH, there were many individual patients in whom MTO or AVS was superior in correctly predicting surgical success, but there was no overall superiority. Nevertheless there was a double dividend from finding so many patients who lateralized on only one of the tests. One was the higher than usual proportion of patients with PA who proceeded to adrenalectomy. The other dividend was that, in a within-patient study of categorical outcomes, it is the subset of patients with discrepant predictions by the two investigations who enable their accuracy to be compared. Low probability scores, in particular, by each investigation could only be evaluated if the other investigation in the same patient was scored as high probability and led to a surgical outcome. Both of these dividends increased our statistical power to discover differences in accuracy. After 2 years, therefore, when it was apparent that MTO and AVS were detecting similar numbers of patients with unilateral PA, we added a meaningful non-inferiority hypothesis. Its lower bound of −17% was pre-specified in the formal protocol amendment, and at clinicaltrials.gov, and justified by the many clinical advantages of an entirely safe, non-invasive investigation, which is guaranteed to yield a same-day result, independent of operator skills.

[^11^C]metomidate has been available for more than two decades^36,37^. However, it has not entered routine clinical practice because, in studies that did not use dexamethasone, its in vivo selectivity for CYP11B2 was not apparent^38,39^. In MATCH, we compared the in vivo uptake of [^11^C]metomidate into APAs, non-aldosterone-producing adenomas and the adjacent adrenal, correlating with measurements of *CYP11B1* and *CYP11B2* expression of each region by quantitative PCR or RNA-seq. It was clear that the uptake of [^11^C]metomidate is much lower in CYP11B1- than CYP11B2-rich regions of the adrenal (for example, see Extended Data Fig. 5d). The main limitation of [^11^C]MTO is its 20-min half-life, which requires synthesis by an on-site cyclotron. ^18^F ligands, with a 2-h half-life, are currently under evaluation (NCT04529018), with the potential for molecular imaging to become available in all PA centers^40^.

In the future, demand for intervention will outstrip supply as the proportion of PA diagnoses rises from <1% to 10% and beyond. A secondary objective of MATCH was therefore to prospectively evaluate predictive measures of success, and to improve on the predictive power of univariate variables (young age, female sex and lower baseline blood pressure)^41–43^. A further aim was to harness improved understanding of the molecular pathogenesis of APAs and their phenotypes into clinical decision-making. These objectives led to the nested spironolactone study, and to the genotyping of almost all APAs and the measurement of particular urine steroid metabolites as a predictor of genotype. The 13-fold greater likelihood of achieving complete clinical success following surgery, if SBP on spironolactone was ≤135 mmHg (Fig. 4f), probably reflects both the lower pre-treatment blood pressure in these patients and the contribution of aldosterone excess to this pressure.

The pre-specified molecular analyses build on the discovery of distinct genotype–phenotype patterns among the APAs, when the characteristics of either the APAs (for example, size, pathological appearance and transcriptome) or the patients (for example, age and sex) are compared between APA genotypes^18,22–25^. At the same time, the conventional anatomical subtype division into unilateral and bilateral has become more blurred^9,10^. So, the question arose as to whether some APA genotypes are more likely to be clearly unilateral, with others being asymmetric rather than unilateral. We are not the first to associate *KCNJ5* mutations with better clinical outcome following adrenalectomy, or with hybrid steroid excess^44–46^. However, the observation has not been uniform. In studies that are retrospective and/or in which the control group is wild-type APAs, it is more difficult to eliminate confounders, such as non-functioning adenomas masquerading as APAs, or to collect data of equal weight from all cases. The 78% complete clinical success rate for *KCNJ5*-mutant APAs, compared with 31% overall (Extended Data Fig. 8a) and only 15% once *KCNJ5*- and *GNAQ/CTNNB1*-mutant APAs are excluded, is dramatic. Those achieving complete clinical success included males and females and older and younger patients. The paucity of young Black women with *KCNJ5*-mutant APAs in MATCH partially explains why our complete clinical success rate of 31% was at the lower end of the anticipated 30–60% range^11^. The observation also makes it unlikely that good outcomes in patients with *KCNJ5*-mutant APAs are due to earlier detection of hypertension during obstetric or contraceptive consultations.

*CACNA1D* mutations are the dominant somatic genotype of APMs, which are the main site of aldosterone production in at least 30% of adult adrenals—even those of normotensive people^30,47,48^. Unlike the discrete hotspots for somatic mutation in all other genes causing autonomous aldosterone production, >60 mutations have been reported in *CACNA1D* since our first report of seven in 2013, with up to four different mutations in the same adrenal^18,25,48^. *CACNA1D*-mutant APMs may also underpin IHA, and before this paper, *CACNA1D* was already reported as the dominant mutation (rather than *KCNJ5*) in the APAs of Black male patients^21,48,49^. We infer that the high rate of absence of success in Black patients may reflect asymmetric rather than unilateral PA, and this is presaged by a report of *CACNA1D* mutations in 6/10 APAs from Paris, whose removal did not cure PA^50^. Some evidence from MATCH is that 4/5 patients who achieved partial biochemical success were Black. In these patients, renin levels remained suppressed despite aldosterone levels decreasing by >75%, to <300 pmol l^−1^. Conversely, *KCNJ5* mutations and the *GNAQ/CTNNB1* double mutations typically arise in solitary adenomas and are rarely seen in APMs^25^. To date, 16/16 double-mutant APAs have achieved complete clinical success^51^.

In most endocrine conditions, it is normalization of the culprit hormone that is the main goal of intervention, and in MATCH this was achieved irrespective of genotype. Furthermore, the large decrease in plasma NT-pro BNP adds weight to the recommendation for intervention as a means of cardiovascular protection (Extended Data Fig. 4a and Extended Data Table 2). Although NT-pro BNP was a secondary outcome of MATCH, and is not included among PASO outcomes, it is important to recall that guidelines cite the substantially increased cardiovascular risk of PA, rather than difficulty in controlling hypertension, as the primary reason to recommend adrenalectomy^6,11^. The failure of NT-pro BNP to decrease in the patients receiving medicine could suggest that aldosterone suppression is more effective than mineralocorticoid receptor blockade, with aldosterone able to stimulate non-mineralocorticoid receptor-mediated effects^52,53^. However, the allocation to surgical and medical management was not randomized, and this hypothesis should wait for formal randomized, controlled trials (RCTs) comparing drugs in the aldosterone synthase inhibitor and mineralocorticoid antagonist classes.

Prospective studies in PA are rare, and all potential designs for MATCH had limitations. We drew on the experience of designing complex crossover trials of antihypertensive therapies that have influenced guidelines^54,55^.

A first limitation was that, by evaluating MTO against predicted biochemical and clinical outcomes (rather than against the criterion-standard investigation of AVS, as in our 2012 feasibility study), the accuracy of MTO could only be assessed in the surgical group. In practice, accuracy within these patients is more important than in the medical group since a wrongly removed adrenal cannot be restored.

Second, an RCT is often regarded as the gold standard, in which each patient has one or other intervention, but not both. However, an RCT would have been unlikely, in 2016, to stratify by ethnicity or the somatic mutations of yet-to-be diagnosed APAs. Most importantly, we would not have learned, in an RCT, that many APAs are diagnosed by only one of the investigations, and that consequently about one-third of patients going to adrenalectomy in each arm would differ by unknown factors. Conversely, a perceived limitation of within-patient studies is so-called carryover between interventions. As described in the Methods, we were rigorous in always scoring the research intervention (namely, MTO) before the AVS result was revealed. In only seven patients did a composite interpretation lead to the multidisciplinary team (MDT) decision, and none of these cases could favor MTO or AVS: either the two investigations lateralized to opposite adrenals and surgery was, by protocol, deferred or both investigations scored medium and surgery was performed on clinical grounds.

Third, the study population in MATCH was more ethnically diverse than in previous studies, but our eligibility criteria might still have influenced the representation of different ethnic groups. The higher than usual number of patients (44%) who met the Endocrine Society trinity of high aldosterone, suppressed renin and hypokalemia, and the requirement for an abnormal adrenal on CT, potentially excluded patients with a milder phenotype^11^. However, the percentage of patients with hypokalemia was inflated by meticulous searching of patients’ hospital and primary care records for evidence of previous hypokalemia. Only 42 of all 143 recruited patients (29%) were hypokalemic at screening. Furthermore, our finding of fewer *KCNJ5* than non-*KCNJ5* mutations suggests that our eligibility criteria had minor influence, if any. APAs with *CACNA1D* mutations are typically smaller than *KCNJ5-*mutant APAs, so easier to miss on CT imaging^18,22,35,56^. Yet, 30% of patients in MATCH were Black, of whom only one had a classical *KCNJ5-*mutant APA. Patients without an APA, whose unilateral PA is presumed due to APMs, would not be detected by MTO. Such patients, whose benefit from surgery is reduced^57^, will continue to have AVS if their CT is regarded as normal.

The final design question concerned the use of cosyntropin stimulation during AVS. The advantages and drawbacks of this are discussed in the Endocrine Society guidelines and expert consensus statement^11,15^. A debate in 2019 (too late to influence our design) found strong arguments both for and against the use of cosyntropin^58^. The most contemporary papers have slightly favored cosyntropin use^59,60^, but in study design we were concerned more with consistency between centers than with whether cosyntropin or no cosyntropin was superior.

The primary objective of comparing the accuracy of MTO with AVS was limited to outcomes after adrenalectomy, using the PASO criteria for surgical success^14^. These criteria have not been validated for medical outcomes. As a within-patient study, MATCH was not designed to compare overall medical and surgical outcomes between patients in whom only one of MTO or AVS detected a high probability of unilateral disease. It is theoretically possible that one of these two subgroups is higher risk and benefits more than the other from surgical intervention. It will be open to clinicians wishing to replicate MATCH’s high probability of unilateral PA to undertake a second investigation in selected patients for whom surgery would be of high benefit:risk. However, we anticipate that the main impact of MATCH will be a gradual increase in the proportion of surgically to medically treated patients as molecular imaging becomes more available.

In conclusion, our study validates dexamethasone-suppressed MTO as a CYP11B2-selective investigation for lateralizing PA. MATCH shows that it will now be possible to diagnose unilateral APAs when AVS is either unavailable, unsuccessful or not desired by the patient. Unlike AVS, MTO is quick, safe and reliable. These advantages may attract more clinicians and patients to seek a diagnosis, especially if the ^18^F analog becomes available and transportable to most hospitals with PET imaging facilities^40^.

The longer-term impact of MATCH may be a reassessment of who undergoes lateralization and surgery, and a stimulus to decision-making by molecular, rather than anatomical, diagnosis. In this view, either surgery itself (a procedure usually undertaken for cure rather than prevention) or the counseling of patients to whom it is offered, will emphasize its success rate in patients with elevated hybrid steroid secretion and normal blood pressure on spironolactone. Conversely, patients advised of a <1/10 likelihood of achieving complete clinical success based on demographics, steroid secretion and response to spironolactone may now prefer optimized medical treatment and/or to wait for nodular ablation if or when this is shown to decrease blood pressure, medication and plasma NT-pro BNP as much as surgery. When that time arrives, molecular imaging offers the potential advantages over AVS of localization as well as lateralization, given our examples of patients in whom the largest nodule on CT was not the [^11^C]metomidate-avid, CYP11B2-positive adenoma.

## Methods

### Study design

MATCH was a within-patient, multicenter prospective study designed to compare the accuracy of MTO with that of AVS in predicting unilateral PA. Patients were recruited from three study sites in the United Kingdom: St Bartholomew’s Hospital, Queen Mary University of London (SBH); Addenbrooke’s Hospital, University of Cambridge (CUH); and Guy’s and St Thomas’ Hospital (GSTT). The study was conducted in accordance with principles of the Declaration of Helsinki. The study protocol was approved by the National Research Ethics Service (Dulwich Research Ethics Committee; Integrated Research Application System project ID 189508) and the Health Research Authority and Administration of Radioactive Substances Advisory Committee and registered on ISRCTN (identifier 91387205; 10.1186/ISRCTN91387205) and on ClinicalTrials.gov (identifier NCT02945904). In addition, the study protocol was approved by the local research and development department at each participating center.

### Participants

Participants were recruited through tertiary endocrinology clinics at SBH and CUH or complex hypertension clinics at SBH and GSTT. The criteria for inclusion consisted of patients aged 18 years or older with a probable or definite adenoma on CT or MRI scans of the adrenals (defined below) and meeting the Endocrinology Society criteria for diagnosis of PA^11^ (elevated ARR measured off interfering medications and either: (1) a plasma aldosterone >190 pmol l^−1^ after a 2 l 0.9% saline infusion test; or (2) failure to suppress plasma aldosterone by 30% plus persistent plasma renin suppression following a 25 mg captopril challenge test; or (3) spontaneous hypokalemia plus plasma renin below detection levels plus plasma aldosterone >550 pmol l^−1^). Patients with elevated ARR could be put forward for consideration by the MDT for inclusion in the study as exceptional cases in whom a confirmatory test was not performed if plasma aldosterone was >450 pmol l^−1^ and: (1) plasma renin was <0.5 pmol ml^−1^ h^−1^ (<9 mU l^−1^) if measured on treatment with an ace inhibitor (lisinopril ≥ 20 mg or equivalent) or an angiotensin receptor blocker (losartan 100 mg or equivalent); or (2) if the patient was aged <40 years and a definite adrenal adenoma was seen on CT or MRI.

There was no minimum dimension used to classify an adenoma. For lesions above 10 mm, CT or MRI is able to outline the nodule and measure its CT density reliably as well as evaluate the loss of signal on out-of-phase images (MRI). For nodules below 10 mm, subjective visual criteria were applied and included: a focal increase in the caliber of an adrenal limb or body resulting in distortion and lobulation of the outer adrenal contour; and adrenal tissue adjacent to the nodule normal in both dimensions and contour.

Exclusion criteria included individuals who: (1) were unlikely to proceed with surgery if recommended; (2) were contraindicated for spironolactone/eplerenone therapy use; (3) were unable to stop beta blockers or direct renin blockers; (4) were pregnant women or unable/unwilling to take secure contraceptive precautions while undergoing investigations; (5)were unable/unwilling to take the dexamethasone required to prepare for an MTO scan; (6) were unwilling/unable to have both MTO and AVS; or (7) had a condition or drug regimen that was considered a contraindication by the principal investigator.

All eligible individuals gave written informed consent to participate in the study. Demographic data were also collected at the screening visit. This included age, sex, weight, height and ancestry. Ancestry was self-declared by individual participants using the list of ethnic groups for England and Wales, as defined by the Office for National Statistics^62^ (date accessed: 24 October 2022). Due to the small number of patients in each subgroup, Asian includes Asian or Asian British, Black includes Black, Black British, Caribbean or African, mixed includes White and Black Caribbean, White and Black African, White and Asian any other mixed or multiple ethnic background and other includes Arab and any other ethnic group.

### AVS and scoring

All AVS procedures were performed by one of three experienced interventional radiologists. Intravenous cosyntropin (50 μg h^−1^) was started 1 h before the procedure. Interpretation of the AVS results was in line with a recent consensus statement^15^. First, cannulation was considered successful if the cortisol level in each adrenal vein was ≥3× greater than that in the iliac and/or infrarenal inferior vena cava. A high probability of unilateral PA was diagnosed if the aldosterone/cortisol ratio in one adrenal vein was ≥4 times that in the contralateral adrenal vein^15,63^. A low probability of unilateral PA was diagnosed with an aldosterone/cortisol ratio of <3, which was indicative of bilateral aldosterone secretion. An aldosterone/cortisol ratio of 3–4 was considered intermediate. In the event of one or both adrenal veins not being successfully cannulated, the outcome was declared failed. Although in clinical practice it has been suggested that a focal right adrenal lesion in association with an aldosterone/cortisol ratio in a unilaterally cannulated left adrenal vein of less than half that of the infrarenal inferior vena cava/iliac vein can provide justification for a right adrenalectomy^64^, this was not adopted in MATCH. A breakdown of successful cannulation and lateralization is reported in Supplementary Table 6.

### [^11^C]metomidate synthesis, scanning and scoring

[^11^C]metomidate was manufactured in compliance with good manufacturing practice using a General Electric Medical Systems PETtrace cyclotron. [^11^C]methyl iodide was passed through a solution of (*R*)-methyl 1-(1-phenylethyl)-1*H*-imidazole-5-carboxylic acid in anhydrous dimethylformamide, containing tetrabutylammonoium hydroxide as a catalyst, and loaded directly into the injector loop of a GE Healthcare TRACERlab FX C system. This captive solvent methylation method produced [^11^C]metomidate with a radiochemical purity of >99% and a specific activity of 19.8–414.8 GBq µmol^−1^.

All patients received 0.5 mg dexamethasone orally four times a day for 72 h before MTO scanning. PET-CT imaging was performed on a GE Discovery PET/CT 690 scanner (GE Medical Systems). Non-contrast CT images were acquired over the adrenals (140 kV; 30 mA; slice thickness = 3.75 mm). Following an intravenous injection of [^11^C]metomidate (mean: 215 mBq; range: 86–289 MBq), dynamic PET images were acquired for 30 min at 30 min after administration. The images were reconstructed with iterative reconstruction (ordered subset expectation maximization) using two iterations, 24 subsets and a Gaussian filter of 6.4 mm. The reconstruction included time-of-flight, attenuation, scatter and decay corrections. The images were converted to standardized uptake values (SUVs; g ml^−1^) by dividing the activity concentration in the image voxels (Bq ml^−1^) by the injected activity per patient weight (Bq g^−1^).

All MTO scans were analyzed and initially scored by a single experienced radiologist blinded to the AVS result. Three key features were used to determine the probability of unilateral PA: a focal adrenal nodule with Hounsfield units in keeping with a benign adrenocortical adenoma; uptake of [^11^C]metomidate into the identified nodule; and, in keeping with our previous report, a calculated ratio of tumor maximum SUV (SUVmax) to normal background SUVmax of >1.25 (ref. 13). An opinion was formed on the basis of the presence or absence each of the three features described, and a score of high, intermediate or low probability of unilateral PA was assigned.

### Minimization

All patients underwent both AVS and MTO. The order that each participant underwent each investigation was determined by a minimization program within the study data center (Robertson Centre for Biostatistics, University of Glasgow), designed to maintain a balance with respect to study site, sex and age (<55 or ≥55 years). Both AVS and MTO were performed with the patient taking non-interfering medications such as doxazosin, verapamil and hydralazine, according to Endocrine Society consensus guidelines^11^.

### Clinical decision-making

Each study patient was discussed in an MDT meeting attended by principal investigators, radiologists and a surgeon. The MTO scan was presented and interpreted by the reporting radiologist, always without previous knowledge of the AVS results. For each investigation, the question posed was: ‘In the absence of any other information, would this result provide sufficient evidence for a referral for surgery?’. The MDT decision was not bound unconditionally to the criteria for diagnosis of unilateral PA as outlined above. The probability of unilateral disease could, by consensus, be reduced if data from the contralateral adrenal suggested bilateral disease (for example, adenoma in the contralateral adrenal with SUVmax higher than in the adjacent adrenal). Importantly, the agreed score for MTO was entered into the case report form before discussion of the AVS and could not be revised once the AVS results were revealed. The overall outcome of the MDT was a recommendation for either surgical or medical management. Surgery was recommended if either investigation was scored highly. In a small number of patients for whom both investigations indicated an intermediate probability of unilateral PA, surgery was recommended if there was a strong clinical indication to proceed. A decision for recommending surgery was deferred if MTO and AVS showed discordant results (lateralized to opposite sides). Such participants (two in total) were managed medically with a view to reviewing the results at the end of the study, when recommendations for surgery would be based on the outcomes from the study.

### Baseline visit and initiation of spironolactone therapy

After the second investigation, patients attended for measurements of all clinical and biochemical parameters for which a response to treatment would assess the accuracy of the MTO and AVS. Obtaining the baseline measurements at this point in the study allowed a maximum interval between the discontinuation of any interfering medicines being taken at screening and the opportunity for the blood pressure response to initiation of spironolactone to be assessed as a predictor of response to surgery.

After 4 d of home blood pressure recordings (three readings twice daily; 24 in total), blood was drawn for electrolytes, renin activity and/or renin mass, aldosterone, NT-pro-BNP and troponin. On the rare occasion that the minimum number of home blood pressure recordings was not available, office blood pressure recordings (an average of three sequential readings) were used instead. Participants also performed a 24-h urine collection for urine steroid profiles. Participants then commenced spironolactone 50 mg once daily for 2 weeks, increasing to 100 mg once daily depending on side effects and any concerns about renal function or potassium homeostasis. The response to spironolactone was recorded at 2 and 4 weeks after initiation of spironolactone with both home and office blood pressure recordings. Patients unable to tolerate spironolactone were treated with 25 mg eplerenone twice daily for 2 weeks, increasing to 50 mg twice daily.

### Surgical group

In patients referred for surgery, spironolactone/eplerenone therapy was continued, as tolerated, until the day of operation. Laparoscopic adrenalectomy, using standard operative techniques, was performed by a single endocrine surgeon at two of the participating sites, unless the patient or referring physician preferred local referral. The serum cortisol value at the conclusion of the 72-h preparatory dexamethasone before the MTO scan was used to guide the clinical concern about potential post-operative adrenal insufficiency. Where appropriate, supplementary hydrocortisone was provided and withdrawn after subsequent clinical and biochemical reassessment. The outcomes from surgery were assessed biochemically and clinically at 3 and 6 months after surgery, and at 12 and 24 months in patients recruited in earlier years of the study. At each visit, the same measurements were performed as at the baseline visit, except that NT-pro BNP, troponin and 24-h urine measurements were repeated only once, at the 6-month post-surgery visit.

### Medical group

After 4 weeks of spironolactone/eplerenone, further treatment titrations in the medical group were at the discretion of the study or referring physician. Formal study visits occurred at 9–12 months and 24 months, where the same clinical and biochemical measurements as at the baseline visit were performed, except BNP, troponin and 24-h urine measurements, which were repeated only once, at the 9- to 12-month visit.

### Outcomes

Primary outcome measures were assessed at 6 months post-surgery or after 9–12 months of medical therapy. The primary objective of MATCH was to compare the accuracy of MTO and AVS in diagnosing unilateral PA, by measuring normalization of biochemical and clinical parameters following adrenalectomy. The criteria for normalization were the PASO criteria, agreed by international consensus in 2017 (Supplementary Table 1) for each of partial or complete biochemical and clinic success^14^. Since the rank order of partial and complete success of PA and hypertension could be assumed (with more patients achieving biochemical than clinical success), prospective studies in PA lend themselves to hierarchical analysis of each PASO outcome, in which all four are primary outcomes without needing to adjust for multiple statistical comparisons (Fig. 1b). It is important to note that, whereas partial success may seem a softer version of complete success (either clinical or biochemical), it measures different parameters. Partial responses are changes in biochemistry, blood pressure and the defined daily dosage of medications, while complete responses are numbers below an absolute target. Of note, the first outcome in the hierarchy—partial biochemical success—is the sole outcome driven by reduction in the culprit hormone, aldosterone, to which excess cardiovascular risk in PA is attributed.

Secondary objectives of MATCH included serial measurements, up to 2 years post-intervention, of the criteria for assessing biochemical and clinical success, analyzed separately in the surgically and medically treated participants, and analyses of parameters that may, in the future, enable more reliable prediction of which patients can achieve complete clinical success post-adrenalectomy. The main clinical test was a within-trial measurement of participants’ responses to 4 weeks of treatment with spironolactone. Molecular analyses included RNA-seq of APAs for genotyping and transcriptomics. We also report the left ventricular mass and end diastolic volume measurements from the CMR sub-study of 50 patients, performed at baseline and 1 year after surgical or medical management.

### Statistical analysis

The primary analysis was a comparison of the accuracy of MTO and AVS. Each test was deemed accurate if it recommended surgery and surgery resulted in a cure (according to each PASO criterion), or if the test did not indicate surgery and surgery did not result in a cure. Since surgery was almost exclusively undertaken when one or both tests indicated that surgery was required, for those patients for whom neither test indicated surgery, both tests were treated in the analysis as having equal accuracy. Only in those patients for whom the two tests gave conflicting recommendations was it possible to determine which of the two test was accurate and which was not.

The difference in accuracy between the two tests was estimated among those who underwent surgery using the Newcombe–Wilson score method for paired binary data and tested using an exact McNemar test.

The sample size for the study was estimated from a table of all permutations of outcomes from each investigation and surgery, in which the frequency of each outcome reflected experience at participating centers (Supplementary Table 7). This estimated a need for 128 completed patients, with half undergoing surgery and half of those (32) doing so as the result of one of MTO or AVS, but not both. This sample size was based on having 90% power at a 5% significance level to detect a difference in accuracy between the two tests, assuming that on those occasions when only one of the tests was accurate, it would be MTO that was accurate 80% of the time. An initial sample size of 142 patients was defined, allowing for a 10% dropout rate, anticipated to be patients who declined either to have both investigations or to proceed with surgery even if lateralized.

The sample size was revised in November 2019 when it became apparent that the use of two investigations increased the proportion of participants with detectable unilateral PA, from the expected 50% to 66%. The dropout rate was much lower than 10%. The resulting smaller proportion of participants managed medically risked inadequate power for several secondary analyses (comparison of baseline characteristics and outcomes in the surgical and medical groups, and whether outcomes were sustained with time). The recalculated overall number of recruits anticipated to maintain the size of the medical group, and duration of follow-up, was 150. Still allowing for a 10% dropout rate, the revised total sample size was calculated to be 165.

Rather than reduce the margin of superiority of which increased numbers would permit detection, the larger sample size permitted declaration of a non-inferiority margin. A total of 150 completed patients, with at least 75 proceeding to surgery, would have 90% power to demonstrate non-inferiority of MTO versus AVS within a non-inferiority margin of −17%. The addition of this non-inferiority margin is specified in the protocol and statistical analysis plan and was published on clinicaltrials.gov after approval by the Dulwich Ethics Research Committee and Medicines and Healthcare products Regulatory Agency.

We had a qualitative and quantitative basis for the 17% margin. Regulatory advice is that bounds in equivalence and non-inferiority studies reflect ‘the largest difference that is clinically acceptable, so that a difference bigger than this would matter in practice’^65^. MATCH compared a 100% safe non-invasive procedure with (in principle) 100% technical success (no operator dependence) and an invasive procedure that requires a day off work, causes severe pain and hospitalization in 1–3% of patients, is available with a >50% success rate in few hospitals, and in all but the best exposes patients to potentially 5× more radiation than MTO when adrenal veins are difficult to locate^66^. If MTO were the established procedure, and AVS were the innovation, regulators would require much higher (>17%) accuracy for AVS than MTO to justify all of the downsides of AVS. In a numbers-needed-to-treat analysis, one in every six patients correctly diagnosed by AVS would need to be wrongly diagnosed by MTO. If the numbers needed to treat were larger than six (superiority ≤ 17%), the downsides of AVS would be too great for approval.

The full analysis set was defined as all patients who had both investigations. Because of pandemic-associated interruptions to the study, particularly the availability of laparoscopic adrenalectomy, the study was censored when more than 75 patients had undergone surgery and at least one evaluable outcome visit. The analysis is reported for the 128 patients who had reached a primary outcome visit either 6 months post-unilateral adrenalectomy (including five for whom data from their most recent, 3-month visit was carried forward) or 9–12 months after their baseline visit in the medically treated patients.

A benefit of the delayed completion of MATCH was that the 25-participant extension could be revised to include a within-patient comparison of MTO and AVS with an ^18^Fluoro analog of metomidate (^18^F-CETO), which had recently completed phase 1 evaluation. This extension (ClinicalTrials.gov identifier NCT04529018) is expected to be reported on in 2023.

### Secondary analyses

Secondary (and primary) outcomes were compared between patients who underwent surgery and those who did not and, for those who underwent surgery, between those for whom surgery was indicated by AVS only, MTO only or both tests.

Groups were initially compared using Fisher’s exact test or Kruskal–Wallis test, as appropriate. Linear, binary logistic or proportional odds logistic regression models were then used, adjusted for age and sex. These models were extended to investigate other predictors of outcome after surgery, in the subset of patients who underwent surgery. There was particular interest in blood pressure at baseline and 4 weeks, age, sex, ethnicity, the number of classes/defined daily dose of antihypertensive medications at baseline, tumor genotypes and immunohistochemical classification, contralateral suppression, numbers of nodules, blood biomarkers, SUVmax and the 24-hour urine steroid profile.

For each of the primary outcomes, a sensitivity analysis was carried out to assess the impact of including patients for whom AVS was classified as failed. A bootstrap procedure was applied, sampling with replacement from the 78 patients who underwent surgery. Sampling was stratified by whether AVS was successful or failed. For each sample, the estimated difference in accuracy between MTO and AVS was calculated for the combined sample of 78 patients and for the 67 patients for whom AVS had not failed, and the difference between these estimated differences was taken as a measure of the sensitivity of the overall result to the inclusion of AVS failures. One million replicates were drawn and a 95% CI was derived by the percentile method. If the 95% CI excluded zero, the overall result was judged to be sensitive to the inclusion of AVS failures. If the 95% CI included zero, the results were judged to be insensitive to the AVS failures.

Clinical data were analyzed using the 64 bit version 4.1.0 of R. Experimental data were analyzed using GraphPad Prism Version 9.

### Sub-study of the effect of spironolactone on MTO PET-CT results

One disadvantage of AVS is the need for patients to come off interfering medications before MTO PET-CT investigation. Our hypothesis was that spironolactone therapy at the time of MTO would not prevent the detection of unilateral disease. A sub-study was designed in which six patients underwent MTO scanning on two separate occasions: once having discontinued spironolactone (as part of the standard protocol); and on a separate occasion while taking at least 50 mg spironolactone. The scans were graded blinded to the knowledge of spironolactone status and compared. The sub-study was the same size as that which validated dexamethasone pre-treatment in our 2012 study, and slightly larger than the number of patients (four) in whom 25 mg spironolactone was reported to cause only slight de-suppression of plasma renin^67^.

### CMR sub-study

A CMR sub-study was performed with imaging acquired at baseline before treatment allocation in 76 patients, and at follow-up 12.0 ± 3.1 months later in 51 patients. All CMR imaging was performed at 1.5 Tesla (Aera, Siemens Healthcare). Cine imaging was performed using steady-state free precession sequences. Automated contouring of left ventricular blood and myocardial volumes using a clinically validated artificial intelligence analysis platform (with verification by two experts) enabled calculation of left ventricular mass, chamber volumes and ejection fraction.

### Severe adverse events

All severe adverse events (SAEs) were reported within 24 h to the sponsor, and locally to the research and development office for the relevant study site. Principal investigators were responsible for determining whether SAEs were study related. All SAEs were recorded in the electronic case report form and the research and ethics committee annual report and discussed at steering committee meetings.

### Biochemistry methods

All biochemistry tests were analyzed at United Kingdom Accreditation Service-accredited clinical laboratories. Serum aldosterone was measured by automated chemiluminescence immunoassay at SBH and GSTT (LIAISON; DiaSorin) and by tandem mass spectrometry at CUH (in-house method adapted from ref. 68). The direct renin mass was measured by automated chemiluminescence immunoassay at all three centres (LIAISON; DiaSorin). Before 3 February 2017, the plasma renin activity was assayed in EDTA plasma, using the generation of angiotensin I from endogenous angiotensinogen, during a 90-min incubation at 37 °C, in the presence of angiotensin-converting enzyme inhibitors. The generated angiotensin I was measured by RENCTK 125I competitive radioimmunoassay kit (DiaSorin) with blank subtraction of angiotensin I levels in aliquots incubated at 4 °C. From 3 February 2017, the renin activity was analyzed by liquid chromatography tandem mass spectrometry (North West London Pathology). Serum cortisol was measured by electrochemiluminescent immunoassay on a Modular Analytics E170 or Cobas 8000 e602 platform (Roche Diagnostics) at SBH and by chemiluminescent immunoassay on the ADVIA Centaur platform (Siemens) at GSTT and CUH.

As renin can be estimated as either mass or activity and the routine assay differed among centers, we took the opportunity in the largest center (SBH) to measure both mass and activity at baseline and primary outcome time points. While generally well correlated, there were some outliers (Extended Data Fig. 2). We therefore adopted a commonly used conversion factor to present all of the results as renin activity.

### Adrenal tissue collection

Adrenal tissue was collected directly from surgery. Once the adrenal was removed, this was dissected into 5 mm serial slices by the surgeon or histopathologist. All adrenal adenomas were identified and 10–200 mg of tissue was removed from each APA and adjacent normal adrenal gland (AAG) for storage in separate tubes in RNAlater solution (AM7020; Invitrogen). Slices were sent to pathology for routine clinical hematoxylin and eosin staining and IHC.

### IHC

IHC was performed on 3-μm sections cut from paraffin blocks using fully automated systems. The CYP11B1 primary antibody clone RAT-87 (MABS502; Merck) was used at a dilution of 1:100 after heat-induced epitope retrieval at pH 9.0 (BOND Epitope Retrieval Solution 2; AR9640; Leica) for 20 min. CYP11B2 primary antibody clone EPR10494 (ab168388; Abcam) was used at a dilution of 1:200 after proteolytic-induced epitope retrieval with proteinase K (BOND Enzyme Pre-Treatment Kit; AR9551; Leica) for 10 min. The primary antibody binding to tissue sections was visualized using a BOND Polymer Refine Detection system (DS9800; Leica). IHC slides were scanned using a NanoZoomer S210 (C13239; Hamamatsu) and viewed using the Hamamatsu NDP.view2 image-viewing software (U12388-01; Hamamatsu).

### Histopathological analysis

For each case, APAs, aldosterone-producing nodules (APNs), APMs and functional and non-functional hyperplasia were characterized based on the international histopathology consensus for unilateral primary aldosterone (HISTALDO)^69^. APM numbers were counted within the adrenal adjacent to the main nodule. The cell types within the main nodules (lipid rich, lipid poor or mixed) were noted. The intensity and proportion (as a percentage) of the staining of tumors with CYP11B1 and CYP11B2 were evaluated semi-quantitatively using a scoring system as follows: 1, weak; 2, intermediate; 3, strong. From these data, a *H* score was assigned using the following formula: 3 × (percentage of cells 3+) + 2 × (percentage of cells 2+) + 1 × (percentage of cells 1+). A consensus of either classical or non-classical histopathological findings was made in line with HISTALDO criteria, where: classical histopathology associated with unilateral PA included a solitary APA or APN; and non-classical histopathology included multiple APMs or multiple APNs (or multiple APMs and multiple APNs together) or aldosterone-producing diffuse hyperplasia.

### RNA and DNA extraction

RNA and DNA were extracted from tissue samples stored in RNAlater for RNA-seq, WES or Sanger sequencing. Tissue homogenization was performed in TRIzol (15596026; Life Technologies) using a FastPrep-24 5G Sample Preparation System (MP Biomedicals) as per protocol. Genomic DNA was extracted from approximately 25–30 mg of APA or adjacent normal adrenal tissue using a QIAamp DNA Mini Kit (51304; Qiagen) according to the manufacturer’s instructions. If AAG was not available to be used as a control, genomic DNA was extracted from blood using the salt extraction method. Total RNA was extracted from approximately 50–100 mg of AAG or APA tissue using the TRIzol method (15596026; Life Technologies) followed by on-column DNase I treatment (Invitorgen) and purification using Invitrogen’s PureLink RNA Mini Kit (12183018A).

### Quantitative PCR

Quantitative PCR was performed on all adenomas to assess for *CYP11B2* and *CYP11B1* messenger RNA (mRNA) expression. RNA was reverse transcribed to complementary DNA using Applied Biosystems’ High-Capacity RNA-to-cDNA Kit. TaqMan assay probes were used to quantify the gene of interest. The ΔΔCT method was used to quantify gene expression levels^70^. Supplementary Table 8 lists the commercially available TaqMan Gene Expression Assays used to quantify mRNA expression of the genes of interest.

### RNA-seq and analysis

RNA-seq was performed on functional adenomas. RNA-seq was performed by the Barts and the London Genome Centre, Blizard Institute in London. Briefly, quality control was performed using an Agilent RNA 6000 Nano reagent kit and run on an Agilent 2100 bioanalyzer (Agilent). Only samples with an RNA integrity number number of >0.8 were sequenced. Library preparation using the mRNA library preparation method was performed using a NEBNext Ultra II RNA Library Prep Kit for Illumina (New England Biolabs). Finally, sequencing was performed on Illumina’s NextSeq 500 system using the high-output 150 cycle kit version 2.5. Partek Flow (Partek) was used for RNA-seq analysis. Sequences were aligned to hg38 with STAR -2.6.1d and annotated genes were aligned to Ensembl Transcripts release 93 with Partek’s own annotation tool. Partek’s GSA tool was used to generate lists of differentially expressed genes.

### WES and analysis

Approximately 1 mg of genomic DNA was sent off for WES by the commercial company GENEWIZ. Library preparations, sequencing reactions and bioinformatics analysis were conducted at GENEWIZ as follows.

#### DNA library preparation and sequencing

Genomic DNA samples were quantified using a Qubit 2.0 Fluorometer (Thermo Fisher Scientific). Twist Human Core Exome library preparation was performed according to the manufacturer’s guidelines (Twist Bioscience).

Briefly, the genomic DNA was fragmented by acoustic shearing with a Covaris S220 instrument. Fragmented DNA was cleaned up and end repaired, as well as adenylated at the 3′ ends. Adapters were ligated to the DNA fragments and adapter-ligated DNA fragments were enriched with limited-cycle PCR. Adapter-ligated DNA fragments were validated using an Agilent TapeStation system (Agilent Technologies) and quantified using a Qubit 2.0 Fluorometer. Adapter-ligated DNA fragments were hybridized with biotinylated baits. The hybrid DNA was captured by streptavidin-coated binding beads. After extensive washing, the captured DNA was amplified and indexed with Illumina indexing primers. Post-captured DNA libraries were validated using Agilent TapeStation (Agilent) and quantified using the Qubit 2.0 Fluorometer and real-time PCR (KAPA Biosystems).

The sequencing libraries were multiplexed and clustered onto multiple lanes of a flowcell. After clustering, the flowcell was loaded onto the Illumina HiSeq instrument according to the manufacturer’s instructions. The samples were sequenced using a 2 × 150 bp paired end configuration. Image analysis and base calling were conducted using the HiSeq Control Software. Raw sequence data (.bcl files) generated from Illumina HiSeq were converted into fastq files and de-multiplexed using Illumina bcl2fastq 2.17 software. One mis-match was allowed for index sequence identification.

#### Data analysis

Sequence reads were trimmed to remove possible adapter sequences and nucleotides with poor quality using Trimmomatic version 0.38. The trimmed reads were mapped to the reference genome using the Illumina DRAGEN Bio-IT Platform. BAM files were generated as a result of this step. Somatic variants were called using the Illumina DRAGEN Bio-IT Platform in somatic mode. Paired normal samples were used in the process, if provided, or a panel of normal was used to remove technical artifacts. Variants were further filtered and any variants in the following categories were considered false positives and removed: (1) marked as common variants in dbSNP build 151; and (2) non_cancer_AC > 5 in the gnomAD exome database r2.1.1. The filtered VCF was then annotated with Ensembl Variant Effect Predictor version 95. For each variant that was mapped to the reference genome, all overlapping Ensembl transcripts were identified and the effects that each allele of the variant may have on each transcript were predicted by Variant Effect Predictor. The set of consequence terms was defined by the Sequence Ontology. The most severe impact was selected for each variant and they were used for downstream cohort analysis. The impacts of the variants were classified based on MAF document specifications. The tumor mutation load was calculated based on the number of mutations in the genome region that was targeted.

### Targeted Sanger sequencing

Complementary DNA from the APA and AAG was sequenced to confirm the presence of *KCNJ5* mutations. PCR was performed with the following forward (CAACTTGCTCGTCTTCACCA) and reverse (GAGGGTCTCCGCTCTCTTCT) primers, using AmpliTaq Gold Fast PCR Master Mix (4390939; Thermo Fisher Scientific) per the manufacturer’s instructions. We then purified 5 µl of PCR products using 2 µl of ExoSAP-IT PCR Product Cleanup Reagent (78201.1.ML; Applied Biosystems) before Sanger sequencing. Sequencing of the PCR products was performed using LIGHTRUN Tube sequencing services from Eurofins Genomics.

### Urinary steroid profiling by liquid chromatography tandem mass spectrometry

Steroids were extracted from 200 µl of a 24 h urine collection after the addition of an internal standard mixture, as described previously^71,72^. In brief, steroids were deconjugated from their sulfate and/or glucuronide conjugates through enzymatic hydrolysis with *Helix pomatia* (Sigma–Aldrich; 60 °C for 3 h). The steroids were then extracted via solid-phase extraction (C18; 100 mg; Biotage). The methanol eluent was collected and evaporated and the extract was reconstituted and run by liquid chromatography tandem mass spectrometry. The mass spectrometer was a Waters Xevo TQ-XS with an Acquity ultra-high-pressure liquid chromatography system, with an electrospray source in positive ionization mode with a methanol and water gradient system (both with 0.1% formic acid). The gradient started at 30% methanol, linearly increasing to 39% over 6 min. Next, the column was washed at 98% methanol and re-equilibrated before the next injection. Separation was achieved on a Waters HSS T3 1.2 × 50 mm 1.8 µM column. With each batch of samples, a calibration series (0–1,500 ng ml^−1^), three spiked quality control samples and seven pooled biological control urine samples were extracted. Steroids were quantified relative to their deuterated internal standard cotisol-d4 or 18-hydroxycortisol-d4, against this calibration series. In addition to previously published methods 18-hydroxycortisol (18OHF) was included in this method. Bias (percentage deviation), calculated as the accuracy at three spiked concentrations (20, 300 and 200 ng ml^−1^) was <5% for cortisol and <10% for 18OHF at all concentrations. Imprecision values (percentage relative standard deviation) measured from multiple extractions of the same urine sample (intra-assay (*n* = 7) and inter-assay (*n* = 14)) were 4.9 and 3.85% for cortisol and 5.8 and 6.21% for 18OHF, respectively.

### Reporting summary

Further information on research design is available in the Nature Portfolio Reporting Summary linked to this article.

## Online content

Any methods, additional references, Nature Portfolio reporting summaries, source data, extended data, supplementary information, acknowledgements, peer review information; details of author contributions and competing interests; and statements of data and code availability are available at 10.1038/s41591-022-02114-5.

## Supplementary information

Supplementary Information Supplementary Tables 1–8.

Reporting Summary

Supplementary Data Study protocol and statistical analysis plan.

## Figures and Tables

**Extended Data Table 1:**
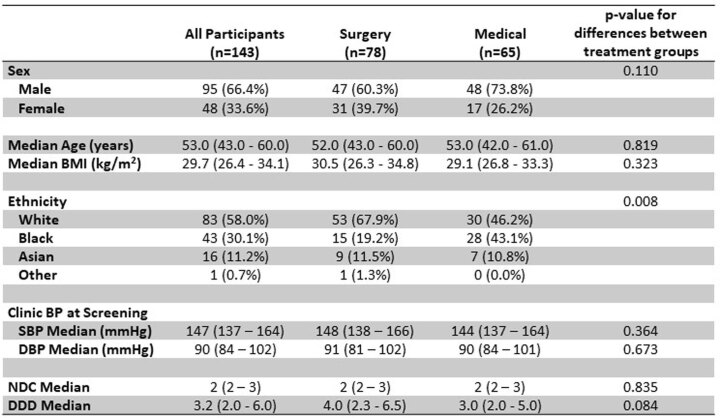
Demographics and baseline characteristics (all patients)

Demographics and baseline characteristics of all patients recruited into the study (excluding 3 patients who withdrew before having both or either investigation). Data for patients managed with surgery or medical therapy is also shown. Numbers in brackets indicate percentage, range in brackets indicate first and third quartiles. For categorical data the Fisher Exact test was used. For continuous data the Mann-Whitney-Wilcoxon test was used. BMI, body mass index; BP, blood pressure; SBP, systolic BP; DBP, diastolic BP; NDC, number of different classes of antihypertensive medications; DDD, defined daily dose of antihypertensive medications.

**Extended Data Table 2:**
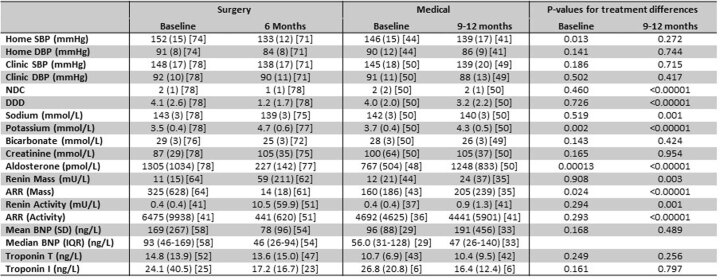
Clinical and biochemical parameters

Clinical and biochemical parameters used in the calculation of primary and secondary outcomes. Data for patients managed with surgery or medical therapy is also shown. Unless otherwise stated numbers show mean; round brackets indicate standard deviation; square brackets indicate n (number of patients). For categorical data the Fisher Exact test was used. For continuous data the Mann-Whitney-Wilcoxon test was used. Reference ranges: Sodium 133-146 mmol/L, Potassium 3.5-5.3 mmol/L, Bicarbonate 22-29 mmol/L, Creatinine 45-105 mmol/L, Aldosterone 150-550 pmol/L, Renin mass 5.4-30 mU/L, Renin activity 0.5-3.0 nmol/L/hr, ARR (mass) <91 pmol/mU, ARR (activity) <1000 (pmol/L)/(nmol/L/hr), BNP < 400 ng/L, Troponin T < 14 ng/L (St Bartholomew’s and Guy’s and St Thomas’ Hospital), Troponin I < 58.1 ng/L (Cambridge University Hospital). SBP, systolic BP; DBP, diastolic BP; ARR, aldosterone renin ratio; BNP, N-terminal pro-B-type natriuretic peptide; NDC, number of different classes of antihypertensive medications; DDD, defined daily dose; SD, standard deviation; IQR, interquartile range.

## Data Availability

Clinical trial data may be granted to qualified academic researchers in the field upon approval by the study management committee and subject to appropriate data sharing and transfer agreements. Requests for data should include rationale and the relevance of the proposed research, hypothesis, research methodology, statistical analysis plan and publication plan. Genotyping and transcriptome data are available from the Sequence Read Archive under accession code PRJNA894093.
