# Supplementary material for: [11C]metomidate PET-CT versus adrenal vein sampling for diagnosing surgically curable primary aldosteronism: a prospective, within-patient trial
Source: Nat Med. 2023 Jan 16;29(1):190–202. doi: 10.1038/s41591-022-02114-5 (PMC9873572; doi:10.1038/s41591-022-02114-5)
Supplement: Supplementary file 1 — Supplementary Tables 1–8. [file 41591_2022_2114_MOESM1_ESM.pdf]

nature medicine

Article

https://doi.org/10.1038/s41591-022-02114-5

[^{11}C]metomidate PET-CT versus adrenal vein sampling for diagnosing surgically curable primary aldosteronism: a prospective, within-patient trial

In the format provided by the authors and unedited

Supplementary Table 1. Definition of success of PA/hypertension as per PASO international consensus[14]

Outcome measure	Definition
Complete clinical success	Complete clinical success will be declared when both **(a) and (b)** have occurred:<br>(a) Normalisation of BP, defined as SBP<135 and DBP<85 mmHg on home BP; if home BP not available, then defined as SBP<140 and DBP<90 mmHg on clinic BP<br>(b) Not taking any antihypertensive medications
Partial clinical success	Partial clinical success will be declared when the criteria for complete success are not met, and **either (a) or (b)** has occurred:<br>(a) reduction in BP, with no increase in antihypertensive medication, i.e. both of (i) and (ii) have occurred:<br>(i) BP has reduced, or has normalised, since baseline (based on home BP if available, otherwise based on clinic BP); reduction in BP defined as<br>(1) a reduction in SBP of ≥20mmHg, or<br>(2) a reduction in DBP of ≥10mmHg, with a change in SBP of <20mmHg<br>(ii) the DDD of antihypertensive medication being used is <150% of baseline<br>(b) reduction in antihypertensive medication, with no increase in BP, i.e.<br>(i) BP is unchanged since baseline (based on home BP if available, otherwise based on clinic BP); unchanged BP defined as<br>(1) a change in SBP of <20mmHg, and<br>(2) a change in DBP of <10mmHg<br>(ii) the DDD of antihypertensive medication being used is ≤50% of baseline
Absent clinical success	An absence of clinical success will be declared when neither partial nor complete clinical success can be declared.
Complete biochemical success	Complete biochemical success will be declared when both of the following occur:<br>• Serum potassium ≥3.6 mmol/L and<br>• Normalisation of ARR ($ARR_{activity}$ <750 pmol/L per ng/ml/hr, if renin activity is recorded, otherwise ARR_{mass} <91 miU/L)
Partial biochemical success	Partial biochemical success will be declared when the criteria for complete success are not met, and both of the following occur:<br>• Serum potassium ≥3.6 mmol/L and<br>• ≥50% reduction in serum aldosterone concentration
Absent biochemical success	An absence of biochemical success will be declared when neither partial nor complete clinical success can be declared.

Defined Daily Dose (DDD) is the assumed average maintenance dose per day for a drug used for its main indication in adults (DDD; ATC/DDD Index 2010: http://www.whocc.no/atc_ddd_index/ 2010 [date accessed 16/12/2021])
PA, primary aldosteronism; BP, blood pressure; SBP systolic BP; DBP, diastolic BP.

Supplementary Table 2

Sensitivity Analysis Results

	95% Bootstrap Interval	Impact of AVS Failures
Clinical Success		
Complete	(-0.235, 0.073)	Not Significant
Partial or Complete	(-0.086, 0.222)	Not Significant
Biochemical Success		
Complete	(-0.018, 0.271)	Not Significant
Partial or Complete	(0.009, 0.291)	Significant (Excluding AVS failures favours AVS)
Modified Clinical Success		
Complete	(-0.226, 0.081)	Not Significant
Partial or Complete	(-0.077, 0.231)	Not Significant

Supplementary Table 2: Sensitivity analysis

Supplementary analysis using Bootstrap method showing effect of excluding AVS ‘failures’ on the relative accuracy of MTO and AVS in predicting biochemical but not clinical success.

Supplementary Table 3. ‘Modified’ definition of success of PA/hypertension

Outcome measure	Definition
Modified complete clinical success	Complete modified clinical success will be declared when **both (a) and (b)** have occurred:<br>(a) Normalisation of SBP, defined as SBP<135mmHg on home BP; if home BP not available, then defined as SBP<140mmHg on clinic BP<br>(b) Not taking any antihypertensive medications
Modified partial clinical success	Partial modified clinical success will be declared when the criteria for complete success are not met, and **either (a) or (b)** has occurred:<br>(a) reduction in SBP, with no increase in antihypertensive medication, i.e. both of (i) and (ii) have occurred:<br>(i) SBP has reduced by ≥20mmHg, or has normalised, since baseline (based on home BP if available, otherwise based on clinic BP)<br>(ii) the number of classes of antihypertensive medication being used is no more than 1 more than at baseline<br>(b) reduction in antihypertensive medication, with no increase in SBP, i.e.<br>(i) SBP is unchanged since baseline (based on home BP if available, otherwise based on clinic BP); unchanged SBP defined as a change in either direction of <20mmHg<br>(ii) the number of classes of antihypertensive medication being used has reduced by 2 or more since baseline, or the use of antihypertensive medication has been stopped
Modified absent clinical success	An absence of clinical success will be declared when neither partial nor complete clinical success can be declared.

Supplementary Table 3. Definition of success of PA/Hypertension

a. Definition of success of PA / Hypertension as defined by the international PASO consensus.[14]
b. ‘Modified’ definition of clinical success.

Defined Daily Dose (DDD) is the assumed average maintenance dose per day for a drug used for its main indication in adults (DDD; ATC/DDD Index 2010: http://www.whocc.no/atc_ddd_index/ 2010 [date accessed 16/12/2021])

PA, primary aldosteronism; BP, blood pressure; SBP systolic BP; DBP, diastolic BP.

Supplementary Table 4

Adrenal no.	Gene 1	Mutation 1	Gene 2	Mutation 2	Method of detection
1	*CACNA1D*	p.F767L			RNA seq
2	*CACNA1D*	p.V1373M			RNA seq
3	*CACNA1D*	p.G403R			WES & RNA seq
4	*CACNA1D*	p.I1035T			RNA seq
5	*CACNA1D*	p.R990G			RNA seq
6	*CACNA1D*	p.I750M			WES & RNA seq
7	*CACNA1D*	p.F767V			RNA seq
8	*CACNA1D*	p.F767V			WES & RNA seq
9	*CACNA1D*	p.V621G			WES & RNA seq
10	*CACNA1D*	p.G403R			RNA seq
11	*CACNA1D*	p.R639P			WES & RNA seq
12	*CACNA1D*	p.F747L			WES & RNA seq
13	*CACNA1D*	p.F747L			WES & RNA seq
14	*CACNA1D*	p.I1152N			RNA seq
15	*CACNA1D*	p.V1373M			RNA seq
16	*CACNA1D*	p.R990H			RNA seq
17	*CACNA1D*	p.V1373M			WES
18	*CACNA1D*	p.R990G			RNA seq
19 nodule 1	*CACNA1D*	p.F747L			RNA seq
19 nodule 2	*CACNA1D*	p.F747L			RNA seq
20	*CACNA1D*	p.G403R			WES & RNA seq
21	*KCNJ5*	p.L168R			Sanger seq
22	*KCNJ5*	p.L168R			WES
23	*KCNJ5*	p.E145Q			WES & RNA seq
24	*KCNJ5*	p.G151R			WES & RNA seq
25	*KCNJ5*	p.G151R			RNA seq
26	*KCNJ5*	p.L168R			WES & RNA seq
27	*KCNJ5*	p.G151R			RNA seq
28	*KCNJ5*	p.G151R			WES & RNA seq
29	*KCNJ5*	p.G151R			WES & RNA seq
30	*KCNJ5*	p.L168R			RNA seq
31	*KCNJ5*	p.L168R			WES
32	*KCNJ5*	p.G151R			RNA seq
33	*KCNJ5*	p.G151R			WES & RNA seq
34	*KCNJ5*	p.L168R			Sanger seq
35	*KCNJ5*	p.G151R			WES
36	*KCNJ5*	p.G151R			RNA seq
37	*KCNJ5*	p.G151R			WES
38	*KCNJ5*	p.G151R			WES & RNA seq
39	*ATP1A1*	p.E960_L964delinsV			RNA seq
40	*ATP1A1*	p.L104R			RNA seq
41	*ATP1A1*	p.L104R			RNA seq
42	*ATP1A1*	p.L104R			WES & RNA seq
43	*ATP1A1*	p.L104R			WES
44 nodule 1	*ATP1A1*	p.L104R			RNA seq
44 nodule 2	*ATP1A1*	p.L104R			RNA seq
45	*ATP1A1*	p.L104R			WES & RNA seq
46	*ATP1A1*	p.E960_L964delinsV			WES & RNA seq
47	*ATP1A1*	p.E960_L964delinsV			WES & RNAseq
48	*ATP1A1*	p.G99-L104del			WES
49	*ATP1A1*	p.L104R			WES
50	*ATP1A1*	p.F959_962del			RNA seq
51	*ATP2B3*	p.L425_V426del			WES & RNA seq
52	*ATP2B3*	p.L425_V426del			WES & RNA seq
53	*ATP2B3*	p.G123R*			WES & RNA seq
54	*ATP2B3*	p.L425_V426del	*CTNNB1*	p.S31P	RNA seq
55	*ATP2B3*	p.L425_V426del			RNA seq
56	*ATP2B3*	p.L425_V426del			WES
57	*ATP2B3*	p.L425_V426del			WES & RNA seq
58	*GNAQ*	p.Q209L	*CTNNB1*	p.G34R	RNA seq
59	*GNAQ*	p.Q209H	*CTNNB1*	p.G34E	RNA seq
60	*CLCN2*	p.N356_L359del**			WES & RNA seq

Supplementary Table 4: Genotyping APAs

Table showing mutations identified in APAs, in genes previously associated with somatic mutations in APAs. The method of detection (RNA seq, WES, Sanger sequencing) is also shown.

APA, aldosterone producing adenoma; RNA seq, RNA sequencing; WES, whole exome sequencing; Sanger seq, sanger sequencing.

Supplementary Table 5

a.

		Proportional Odds Model	
		**OR (95% CI)**	**p**
**Genotype**	***KCNJ5* vs. Other**	3.43 (0.58, 20.74)	p=0.087
	***CACNA1D* vs. Other**	0.44 (0.10, 1.74)	
	KCNJ5* vs. *CACNA1D	7.84 (1.24, 55.96)	
**Age**	**per 10 years**	0.48 (0.24, 0.87)	p=0.015
**Sex**	**Female vs. Male**	3.44 (0.70, 18.29)	p=0.128
**Ethnicity**	**White vs. Black**	3.36 (0.70, 17.39)	p=0.304
	**Asian/Other vs. Black**	2.08 (0.31, 15.55)	
	**White vs. Asian/Other**	1.61 (0.33, 7.63)	

b.

		Proportional Odds Model	
		**OR (95% CI)**	**p**
**Genotype**	***KCNJ5* vs. Other**	4.08 (0.64, 28.74)	p=0.074
	***CACNA1D* vs. Other**	0.47 (0.11, 1.89)	
	KCNJ5* vs. *CACNA1D	8.77 (1.34, 66.50)	
**Age**	**per 10 years**	0.51 (0.25, 1.00)	p=0.049
**Sex**	**Female vs. Male**	3.02 (0.57, 16.81)	p=0.193
**Ethnicity**	**White vs. Black**	3.12 (0.64, 16.81)	p=0.366
	**Asian/Other vs. Black**	2.07 (0.30, 15.42)	
	**White vs. Asian/Other**	1.51 (0.30, 7.24)	

Supplementary Table 5: Proportional Odds Model

Results from logistic regression analysis of variables predicting clinical success
a. All genotyped patients.
b. Excluding 2 patients with double *GNAQ&CTNNB1* mutations.
The p-values are from proportional odds logistical regressions and are based on the resulting t values for the corresponding explanatory variable in the model.

Supplementary Table 6

	No. of participants	
	n=128	n=143
SI Right AV ≥3	104 (81%)	115 (80%)
SI Right AV <3	24 (19%)	28 (20%)
SI Left AV ≥3	117 (91%)	132 (92%)
SI Left AV <3	11 (9%)	11 (8%)
SI Both AV ≥3	98 (77%)	109 (76%)
LI ≥4	56 (57%)	63 (58%)
LI 3-4	8 (8%)	12 (7%)
LI <3	33 (34%)	37 (34%)

Supplementary Table 6. Selectivity and Lateralisation Index of all participants

Table showing selectivity index (SI) of right, left or both adrenal veins (AV). SI is calculated by $cortisol_{AV}$ / $cortisol_{IVC}$. SI ≥ 3 suggests successful cannulation of the adrenal vein. Lateralization Index (LI) is only calculated if SI in both AV is ≥3. It is calculated by higher A/C (aldosterone/cortisol) ratio in one adrenal divided by A/C ratio in contralateral adrenal. LI ≥4 suggests unilateral PA, LI <3 suggests bilateral PA. Results are shown for all patients in the full analysis set (n=143), and in the 128 patients who completed their allocated treatment with 6-9 months follow up data.

IVC, inferior vena cava; AV adrenal vein

Supplementary Table 7

Diagnostic Tests		Normalisation of renin and ARR after surgery (ie confirmed unilateral PA)	
AVS	PET-CT	No	Yes
Unsuccessful	Bilateral	A (=9%)	B (=0%)
Unsuccessful	Unilateral	C (=1%)	D (=10%)
Bilateral	Bilateral	E (=35%)	F (=0%)
Bilateral	Unilateral	G (=1%)	H (=9%)
Unilateral	Bilateral	I (=0.5%)	J (=3.5%)
Unilateral (c)	Unilateral	K (=1%)	L (=29%)
Unilateral (d)	Unilateral	M (=0.25%)	O (=0.25%)
Unilateral	Unilateral (d)	P (=0.25%)	Q (=0.25%)

Supplementary Table 7. Predicted frequencies and permutations of outcomes from both investigations

Table showing potential outcomes from the two separate investigations and the predicted frequency of each outcome based on experience at each participating center. The power calculations were based on the above predicted frequencies.
c = concordant (MTO and AVS lateralizes to same side); d = discordant (MTO and AVS lateralizes to opposite sides)
ARR, aldosterone renin ratio

Supplementary Table 8

Gene	Assay ID	Amplicon Length	Properties
18S	Hs99999901_s1	187	Primer and probe within same exon
CYP11B2	Hs01597732_m1	137	Spans exon 5 - 6
CYP11B1	Hs01596404_m1	137	Spans exon 2 - 3

Supplementary Table 8. Quantitative PCR probes

Features and assay IDs of Taqman Gene Expression Assays used in qPCR quantification of mRNA expression of genes of interest.
