# Supplementary material for: [11C]metomidate PET-CT versus adrenal vein sampling for diagnosing surgically curable primary aldosteronism: a prospective, within-patient trial
Source: Nat Med. 2023 Jan 16;29(1):190–202. doi: 10.1038/s41591-022-02114-5 (PMC9873572; doi:10.1038/s41591-022-02114-5)
Supplement: Supplementary file 2 — Reporting Summary [file 41591_2022_2114_MOESM2_ESM.pdf]

Corresponding author(s): Morris Brown
Last updated by author(s): 2022.10.24

Reporting Summary

Nature Research wishes to improve the reproducibility of the work that we publish. This form provides structure for consistency and transparency in reporting. For further information on Nature Research policies, see our Editorial Policies and the Editorial Policy Checklist.

Statistics

For all statistical analyses, confirm that the following items are present in the figure legend, table legend, main text, or Methods section.

n/a	Confirmed	
☐	☒	The exact sample size (*n*) for each experimental group/condition, given as a discrete number and unit of measurement
☐	☒	A statement on whether measurements were taken from distinct samples or whether the same sample was measured repeatedly
☐	☒	The statistical test(s) used AND whether they are one- or two-sided<br>*Only common tests should be described solely by name; describe more complex techniques in the Methods section.*
☐	☒	A description of all covariates tested
☐	☒	A description of any assumptions or corrections, such as tests of normality and adjustment for multiple comparisons
☐	☒	A full description of the statistical parameters including central tendency (e.g. means) or other basic estimates (e.g. regression coefficient) AND variation (e.g. standard deviation) or associated estimates of uncertainty (e.g. confidence intervals)
☐	☒	For null hypothesis testing, the test statistic (e.g. *F*, *t*, *r*) with confidence intervals, effect sizes, degrees of freedom and *P* value noted<br>*Give P values as exact values whenever suitable.*
☒	☐	For Bayesian analysis, information on the choice of priors and Markov chain Monte Carlo settings
☒	☐	For hierarchical and complex designs, identification of the appropriate level for tests and full reporting of outcomes
☒	☐	Estimates of effect sizes (e.g. Cohen's *d*, Pearson's *r*), indicating how they were calculated

Our web collection on statistics for biologists contains articles on many of the points above.

Software and code

Policy information about availability of computer code

Data collection

Clinical data was collected and entered into the case report form (CRF) by members of the study team. The CRF was managed by the Robertson Centre for Bioinformatics.

NextSeq 500 high-output 150 cycle kit v2.5 and bcl2fastq Conversion Software v1.8.4: RNA sequencing

CFX Manage TM Software v3.1 (Bio-Rad, USA) or 7900 SDS v2.4.1 (Applied Biosystems, USA): qPCR data acquisition

Image analysis and base calling were conducted by the HiSeq Control Software (HCS). Raw sequence data (.bcl files) generated from Illumina HiSeq was converted into fastq files and de-multiplexed using Illumina bcl2fastq 2.17 software: Whole exome sequencing

IHC slides were scanned using Nanozoomer S210 (C13239, Hamamatsu)

PET CT acquisition using GE Xeleris (version 4.0) software on Xeleris functional imaging workstation

Urine steroid profile data was collected using a Waters Xevo-XS and an acquity uPLC using the masslynx software, version 4.2

Data analysis

Clinical data was analysed using the 64 bit version 4.1.0 of R. The following R packages were also used.
R Package Version
brant 0.3.0
car 3.0.11
DescTools 0.99.42
devEMF 4.0.2
dplyr 1.0.7
eq5d 0.9.0

flextable 0.6.6
ggmosaic 0.3.3
ggplot2 3.3.5
Hmisc 4.5.0
lme4 1.1.27.1
MASS 7.3.54
metafor 3.0.2

mlogit 1.1.1
officer 0.3.18
pbkrtest 0.5.1
pROC 1.17.0.1
RODBC 1.3.17
rtf 0.4.14.1
stringr 1.4.0
tidyverse 1.3.1
vioplot 0.3.6

Graphpad Prism software v.9 for statistical analysis of experimental data

Partek Flow software (Partek, St. Louis, Missouri, United States), including its annotation tool and GSA tool was used for RNA sequencing analysis.

For Whole Exome Sequencing: Sequence reads were trimmed to remove possible adapter sequences and nucleotides with poor quality using Trimmomatic v.0.38. The trimmed reads were mapped to the reference genome using the Illumina Dragen Bio-IT Platform. Somatic variants were called using the Illumina Dragen Bio-IT Platform in somatic mode

IHC slides were viewed using NDP.view2 Image viewing software (U12388-01, Hamamatsu)

Targetlynx software was used for urine steroid profile quantification

For manuscripts utilizing custom algorithms or software that are central to the research but not yet described in published literature, software must be made available to editors and reviewers. We strongly encourage code deposition in a community repository (e.g. GitHub). See the Nature Research guidelines for submitting code & software for further information.

Data

Policy information about availability of data

All manuscripts must include a data availability statement. This statement should provide the following information, where applicable:

- Accession codes, unique identifiers, or web links for publicly available datasets
- A list of figures that have associated raw data
- A description of any restrictions on data availability

Clinical trial data may be granted to qualified academic researchers in the field upon approval by the study management committee and subject to appropriate data sharing and transfer agreements. Requests for data should include rationale and relevance of proposed research, hypothesis, research methodology, statistical analysis plan and publication plan. Genotyping and transcriptome data is available from Sequence Read Archive under accession no. PRJNA894093.

Field-specific reporting

Please select the one below that is the best fit for your research. If you are not sure, read the appropriate sections before making your selection.

☒ Life sciences ☐ Behavioural & social sciences ☐ Ecological, evolutionary & environmental sciences

For a reference copy of the document with all sections, see nature.com/documents/nr-reporting-summary-flat.pdf

Life sciences study design

All studies must disclose on these points even when the disclosure is negative.

Sample size

The calculations on sample size are based on our stated intention that AVS will only be performed in centres whose robust audit data indicate a bilateral cannulation rate of ≥75%, as the testing of the hypothesis will have maximum clinical value if 11C metomidate scanning is compared with current best practice AVS. It also assumes that 100% of metomidate scans will provide a technically satisfactory result (we consider this to be reasonable, based on the Cambridge experience since the investigation was first introduced in May 2009).

The design of the study (and therefore the statistical analysis) presupposes that there will be a group of patients for whom the results from metomidate scanning and AVS will differ, but that the former is correct more often and that this difference is statistically significant. Given the results of the proof-of-concept study, it is inevitably the case that there will be a large group of patients for whom the two tests are concordant. The different scenarios of concordant/discordant, unilateral/bilateral, together with their estimated frequencies, are shown in Supplementary Table 7, and it is these figures that have been used the model for the sample size calculation and proposed analysis.

Recruitment of 165 patients across 3 centres over 3 years (<1/centre/month), with an estimated 70 proceeding to adrenalectomy, permits 90% power at alpha=0.01 of detecting non inferiority of PET CT in relation to AVS within a margin 16.7%.

Data exclusions	Data from the second MTO PET-CT (while on spironolactone) from one participant in the spironolactone sub-study was excluded from the sub-study analysis. Decision for this exclusion was made on the basis this participant failed to take dexamethasone prior to his second MTO PET-CT scan. Failure to do so rendered his scan uninterpretable and would not have made a fair comparison with the original PET-CT. Due to the small number of participants in the sub-study a decision was made by the MDT to include one further participant in the sub-study, to replace this participant.
Replication	Home BP measurements were average readings over four consecutive days. Each day 3 readings taken twice daily were recorded (24 in total). Office BP measurements were average recordings of 3 sequential readings.<br>Quantitative PCR experiments were conducted with a minimum of technical duplicates.<br>All experimental data used biological patient samples where technical replications were successful. Biological replication is not application in this setting.
Randomization	The order that each participant underwent AVS or MTO PET-CT was determined by a minimization program within the study data center (Robertson Centre for Biostatistics, University of Glasgow), designed to maintain balance with respect to study site, gender, and age (<55 or ≥55 years).
Blinding	All participants underwent both AVS and MTO PET-CT. MTO PET-CT was considered the 'experimental' investigation of the two, therefore results from MTO were always reviewed first at MDT, blinded to the results of AVS, such that grading of MTO and decisions regarding recommendation for surgery based on MTO alone were not biased by results from AVS.

Reporting for specific materials, systems and methods

We require information from authors about some types of materials, experimental systems and methods used in many studies. Here, indicate whether each material, system or method listed is relevant to your study. If you are not sure if a list item applies to your research, read the appropriate section before selecting a response.

Materials & experimental systems

n/a	Involved in the study
☐	☒ Antibodies
☒	☐ Eukaryotic cell lines
☒	☐ Palaeontology and archaeology
☒	☐ Animals and other organisms
☐	☒ Human research participants
☐	☒ Clinical data
☒	☐ Dual use research of concern

Methods

n/a	Involved in the study
☒	☐ ChIP-seq
☒	☐ Flow cytometry
☒	☐ MRI-based neuroimaging

Antibodies

Antibodies used	Primary antibodies for IHC<br>CYP11B1 primary antibody clone RAT-87 (MABS502, Merck) dilution of 1:100<br>CYP11B2 primary antibody clone EPR10494 (ab168388, Abcam) was used at a dilution of 1:200
Validation	IHC staining is used routinely in the clinical setting at CUH for diagnosis of aldosterone and cortisol secreting adenomas. Both antibodies were validated in accordance with in house laboratory protocols for use on human tissue sections, using appropriate positive and negative controls.

Human research participants

Policy information about studies involving human research participants

Population characteristics	Population characteristics are shown in Table 1 and Supplementary Table 2. No differences in age, gender, clinic BP or antihypertensive pill burden at screening were observed between the medical or surgical groups.
Recruitment	Participants were recruited from tertiary endocrinology or complex hypertension clinics from three different hospitals in London. Referral to these our recruiting centers primarily came from hospitals in the south east, south west and east of England and the Midlands, with a few participants from further afield (North of England). Participation in the study was offered to all eligible participants seen at the three recruiting sites.<br><br>Potential selection bias include willingness for surgery as those who preferred medical management may not have been referred for lateralization investigations.
Ethics oversight	The study protocol was approved by the National Research Ethics Service (Dulwich Research Ethics Committee, IRAS project ID 189508) and the Health Research Authority and Administration of Radioactive Substances Advisory Committee, and registered on ISRCTN (Identifier 91387205, https://doi.org/10.1186/ISRCTN91387205) and on ClinicalTrials.gov (Identifier: NCT02945904). In addition the study protocol was approved by the local Research and Development department at each participating center.

Note that full information on the approval of the study protocol must also be provided in the manuscript.

Clinical data

Policy information about clinical studies

All manuscripts should comply with the ICMJE guidelines for publication of clinical research and a completed CONSORT checklist must be included with all submissions.

Clinical trial registration	ClinicalTrials.gov Identifier: NCT02945904
Study protocol	Full study protocol is included in the submission to Nature Medicine and will be made available on publication
Data collection	Participants were recruited from Endocrinology or Hypertension clinics at the three study sites in the United Kingdom: St Bartholomew's Hospital, Queen Mary University of London (SBH); Addenbrooke's Hospital, University of Cambridge (CUH) and Guy's and St Thomas' Hospital (GSTT). Participants were enrolled between 2nd December 2016 and 11th September 2020. Data collection occurred at each study visit, as per study protocol. The final study visit was conducted on 19th February 2021 with datalock on 26th February 2021.
Outcomes	The primary outcome measures were accuracy of MTO PET-CT and AVS in predicting unilateral PA, by measuring normalisation of aldosterone, renin and potassium, and change in blood pressure, following adrenalectomy. Assessment of success were graded as partial/complete biochemical/clinical cure as defined by the PASO consensus.<br><br>Secondary outcome measures investigated predictors of cure post adrenalectomy, including change in aldosterone, renin, ARR, potassium, troponin, BNP, home / office BP, number of antihypertensive medications or cardiac MRI measurements between baseline and 6 months post-surgery. Influence of BP response to spironolactone, MTO PET-CT and AVS measurements and genotype of APAs on likelihood of cure were also explored.
