# Supplementary material for: [11C]metomidate PET-CT versus adrenal vein sampling for diagnosing surgically curable primary aldosteronism: a prospective, within-patient trial
Source: Nat Med. 2023 Jan 16;29(1):190–202. doi: 10.1038/s41591-022-02114-5 (PMC9873572; doi:10.1038/s41591-022-02114-5)
Supplement: Supplementary file 3 — Study protocol and statistical analysis plan. [file 41591_2022_2114_MOESM3_ESM.pdf]

--

CLINICAL STUDY PROTOCOL

IS Metomidate PET-CT superior to Adrenal venous sampling in predicting outcome from adrenalectomy in patients with primary Hyperaldosteronism (MATCH)

Protocol Version: 5.0
02/04/2020

2.0 STUDY TEAM **5**

3.0 PROTOCOL SYNOPSIS **8**

SHORT TITLE OF CLINICAL TRIAL 8
SPONSOR NAME 8
RESEARCH IDENTIFICATION NUMBER 8
MEDICAL CONDITION OR DISEASE UNDER INVESTIGATION 8
PURPOSE OF CLINICAL TRIAL 9
PRIMARY 9
OBJECTIVE /AIM 9
SECONDARY OBJECTIVE/AIMS (S) 10
STUDY DESIGN 11
STUDY 14
OUTCOME MEASURES 14
SAMPLE SIZE 16
STUDY SITES 16
SUMMARY OF ELIGIBILITY CRITERIA 17
PROCEDURES: 20
SCREENING & ENROLMENT 20
11-CMETOMIDATE PET CT SCAN/ADRENAL VEIN SAMPLING 21
BASELINE VISIT 23
STUDY VISITS (SEE ALSO 2.3: SCHEDULE OF ASSESSMENTS AND PROCEDURES) 24
TREATMENT AT STUDY END 24
PROCEDURES FOR SAFETY MONITORING DURING TRIAL 24
CRITERIA FOR WITHDRAWAL OF PATIENTS ON SAFETY GROUNDS 24
ADVERSE EVENTS AND SERIOUS ADVERSE EVENTS 25
DIRECT ACCESS TO SOURCE DATA / DOCUMENTS 25
ETHICAL CONSIDERATIONS 25
INDEMNITY 26
EMERGENCY CONTACTS 26
3.1 SCHEDULE OF ASSESSMENTS AND PROCEDURES 26

4.0 RATIONALE **30**

4.1 MEDICAL BACKGROUND 30
4.2 HYPOTHESIS AND NOVEL ASPECTS OF THE TRIAL 32
4.3 RISK-BENEFIT CONSIDERATIONS 34

5.0 OBJECTIVES **35**

5.1 ASSESSMENT OF EFFICACY AND SAFETY 35
5.2 PRIMARY OBJECTIVES/AIMS 35
5.3 SECONDARY OBJECTIVES AND AIMS 35
5.4 PRIMARY OUTCOME MEASURES 36
5.5 SECONDARY OUTCOME MEASURES 36

6.0 TRIAL DESIGN **36**

6.1 11C- METOMIDATE PET CT 37
6.2 (18F) CETO 37
6.2 ADRENAL VEIN SAMPLING 38

7.0 CARDIAC MAGNETIC RESONANCE IMAGING SCAN (CMR) **39**

8.0 SUB-STUDY OF REPEAT METOMIDATE PET CT BEFORE AND AFTER SPIRONOLACTONE/EPLERENONE THERAPY **39**

9.0 FOLLOW-UP MANAGEMENT AND INVESTIGATIONS.............................39
9.1 Stopping Criteria...39
9.2 Study population 40
10.0 INCLUSION/EXCLUSION CRITERIA: 40
10.1 Inclusion Criteria..40
10.2 Exclusion Criteria...41
10.3 Withdrawal Criteria...41
11.0 DIAGNOSTIC INVESTIGATIONS 41
12.0 REQUIREMENTS FOR PARTICIPATING SITES AND INVESTIGATORS.43
12.1 Trial Sites and Numbers of Participants 43
12.2 Expected duration of the clinical research...43
13.0 PREMATURE SITE CLOSURE...43
13.1 Premature termination of the clinical research 43
14.0 SELECTION OF THE PARTICIPANTS..44
14.1 Screening for eligibility 44
14.2 Informed Consent 44
14.3 Withdrawal of Informed Consent..44
15.0 ENROLMENT...45
15.1 Violation of Eligibility criteria 45
16.0 TRIAL PROCEDURES...45
16.1 Screening and enrolment visit..45
16.2 Baseline Visit 46
16.3 Spironolactone/Eplerenone Therapy visit (Baseline Visit) 47
16.4 Post-surgical and non-surgical follow up schedule visits at 1, 3,6,12 and 24 months...47
16.5 Unscheduled Visits..48
16.6 Premature termination of therapy or follow up and replacement of withdrawals. 48
16.7 Plan of Further Treatment 49
17.0 ADVERSE EVENTS (AE/SAE)..49
17.1 Reporting to research ethics committee..49
17.2 Annual progress report 50
18.0 LABORATORY ASPECT OF THE RESEARCH 50
19.0 STATISTICAL ASPECT OF THE RESEARCH..................................51
20.0 STUDY OUTCOMES 51
20.1 Primary Outcome(s)..51
20.2 Secondary Outcomes...51
21.0 PLANNED METHODS OF ANALYSIS 52
22.0 SAFETY ANALYSIS. 53
22.1 Interim Analysis..53
22.2 Final Analysis...53

23.0 SAMPLE CALCULATION 53
24.0 ETHICAL, LEGAL AND ADMINISTRATIVE ASPECTS 54
24.1 ETHICS COMMITTEE 54
24.2 DATA MONITORING AND ETHICS COMMITTEE (DMEC): 54
24.3 CRITICAL ENDPOINTS COMMITTEE 54
24.4 THE STEERING COMMITTEE 54
25.0 DATA HANDLING 55
25.1 DATA CAPTURE 55
25.2 DATA COLLECTION AND ECRF 55
25.3 DATA MANAGEMENT. 55
25.4 RECORD RETENTION 56
25.5 RECORD ARCHIVAL 56
26.0 QUALITY CONTROL AND ASSURANCE 56
26.1 DIRECT ACCESS TO SOURCE DATA AND DOCUMENTS 56
26.2 MONITORING 56
26.3 AUDITS 57
26.4 INSPECTIONS 57
27.0 DATA PROTECTION 57
27.1 ADMINISTRATIVE AGREEMENTS AND PROTOCOL ADHERENCE 57
27.2 PROTOCOL AMENDMENTS 58
28.0 FUNDING 58
28.1 INSURANCE 58
28.2 PUBLICATION POLICY 58
28.3 CASE PAYMENTS 58
29.0 CONFIRMATION OF THE FINAL PROTOCOL 59
29.1 PROTOCOL AGREEMENT 60
30.0 APPENDICES 61
31.0 PHARMACOGENETIC RESEARCH 61
32.0 STUDY POPULATION AND ASSESSMENT 61
33.0 CALCULATION OF PROPOSED SAMPLE SIZE 62
34.0 REFERENCES 64

2.0 Study Team

**Sponsor**	Queen Mary University of London (QMUL)
**Chief Investigator**	Professor M J Brown<br>Professor of Endocrine Hypertension<br>The Barts Heart Centre,<br>William Harvey Research Institute,<br>Queen Mary University of London,<br>Charterhouse Square,<br>London EC1M 6BQ<br><br>Email: morris.brown@qmul.ac.uk<br>Telephone: 020 7882 3901 Mobile 07710 447223
**Principle Investigators**	Dr Mark Gurnell<br>Senior Lecturer in Endocrinology<br>University of Cambridge<br>Addenbrookes Hospital,<br>Cambridge<br>CB2 2QQ
	Professor William Drake<br>Prof of Clinical Endocrinology<br>Barts and the London School of Medicine and Dentistry<br>W Smithfield<br>London - City of London<br>EC1A 7BE<br>02034657264
**Collaborators**	**Prof Franklin Aigbirhio,** Director of Cyclotron Unit (WBIC), Cambridge. Established 11C-metomidate synthesis and (18F) CETO University of Cambridge<br>Addenbrookes Hospital,<br>Cambridge<br>CB2 2QQ
	**Dr H Cheow,** Consultant in Nuclear Medicine and PET, Cambridge University Hospital Foundation Trust (CUHFT)<br>University of Cambridge<br>Addenbrookes Hospital,<br>Cambridge<br>CB2 2QQ
	**Dr Alison Marker,** Lead Consultant Endocrine Histopathologist, CUHFT:<br>University of Cambridge<br>Addenbrookes Hospital,<br>Cambridge

	CB2 2QQ
	**Professor K Cruickshank & Professor P Chowienczyk**, Clinical Pharmacology<br>Professor of Cardiovascular Medicine & Diabetes; Franklin-Wilkins Building level 4 & Hon Consultant Physician, St Thomas' & Guy's Hospital, King’s College & King’s Health Partners, 150 Stamford Street, London SE1 9NH, UK.
	**Professor D Webb,** Clinical Pharmacology, University of Edinburgh
	**Dr J Buscombe,** Consultants in Nuclear Medicine and PET, Cambridge University Hospital Foundation Trust (CUHFT).<br>University of Cambridge<br>Addenbrookes Hospital,<br>Cambridge<br>CB2 2QQ
	**Dr B Koo,** Consultant Interventional Radiologist, Cambridge University Hospital Foundation Trust (CUHFT):<br>University of Cambridge<br>Addenbrookes Hospital,<br>Cambridge<br>CB2 2QQ
	Professor T Macdonald,<br>University of Dundee<br>Medicines Monitoring Unit and Hypertension Research Centre<br>Medical Research Institute<br>University of Dundee<br>Ninewells Hospital & Medical School<br>Dundee<br>DD1 9SY<br><br>Tel: +44 (0) 1382 383119
	**Dr M Levy,** Consultant Endocrinologist, University Hospitals of Leicester: Lead Endocrinologist for adrenal disorders.
**Study-Coordinator**	Jackie Salsbury<br>The Barts Heart Centre,<br>William Harvey Research Institute,<br>Queen Mary University of London,<br>Charterhouse Square,<br>London EC1M 6BQ

	j.salsbury@qmul.ac.uk
**Statistics**	**Dr A McConnachie,** Assistant Director of Biostatistics, Robertson Centre for Biostatistics, University of Glasgow<br>G12 8QQ<br>Tel: 0141 330 4744
**Data Centre**	Robertson Centre for Biostatistics<br>University of Glasgow<br>Glasgow<br>G12 8QQ<br>Tel: 0141 330 4744

3.0 Protocol Synopsis

**Short Title of clinical trial**	IS Metomidate PET-CT superior to Adrenal venous sampling in predicting ouTCome from adrenalectomy in patients with primary Hyperaldosteronism (MATCH)

**Sponsor name**	Queen Mary University of London (QMUL)

**Research Identification Number**	011149

**Medical condition or disease under investigation**	Hypertension/hyperaldosteronism/adenoma

**Purpose of clinical trial**	To improve prediction of outcomes from surgical intervention in patients with Primary aldosteronism, and evaluate the merits of non-invasive metomidate PET CT versus adrenal vein sampling in the diagnosis of surgically correctable aldosteronism.

**Primary objective /aim**	To assess the aldosterone/renin ratio and blood pressure response of patients with primary hyperaldosteronism recommended for surgery or continued medical management following diagnostic testing with 11C-metomidate PET-CT and adrenal venous sampling, and to compare the ability of the two diagnostic modalities to distinguish between unilateral and bilateral aldosterone-producing adenoma.

**Secondary objective/aims (s)**	1. To investigate whether measurements during 11C-metomidate PET-CT or AVS improve prediction of outcome after surgery.<br><br>2. To investigate whether A blood pressure response to therapy with spironolactone/eplerenone improve prediction of outcome after surgery<br><br>3. To investigate whether demographic, genotypic, and pathological data, improve prediction of outcome after surgery<br><br>4. To measure the proportion of patients who have concordant findings on 11C-metomidate and 18F-CETO PET CT<br><br>5. To measure the proportion of patients in whom 11C-metomidate and 18F-CETO predict the same outcomes after surgery

**Study Design**	This is an observational cohort study which takes place within existing secondary and tertiary care practice of the NHS. It is a multi-centre study in which all patients have the same two investigations performed in random order. These are as follows:<br><br>**<u>11C- metomidate PET CT</u>**<br>This is a one-hour non-invasive study, prior to which patients are treated with dexamethasone for 3 days.<br><br>**<u>[18F]CETO PET CT in final 25 patients</u>**<br>Is a non-invasive procedure This cohort of patients will each have adrenal vein sampling, one[18F) CETO PET CT and one 11C-Metomidate scan. The scans and adrenal vein sampling will be separated with a minimum of one week's interval. For patient convenience we will aim to undertake the (18F) CETO PET Scan on the same afternoon as the 11C-Metomidate PET CT scan. In this case, only 1 x 3 day course of dexamethasone pre-treatment regimen is required, with a final dose taken around midday, between the two scans. If logistically it is not feasible to do both scans on the same day, options will be either for the patient to be scanned the next day; or the patient returns on another occasion, at least 1 week later. Both are recognised scenarios with 11C-metomidate, if synthesis of the isotope failed at the initial visit. Either the dexamethasone is taken for 1 further day (4 doses), or discontinued and taken for a further 3 days prior to the second visit. No adverse consequence of either scenario has occurred during MATCH, or several previous years' experience of undertaking this scan.

	**<u>Adrenal Vein Sampling</u>**<br>This is an invasive investigation in which both adrenal veins are cannulated and blood collected for measurement of adrenal steroid hormones. Adrenocorticotrophic hormone (ACTH) is administered prior to AVS in order to ensure steroid hormone secretion during the procedure.<br><br>We will standardise the start-time of treatment with spironolactone/eplerenone, which is first-choice treatment for patients with primary aldosteronism, in order that the pre-spironolactone blood pressure and biochemistry can be compared with subsequent measurements post-surgery, and the changes during the first month of spironolactone/eplerenone treatment can be used to assess their value in predicting response to surgery.<br><br>**<u>Sub-study of Repeat Metomidate PET CT before and after Spironolactone/eplerenone therapy</u>**<br><br>In order to determine whether it will be necessary for the start of spironolactone/eplerenone treatment to be delayed in all patients until after both investigations are completed, we will perform a sub-study, early in MATCH, in which 6 patients have their PET CT repeated after at least 6 weeks treatment with spironolactone/eplerenone. (This is the conventional period of time for which spironolactone/eplerenone is withdrawn prior to AVS.)

	**Follow-up management and investigations**<br>We anticipate ~50% of patients will be found on one or both investigations to have unilateral PA, and be recommended for adrenalectomy.<br>The primary outcome measurements will be at 6 months after surgery.

**Study**<br><br>**Outcome Measures**	**<u>Primary outcomes</u>**:<br><br>1. Change in ARR from baseline to 6 months after treatment<br>2. Change in SBP (mean of at least 12 measurements from 3 days' home monitoring), from baseline to 6 months after treatment.<br><br>**<u>Secondary outcomes</u>**<br><br>**1.** Cure of hypertension, defined as the proportion of patients in whom average home blood<br>Pressure measurements 6 months post adrenalectomy are <140/90 mmHg on no treatment.<br><br>**2.** Prediction of cure of hypertension, by one or more of:<br>a) The ratio of SUVmax between tumour and normal adrenal;<br>b) Blood pressure fall on spironolactone/eplerenone (50 mg daily for 2 weeks, 100 mg daily for 2<br>weeks), measured as the difference in home systolic blood pressure<br>c) Post-operative analyses of the APA, to include genotyping for somatic mutations in<br>KCNJ5, CACNA1D, ATP1A1, ATP2B3, CTNNB1 and<br>phenotyping by histological grading (zona fasciculata<br>versus zona glomerulosa), gene expression and<br>immunohistochemistry.<br>**3.** Change in home blood pressure from baseline at<br>follow-up visits other than 6 months post-adrenalectomy<br><br>4. Reduction in number of antihypertensive drugs

	5. Change from baseline in blood levels of Troponin, Brain Natriuretic Peptide. There is an awareness that these biomarkers may be elevated in hyperaldosteronism and patients will be reviewed with clinical history and symptomology to assess if there are any associated cardiovascular or other pathology as per usual clinical care.<br><br>6 Cardiac MRI changes in heart structure, anatomy and blood flow from baseline to six months.<br><br>7. Changes in Quality of life from baseline to six months.<br><br>The judgement as to which patients have met primary and secondary outcomes will be made by a critical endpoints committee meeting every 3-6 months throughout the study.

**Sample Size**	The sample size calculation has been performed to permit detection of a significant influence on outcome where the smallest number of patients will be available. This is the group of patients in whom the two diagnostic techniques give discordant results, estimated to be ~20% of the total.<br><br>Recruitment of 165 patients across 3 centres over 3 years (<1/centre/month), with an estimated 90 proceeding to adrenalectomy, permits 90% power at alpha=0.01 of detecting superiority of all hierarchical primary outcomes. This sample size will also provide 90% power to show non inferiority of PET CT in relation to AVS within a margin of 18%.

**Study Sites**	Approximately 3 sites in the UK

**Summary of eligibility criteria**	**Inclusion Criteria**<br><br>• Male or female: Age >18 yrs.<br>• Diagnosis of PHA based on current published Endocrine Society consensus guidelines (Funder et al 2016)<br><br>**Patients will be enrolled/consented when they have had <u>each</u> of the following:**<br><br>At least one paired measurement of plasma renin and aldosterone, measured off spironolactone/eplerenone, showing an elevated ARR. With<br><br>1. *either* a plasma aldosterone >190 pmol/L after saline infusion<br><br>2. *or* 'spontaneous hypokalemia + plasma renin below detection levels + plasma aldosterone > 550 pmol/L)' (as per Endocrine Society guidance, 2016)<br><br>3. *or* failure to suppress plasma aldosterone by 30% + persistent PRA suppression after oral administration of captopril (as per Endocrine Society guidance, 2016)<br><br>**and** a CT or MRI scan of the adrenals with probable or definite adenoma(s) within the last five years

	**Patients with elevated ARR can be put forward for consideration by the MDT as exceptional cases in whom spironolactone/eplerenone is not (fully) withdrawn, and/or saline suppression is not performed, IF:**<br><br>1. Plasma Aldosterone > 450 pmol/L **AND** plasma renin <0.5 pmol/ml/hr (<9 mU/L) if measured on treatment with ACEI (Lisinopril >=20 mg or equivalent) or ARB (Losartan 100 mg or equivalent); OR<br>**2.** Age <40 **AND** definite adrenal adenoma on CT or MRI<br><br>**Patients whose CT/MRI does not show probable or definite adenoma must also be reviewed by MDT before enrolment/consent**<br><br>Any exception to the above diagnostic criteria will be subject to approval by monthly MDT.<br><br>**Exclusion Criteria**<br>• Those patients who indicate that they are unlikely to proceed with surgery will not be recruited, because there will be no outcome change in blood pressure, restoration of normal renin/angiotensin physiology) against which to compare the accuracy of the two Investigations.<br>• Patients contraindicated for spironolactone or eplerenone therapy.<br>• Any patients continuing on beta-blockers or direct renin blockers .

	• Patients with eGFR <30 ml/min/ or expected to have a reduction in eGFR<30ml/min on aldosterone antagonist therapy.<br>• Pregnant / breastfeeding females unable/unwilling to take secure contraceptive precautions whilst undergoing investigations.<br><br>• Patients unwilling/unable to take the dexamethasone required to prepare for a metomidate PET-CT scan.<br>• Patients unwilling to have both 11- C Metomidate PET CT scan and Adrenal Vein Sampling.<br>• Any illness, condition or drug regimen that is considered a contraindication by the PI

**Procedures:**<br><br>**Screening & enrolment**	• Informed consent<br>• Demography<br>• Ethnicity<br>• Weight<br>• Height<br>• Medical History, Concomitant medications<br>• Clinic blood pressure in triplicate, after 5 minutes seated rest.<br>• Blood samples for electrolytes, bicarbonate, renin mass and renin activity, aldosterone and renal function<br>• Urinalysis 24 hours electrolytes Sodium and Potassium<br>• A 24 Urine bottle provided to patient for electrolytes and metabolomic sample<br>• HCG for women of child-bearing potential<br>• Inclusion/exclusion criteria checks<br>• Subject identification number will be issued and patient notified the order of investigations, Patients allocated to the 11-C-metomidate PET CT scan with an additional [18F]CETO for n=25 patients, first will proceed as follows:<br>• Blood pressure monitor provided with training on its use with Pt Blood pressure diary. 3 readings are to be taken morning and evening for 4 days before each clinic visit.<br>• Date provided for PET CT scan appt.<br>• Provision of prescription for 0.5mg Dexamethasone with instructions to take 6hrly 3 days before scan. A surplus of tablets to cover for 5 days supply if required.

**11-Cmetomidate PET CT scan/Adrenal Vein sampling**	• If patient is on a MR (spironolactone/eplerenone antagonist this will usually need to be stopped 6 weeks before going onto AVS sampling.<br>• Correct potassium depletion following local guidelines.<br>• Patient enrolled onto Ecrf with PET CT and AVS order of allocation determined by minimisation<br><br>**Prior to 11-C-metomidate PET CT and [18F]CETO,PET CT scan:**<br>• 0.5mg Dexamethasone to be taken 6hrly 3-5 days before scan<br><br>**Prior to AVS:**<br>• Spironolactone/eplerenone will usually be stopped 6 weeks before;<br>• Correct potassium depletion, following local guidelines. Bloods for ACTH, cortisol, electrolytes with bicarbonate, urea, creatinine, plasma renin mass and aldosterone<br><br>**Treatment:**<br>• 11-C metomidatePET CT scan and AVS results reviewed at specialist MDT<br>o If lateralisation occurs, patient informed and referred for adrenalectomy<br>o Post-operative analyses of aldosterone-producing adenoma (genotyping and somatic mutations)<br>• All patients continue/commence treatment with spironolactone/eplerenone

**Baseline visit**	• Inclusion/exclusion criteria checks<br>• Concomitant medications<br>• Clinic BP, using the same provided bp monitor as for home BP readings, in triplicate, after 5 minutes seated rest.<br>• Four days of Home blood pressure recordings<br>• Blood samples for electrolytes, bicarbonate, serum magnesium, renin mass and renin activity, aldosterone and renal function, high sensitivity troponin, Brain naturetic peptide<br>• 12-lead ECG If not done at screening)<br>• 10mls or urine for steroid metabolomics<br>• Home blood pressure and HR monitoring provided with training and study Bp monitor provided (validated microlife monitor or BHIS listed home blood pressure monitor<br>• Pharmacogenetics, 9mls of serum and plasma to be stored at -80 °C<br>• Home blood pressure monitoring at baseline and repeated at spironolactone visits week 2 and 4 then 4weeks post-surgery visit. Followed by 3months, 9months, 1 and 2-year post surgery visits. For non-surgical patients, a 9-12 month with a subsequent visit up to 2 years.<br>• Pharmacogenetic, serum and plasma sample<br>• Cardiac MR scan<br>• Quality of Life Questionnaire<br>•

**Study visits (See also 2.3: Schedule of Assessments and Procedures)**	Patients will have 10 scheduled visits during the study. Screening visit and one for each PET CT AVS followed by Baseline visit and again at 2 and 4 weeks following start of aldosterone antagonist therapy either spironolactone/eplerenone. Those continuing on medical treatment alone will have one or two further visits between 9 months and 2 years following MDT decision. Those proceeding to adrenalectomy will have as many as possible of the following visits compatible with the overall study end 4 years after study-start: at 1, 3, 6, 12 and 24 months after adrenalectomy.

**Treatment at study end**	It is expected that post adrenalectomy patients will require either no antihypertensive medications or reduced antihypertensive medication which is tailored to their requirements at various points post operatively. For patients who do not proceed to surgery, it is expected that they will continue medical treatment with spironolactone, with change to eplerenone and/or addition of amiloride, if indicated by blood pressure or side effects.

**Procedures for safety monitoring during trial**	Serious adverse event reporting, symptom monitoring, BP monitoring and blood tests for creatinine and electrolytes.

**Criteria for withdrawal of patients on safety grounds**	New diagnosis which contra-indicates adrenalectomy.

**Adverse events and Serious Adverse events**	Selected adverse and all serious adverse events will be recorded onto study database and reported to the sponsor Queen Mary University of London. Refer to SAE/A/E section 12

**Direct access to source data / documents**	The investigators will permit trial related monitoring, audits, REC review, regulatory inspections and following patients informed consent.

**Ethical considerations**	All patients will freely give their informed consent to participate in the study. A patient may decide to withdraw from the study at any time without prejudice to their future care.<br><br>This study protocol has been reviewed and authorised by Dulwich Research Ethics Committee.

**Indemnity**	Negligence in protocol writing will be covered by QMUL (Queen Mary University of London insurers. Negligence in execution of the protocol will be covered by QMUL insurers.<br>There will be no non-negligence cover.

**Emergency contacts**	Local PI at each site, as above.

3.1 Schedule of Assessments and Procedures

Time Point	SCREEN	**C-11 Metomidate PETCT**	**[18F]CETO PET CT** ****	**AVS**	**BASELINE**	2wk Post spiro 50mg	2wk Post spiro 100mg	MDT** Meeting Pts for surgery only continue to 4 weeks'	4weeks post-surgery	3 months post-surgery	6months post surgery<br>Or 9-12 months after end of spironolactone testing for [illegible]	9 months	1-year post surgery	2-year po[illegible] surgery
Informed consent	X													
Medical history	X													
clinical examination	X													
Concomitant medications	X													
weight	X													
Ethnicity	X													
Inclusion/ Exclusion	X													
Clinic BP/HR	X				X	X	X		X	X	X		X	X

Home BP and HR					X	X	X		X	X	X		X	X
12 lead ECG					X						X			
Dexamethasone 6hrly 3 days prior to PET CT		X	X											
avs samples. Aldosterone cortisol				X										
Blood tests – electrolytes with bicarbonate, urea, creatinine, before scan	X	X			X	X	X		X	X	X		X	X
ACTH (before scan)		X	X	X										
High-sensitivity Troponin					X					X	X			X
BNP					X					X	X			X
Blood tests – plasma, renin activity, Aldosterone	X	X	X		X				X	X	X		X	X
Renin mass					X						X			
Serum Cortisol		X	X											
harmacogenetics[1]	X													
24 hour urinary electrolytes sodium/potassium					X									
erum magnesium					X						X			
Urinalysis	X													X

1

24hr Urine Steroid Profile					x						x			
HCG testing for CBP	X	X	X	X	X	X	X		X	X	X		X	X
2mls sample plasma collected EDTA and frozen					X									
Serum sample collected spun and frozen					X									
CMR surgical***					X						X			
CMR non-surgical***												X		
QoL Questionnaire					X						X			
									X					

*For patients not having surgery next study visit will be 9-12 months and another up to 2 years following MDT review of PET CT/or and Adrenal Vein Sampling
** There will be monthly MDTs, with the aim of having a decision for most patients that is discussed with them at their visit after 4 weeks of spironolactone/eplerenone.
*** CMR can be done any time before spironolactone/eplerenone treatment and following 6 weeks withdrawal from amiloride, eplerenone and/or spironolactone.
Follow up 6 month CMR scan can be done after 6 month time point but before the 12 month time point. The cohort of n=25 having (18 F) CETO, , 11C-metomidate and AVS no CMR.

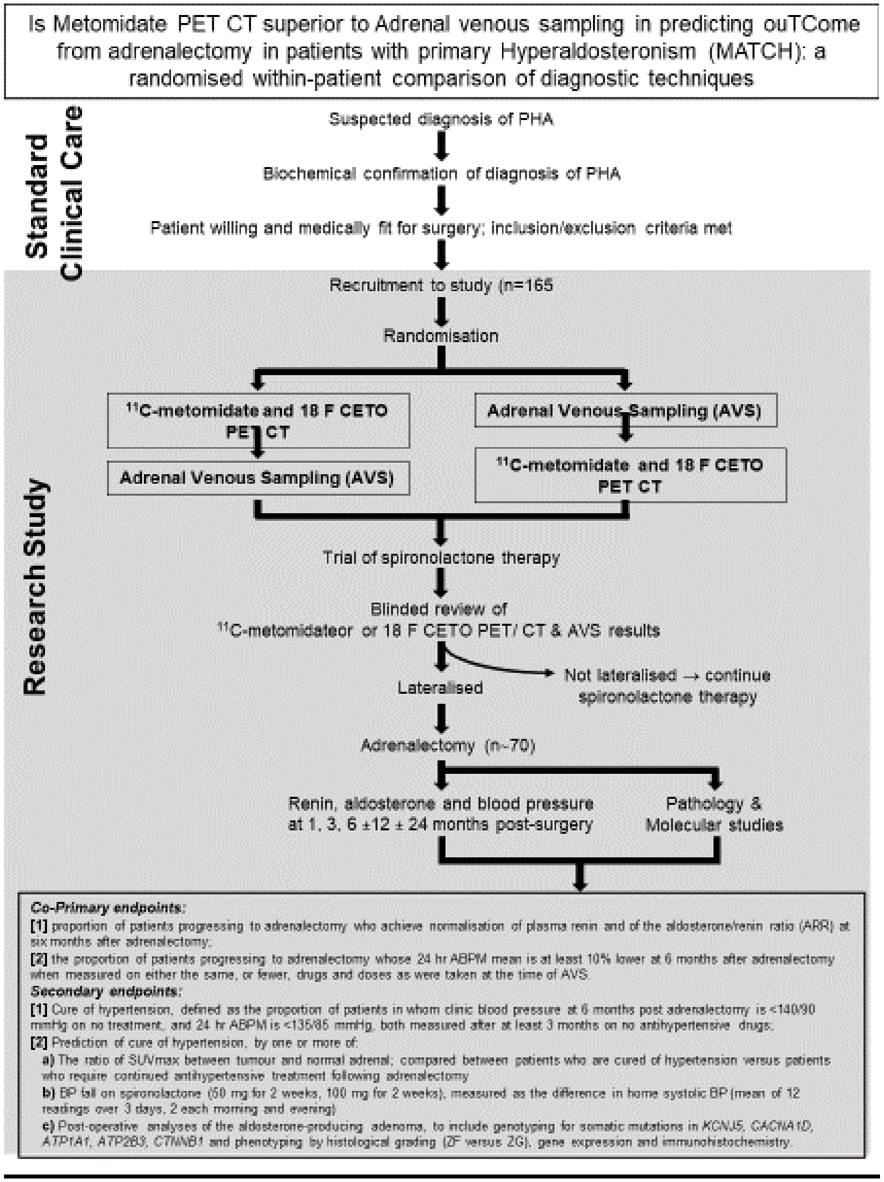
Is Metomidate PET CT superior to Adrenal venous sampling in predicting ouTCome from adrenalectomy in patients with primary Hyperaldosteronism (MATCH): a randomised within-patient comparison of diagnostic techniques
Standard Clinical Care
Suspected diagnosis of PHA
Biochemical confirmation of diagnosis of PHA
Patient willing and medically fit for surgery; inclusion/exclusion criteria met
Research Study
Recruitment to study (n=165
Randomisation
11C-metomidate and 18 F CETO PET CT
Adrenal Venous Sampling (AVS)
Adrenal Venous Sampling (AVS)
11C-metomidate and 18 F CETO PET CT
Trial of spironolactone therapy
Blinded review of 11C-metomidateor or 18 F CETO PET/ CT & AVS results
Not lateralised → continue spironolactone therapy
Lateralised
Adrenalectomy (n~70)
Renin, aldosterone and blood pressure at 1, 3, 6 ±12 ± 24 months post-surgery
Pathology & Molecular studies
Co-Primary endpoints:
[1] proportion of patients progressing to adrenalectomy who achieve normalisation of plasma renin and of the aldosterone/renin ratio (ARR) at six months after adrenalectomy;
[2] the proportion of patients progressing to adrenalectomy whose 24 hr ABPM mean is at least 10% lower at 6 months after adrenalectomy when measured on either the same, or fewer, drugs and doses as were taken at the time of AVS.
Secondary endpoints:
[1] Cure of hypertension, defined as the proportion of patients in whom clinic blood pressure at 6 months post adrenalectomy is <140/90 mmHg on no treatment, and 24 hr ABPM is <135/85 mmHg, both measured after at least 3 months on no antihypertensive drugs;
[2] Prediction of cure of hypertension, by one or more of:
a) The ratio of SUVmax between tumour and normal adrenal; compared between patients who are cured of hypertension versus patients who require continued antihypertensive treatment following adrenalectomy
b) BP fall on spironolactone (50 mg for 2 weeks, 100 mg for 2 weeks), measured as the difference in home systolic BP (mean of 12 readings over 3 days, 2 each morning and evening)
c) Post-operative analyses of the aldosterone-producing adenoma, to include genotyping for somatic mutations in KCNJ5, CACNA1D, ATP1A1, ATP2B3, CTNNB1 and phenotyping by histological grading (ZF versus ZG), gene expression and immunohistochemistry.

4.0 Rationale

4.1 Medical Background

Hypertension is a major risk factor for heart attacks and stroke. Primary hyperaldosteronism (PHA) is an important cause of hypertension and is characterized by excessive aldosterone production by one or both adrenal glands. Previously thought to account for less than 1% of cases of hypertension, there is now general consensus that this is an underestimate, with various series reporting a prevalence of 6-15% in unselected hypertensive populations and up to 20-25% of individuals whose blood pressure is resistant to therapy.(1-5) Uncontrolled hypertension *per se* is thought to cause 350 preventable strokes and major cardiac events per day in the UK but, in addition, PHA itself carries significant cardiometabolic risk over and above that conferred by elevated blood pressure alone.(6) Stroke, myocardial infarction and atrial fibrillation are increased 4 to 12 fold compared to age, gender and blood pressure-matched subjects with essential hypertension. Recent surveys suggest that ~12 million people in the UK have hypertension and over 1 million are therefore likely to have PHA-mediated hypertension.(7) This figure is in line with the recently reported estimate for the USA of at least 10 million affected Americans.(8)

Although various rare forms of PHA exist, the vast majority of cases are accounted for by either bilateral adrenal hyperplasia (BAH) or (in approximately 50% of cases) a unilateral aldosterone-producing adenoma (APA). Distinguishing unilateral from bilateral disease is important, as the ideal (potentially curative) treatment for an APA is surgical removal by laparoscopic adrenalectomy (a low-morbidity procedure in experienced surgical hands), whereas BAH is most appropriately managed medically with aldosterone antagonists.(9-11) Using even the most conservative of the above prevalence estimates, at least 360 000 people in the UK (and probably closer to 500 000) will have a unilateral APA as the cause of their hypertension, making it easily the most common cause of surgically curable hypertension.

Current deficiencies in clinical care
In recent years detection rates of PHA in both primary and secondary care have improved significantly, largely consequent upon the greater availability of high throughput renin assays.(3,12,13) However, in current UK clinical practice, only a tiny proportion of patients with PHA complete the investigational pathway and are referred for potentially curative surgery.

One of the main barriers is the difficulty of proving that only one adrenal gland is the culprit. On diagnostic cross-sectional imaging (CT or MRI), smaller (<1 cm) adenomas may be overlooked (particularly if they lie within the body of the adrenal gland) while, conversely, incidental non-secreting adrenal adenomas occur in 4% of the population over the age of 40 years.(14,15) The latter issue means that in a significant minority of patients with PHA a focal adrenal lesion seen on imaging will not be the source of aldosterone excess and its surgical removal would be inappropriate.

Currently, the test widely considered to be most accurate in distinguishing unilateral (surgically-treated) from bilateral (managed medically) disease is adrenal venous sampling (AVS). This diagnostic procedure, involving the successful cannulation of both 1mm diameter adrenal veins is difficult, time-consuming, invasive, available in only a few centres and has variable success rates, depending on technical expertise and the diagnostic criteria

employed.(16,17) Inevitably, therefore, many PHA patients with surgically-remediable hypertension are suboptimally treated. Hospital Episodes Statistics indicate that fewer than 300 adrenalectomies for PHA are performed in the UK – far behind, *pro rata*, other Western European countries (Reincke M and Plouin F, personal communication from the German Conn's registry and the European Study of Adrenal Tumours). Given the above prevalence figures, this represents a major shortfall in clinical care, with public health consequences that are hard to overestimate. A second reason that physicians are seemingly reluctant to complete the assessment of PHA by looking for a resectable APA is that the chances of 'cure' of hypertension following adrenalectomy are difficult to predict, making surgical recommendations for individual patients problematic, especially in older patients.(10,11) The purpose of this proposal is to conduct a multi-centre clinical study to provide data that will address both these issues and, thereby, improve the major shortcomings in clinical care currently offered to patients with PHA.

^{11}C-metomidate scanning

Metomidate is a potent inhibitor of CYP11B1 (11β-hydroxylase) and CYP11B2 (aldosterone synthase). It is the methyl analogue of etomidate, an ethyl-imidazole anaesthetic agent which is used clinically in sub-hypnotic doses as an inhibitor of adrenal steroidogenesis in the management of refractory Cushing's syndrome.(18,19) Metomidate (itself used as a veterinary anaesthetic until the 1970's) can be $C^{11}H_3$-labelled as a PET radiotracer (^{11}C-metomidate), and proof of concept of its high affinity binding to adrenal cortex was established *in vitro* and in primate studies as far back as 1998.(20) In the first human application, 15 patients underwent ^{11}C-methionine imaging prior to surgery for a unilateral adrenal mass.(21) The nine histologically-diagnosed adrenocortical tumours each demonstrated high uptake and could be reliably discriminated from the six lesions which were not of cortical origin. In a larger follow up series, comprising 73 patients who underwent surgery for suspected adrenal lesions, the six histologically confirmed APAs demonstrated the highest ^{11}C-metomidate uptake, with a standardised uptake value (SUV) of 30.7 compared to 18.4 in non-functional adenomas.(22) When combined with high-resolution CT scanning its sensitivity and precision both increase as the improved spatial resolution permits distinction of tumour from normal adrenal tissue (and separate measurement of SUV in each) with greater confidence.(23)

In a previous, non-randomised proof-of-concept study we showed that ^{11}C-metomidate PET CT scanning is comparable to AVS in distinguishing unilateral from bilateral PHA.(24) In 35 patients with confirmed PHA and an anatomical adrenal abnormality, ROC analysis for PET-CT showed that the finding of >25% higher PET signal (SUVmax - maximum standardised uptake value) over an adenoma compared to the contralateral adrenal conferred 76% sensitivity and 87% specificity compared to AVS; specificity rose to 100% in patients whose absolute SUVmax over tumour was >17. Since then, considerable clinical experience with ^{11}C-metomidate PET-CT scanning has been accumulated, although robust, randomized trial data are lacking to endorse its widespread use as the investigation of choice.(23,25).

[^{18}F]CETO (Chlorofluoroethyl ETOmidate) is a positron emission tomography (PET) - radiopharmaceutical developed for the study of the adrenal glands. It is closely related to metomidate, with replacement of its methyl by chlorofluoroethyl group. It is also closely related to an earlier 18F-analogue, fluoroetomidate (FETO), which was not pursued because of difficulties with synthesis, and higher uptake outside the adrenal than seen with metomidate . [^{18}F]CETO binds to the same two enzymes as metomidate in the adrenal cortex: aldosterone synthase and 11-beta-hydroxylase (the final enzyme in cortisol synthesis) encoded by the enzymes *CYP11B2* and *CYP11B1* respectively. It is administered in a low volume (< 5mL) with a total mass not exceeding

10 micrograms. The amount of administered radioactivity will be in the range 100-200MBq. Study subjects will have a single administration of [18F]CETO. [18F]FETO was previously evaluated in animals and in healthy volunteers (Wadsak et al, 2003; Mitterhauser et al, 2003; Ettlinger et al, 2006; Wadsak et al, 2006). [18F]CETO has also been evaluated in animals and patients with adrenal tumours, and shown to cause no adverse effects and to have no uptake outside the adrenal. [18F]CETO is produced at: Wolfson Brain Imaging Centre (WBIC), University of Cambridge , Cambridge Biomedical Campus, Cambridge, United Kingdom.

4.2 Hypothesis and Novel Aspects of the Trial

Our hypothesis that ^{11}C-metomidate scanning is superior to AVS derives from the findings of our earlier study, and subsequent clinical experience in more than a hundred patients.We now wish to determine whether ^{11}C-metomidate scanning is superior to AVS in identifying unilateral PHA. Despite our experience, most individual centres, and importantly the forthcoming revision to the American Society of Endocrinology Guideline, are waiting for further trial data, albeit acknowledging that PET CT is likely to have a major role in sub-typing of PHA. If superiority of PET-CT can be shown then this, coupled with greater patient preference and convenience, would improve access to diagnosis and will encourage doctors to look for PHA in many more patients than is currently the case. Another major challenge in the management of unilateral PHA is the successful preoperative identification of those patients who are likely to benefit most from adrenalectomy. It is timely therefore to investigate whether ^{11}C-metomidate PET-CT and clinical response to spironolactone, together with demographic, genotypic and pathological data, can provide predictive information as to which patients should be prioritised for surgery. The case that ^{11}C-metomidate PET-CT will be superior to AVS Aside from the obvious advantages of being non-invasive and, potentially, affording greater capacity for investigating PHA (only a handful of interventional radiologists in the UK are able to perform AVS to a good standard), there are several reasons why we consider ^{11}C-metomidate PET-CT is likely to be superior in distinguishing unilateral from bilateral disease. First, international consensus criteria on the interpretation of AVS demand stringent thresholds to reliably establish whether excessive aldosterone production lateralises to one side or the other.(17) Aldosterone (A) and cortisol (C) values are measured in both adrenal veins and in the lower part of the inferior vena cava (IVC), and a 'normalised' aldosterone value (A/C ratio) calculated for each side. Evidence of lateralisation (i.e. 'dominance' of aldosterone production by one adrenal gland) is considered to be present if (i) the A/C ratio on one side is ≥ 4 times that on the other side **and** (ii) the A/C ratio in the 'non-dominant' (or 'suppressed') vein is less than that in the IVC. BAH is diagnosed if the A/C values in each adrenal vein differ by ≤ 2-fold, which leaves an equivocal ('grey') area (A/C ratio of 2-4) in which clinical decision-making is controversial.(26)

As already indicated, given that many physicians are inherently reluctant to pursue PHA investigations to their conclusion, clinical caution dictates that a substantial proportion of patients with ambiguous or non-diagnostic results from initial AVS do not progress to further investigation or surgery. Second, it is an important requirement for AVS that patients

discontinue spironolactone (or other mineralocorticoid receptor antagonists) several weeks before the procedure in order to avoid a false negative result due to re-activation of aldosterone secretion from the contralateral (normal) adrenal. However, patients with difficult-to-control hypertension and/or hypokalaemia are exposed to potential risk by this practice. It is also important to avoid hypokalaemia at the time of AVS, which itself can blunt aldosterone secretion from the APA and thus contribute to a false negative catheter study. For patients who develop marked hypokalaemia on discontinuing potassium-sparing diuretic therapy, the requirement to take large amounts of potassium supplements to achieve normokalaemia is unpalatable and, in many cases, unsuccessful. In contrast, ^{11}C-metomidate scanning does not require the discontinuation of spironolactone Third, it is being increasingly recognised that in a significant proportion of patients, what may have appeared to be a single focal lesion within one adrenal gland is, on histological examination, a series of discrete, smaller nodules.(27,28) The anatomical/functional ‘model’ on which interpretation of AVS is based, is one of a homogeneously draining adrenal effluent, but our *ex vivo* experiments on adrenals removed at surgery clearly indicate these smaller nodules may have different secretory activity. For example, an APA may exist alongside a nodule with high expression of CYP11B1 (encoding the enzyme that produces cortisol).Given the stringent criteria described above, any change in the denominator of A/C has the potential to alter the interpretation of the AVS result and change surgical decision making. Our experience indicates that ^{11}C-metomidate scanning is not subject to this potential confounding factor.(23-25)

Increasingly, we are also using 11C-metomidate PET CT to identify small APAs which were missed altogether on original cross-sectional imaging by CT or MRI (Appendix A Case 4). These small APAs often prove to be zona glomerulosa (ZG)-like on appearance and laboratory analyses, and we believe the high density of metomidate uptake reflects their high density of aldosterone synthase.

Finally, the PET CT also appears valuable in excluding unilateral disease in patients where AVS has been ambiguous or unsuccessful

Pre-treatment with spironolactone as a predictor of outcome following surgery
Aside from the lack of widespread availability of high quality AVS, surgical decision-making in PHA is made more difficult by the lack of a good ‘prediction’ test to indicate who will benefit most from a unilateral adrenalectomy.(11) In younger patients (particularly females planning to conceive), with a shorter duration of hypertension, the avoidance or reduction in number of long-term antihypertensive agents almost always outweighs the temporary discomfort of surgery. However, in older patients the decision may be more balanced, particularly in the presence of comorbidities and if the number of agents required to control blood pressure is modest. We hypothesize that a preoperative therapeutic trial of spironolactone (an aldosterone antagonist), using a strictly defined protocol, will provide information to aid prediction of which patients are likely to derive the greatest benefits in terms of blood pressure control following surgery. Using higher doses, but for a relatively short time period (4 weeks), should permit more complete mineralocorticoid receptor blockade than is often achieved in routine clinical practice, while avoiding the side effects (e.g. gynaecomastia in men; menstrual disturbance in premenopausal women) that are typically associated with long-term use.
^{11}C-metomidate scanning as a tool for predicting outcome following surgery.In addition to the above, it is timely and plausible to explore whether ^{11}C-metomidate PET CT can recognise differing types of APA with intrinsic differences in prognosis. We see these efficacy and mechanism evaluations as the vital middle step in progressing the technique from its BHF-

funded experimental proof-of-concept to HTA-assessment as the nationally (and internationally) preferred technique for optimal and cost-effective decision making in PHA.

Rationale for undertaking tumour genotyping to enable prediction of outcome
Currently, approximately two thirds of unselected APAs harbour somatic mutations in one of several recently identified genes (*KCNJ5, ATP1A1, ATP2B3, CACNA1D, CTNNB1*) (27,29) It remains unclear whether APAs harbouring particular mutations may have inherent differences in their natural history and the clinical outcome following their surgical removal. Our group is developing techniques and protocols (not included in this grant proposal) to identify the presence of mutations in DNA derived from adrenal effluent blood (obtained during AVS – as a proof-of-concept step in the pathway to screening blood from a peripherally collected sample(30)), and from fine needle aspirates obtained by endoscopic ultrasound. Developing a prediction model that includes the retrospective genotype information obtained from the tumours of patients included in this study opens up the potential, in the future, for stratified clinical decision-making using genotypic information obtained by the two novel approaches described.

The potential for 'digital' quality diagnosis, by detection of somatic mutations seen uniquely in unilateral APAs, will be increased if the proportion of APAs with known mutations rises from the current 60-70%, towards 80-90%. A greater variety of mutations would also increase the possibilities for linking outcome to genotype. We therefore plan two complementary pieces of genotyping on each APA. One is targeted sequencing of all known genes with adrenal mutations, using an Ion Torrent Custom Panel, commissioned by Prof Eamonn Maher (Genetics). The remaining samples will be submitted to exome sequencing at the end of the project, by when cost is expected to be as low as the targeted sequencing now costs.

4.3 Risk-Benefit Considerations

Risks: We consider the potential risks to patients who participate in MATCH to be very small. The radiation dose of an ^{11}C-metomidate PET-CT scan has been estimated at 3.5 mSv and /[18F]CETO PET CT scan is estimated at 4.1mSV- slightly higher than a year's exposure to background radiation (2.7 mSv - https://www.gov.uk/government/publications/ionising-radiation-dose-comparisons/ionising-radiation-dose-comparisons), and substantially lower than that associated with a chest CT scan (6.6 mSv) and the minimum level, 10 mSv, to raise any concerns about imaging studies. For the sub study of 6 patients, the dose for the two PET CT scans before and after spironolactone therapy, this exposure is approximately 10.75 millisieverts (mSv) equivalent to 4 years' exposure to background radiation in the UK. This equates to approximately estimated at about 1 in 2100 risk of developing cancer and to put this into context, the natural life time incidence of developing cancer is estimated to be about 1 in 3. For the cohort n=25 patients having 1 diagnostic CT (+ 11C-metomidate + [18F] CETO PET CT + 2x low dose CT) is equal to 12.75mSv. The estimated risk of cancer is 1 in 1,900, the equivalent of 6 years background radiation in the UK.

As described, the group of patients to be studied are those with PHA who indicate a desire to proceed with laparoscopic surgery if the diagnostic tests support a unilateral source of aldosterone excess. The discomfort from surgery is something that would occur with routine clinical management and is not considered to be 'study related'. If our hypothesis is correct, that 11C-metomidate scanning is superior to AVS, then clearly more patients will experience the discomfort of surgery. However, given that the stated goal of the project is to increase the

numbers of patients offered potentially curative surgery prior to the onset of irreversible hypertension we consider this outcome to be a potential benefit rather than a risk.

Benefits: The potential benefit to patients is that, if the hypothesis is correct, more patients will proceed to adrenalectomy than do so based on conventional testing; moreover, at least 50% of these will be cured of their hypertension altogether, and the remainder will require fewer drugs for effective blood pressure control. Therefore, aside from the potential benefits to individuals, society also stands to gain from MATCH with a reduction in the public health burden from poorly controlled hypertension and its sequelae in a large number of patients.

By including the [18F] CETO PET CT scan sub study in approx. n= 25 patients, we hope to demonstrate that by having a tracer with an increased half-life will extend availability to other NHS sites without on-site facilities for tracer synthesis with regard to laterization.

5.0 Objectives

5.1 Assessment of Efficacy and Safety

Efficacy will be determined by changes in ARR and BP following treatment, guided by 11-C-metomidate PET-CT and AVS.

Safety will be assessed by monitoring symptoms, BP, renal function and serious adverse events at each visit.

5.2 Primary Objectives/Aims

To assess the aldosterone/renin ratio and blood pressure response of patients with primary hyperaldosteronism recommended for surgery or continued medical management following diagnostic testing with 11C- metomidate PET-CT and adrenal venous sampling, and to compare the ability of the two diagnostic modalities to distinguish between unilateral and bilateral aldosterone-producing adenoma.

5.3 Secondary Objectives and Aims

To investigate whether measurements during 11C-metomidate PET-CT or AVS improve prediction of outcome after surgery.

To investigate whether blood pressure response to therapy with spironolactone/eplerenone improve prediction of outcome after surgery

To investigate whether demographic, genotypic, and pathological data, improve prediction of outcome after surgery.

To measure the proportion of patients who have concordant findings on 11C-metomidate and 18F-CETO PET CT

To measure the proportion of patients in whom 11C-metomidate and 18F-CETO predict the same outcomes after surgery

5.4 Primary Outcome Measures

1. Change in ARR from baseline to 6 months after treatment
2. Change in SBP (mean of at least 12 measurements from 3 days' home monitoring), from baseline to 6 months after treatment.

5.5 Secondary Outcome Measures

1. Cure of hypertension, defined as the proportion of patients in whom average home blood Pressure measurements 6 months post adrenalectomy are <140/90 mmHg on no treatment.

2. Prediction of cure of hypertension, by one or more of:
a) The ratio of SUVmax between tumour and normal adrenal;
b) Blood pressure fall on spironolactone/eplerenone (50 mg daily for 2 weeks, 100 mg daily for 2 weeks), measured as the difference in home systolic blood pressure
c) Post-operative analyses of the APA, to include genotyping for somatic mutations in KCNJ5, CACNA1D, ATP1A1, ATP2B3, CTNNB1 and phenotyping by histological grading (zona fasciculata versus zona glomerulosa), gene expression and immunohistochemistry.

3. Change in home blood pressure from baseline at follow-up visits other than 6 months post-adrenalectomy.4. Reduction in number of antihypertensive drugs

5. Change from baseline in blood levels of Troponin, Brain Natriuretic Peptide. Change from baseline in blood levels of Troponin, Brain Natriuretic Peptide. There is an awareness that these biomarkers may be elevated in hyperaldosteronism and patients will be reviewed with clinical history and symptomology to assess if there are any associated cardiovascular or other pathology as per usual clinical care.

6 Cardiac MRI changes in heart structure, anatomy and blood flow from baseline to six months.

7. Changes in Quality of life from baseline to six months.

8.
The judgement as to which patients have met primary and secondary outcomes will be made by a critical endpoints committee meeting every 3-6 months throughout the study.

6.0 Trial Design

This is an observational multi-centre prospective cohort study taking place within existing secondary and tertiary care practice of the NHS. Its aims to compare the diagnostic accuracy of AVS and ^{11}C- metomidate PET-CT, performed in random order using minimization allocation .

The Proof of concept in humans has already been demonstrated for 11C-metomidate PET-CT in a published non-inferiority study of 35 patients.(24) This proposal seeks to establish if PET-CT is superior to AVS in its ability to identify surgical candidates.

6.1 11C- metomidate PET CT

The specialised abdominal PET CT scan locates the hot spots of aldosterone synthase activity of the affected adrenal gland. The Standard ^{11}C- metomidate PET CT scan procedure will be manged in specialised sites where a cyclotron is available to synthesise the radionuclide 11- C metomidate .Currently the diagnostic^{11}C- metomidate PET CT scan is manged at the PET CT unit Addenbrookes Hospital, Cambridge University Hospitals Cambridge. However other units are including this technique predominantly in London and hope to join within the next two years. Patients will be recompensed for reasonable travel expenses incurred.

Prior to 11-C metomidate scan PET CT scan patients will be prescribed Dexamethasone for three up to five days prior to the scan; one tablet four times a day (one tablet every 6 hours), with the last dose being taken on the morning of the scan or at midday if the scan appointment is in the afternoon. Dexamethasone supresses the release of cortisol from the adrenal gland allowing aldosterone levels to be measured more accurately by dampening the background noise.

On the day of the scan the patient will be cannulated Baseline bloods will be measured for ACTH, cortisol, electrolytes including urea and creatinine, renin mass, aldosterone and bicarbonate. Following this an Intravenous preparation of 11C-Metomidate radiotracer, after 30 minutes the patients will have the PET CT scan in the specialist unit. 11C-Metomidate has a very short radioactive half-life (20 minutes). This means that there is effectively none of the radiotracer left in the body two hours after the injection.

Dexamethasone: is used diagnostically in clinical practice to assess adrenal gland function. It is commonly used to treat a variety of conditions including allergy. Side-effects usually occur in people who take the medicine long-term with possible side effects including reduced bone density and a compromised immune system. Short term Side-effects are almost always reversible and will go away after treatment is complete. These include indigestion, excess in hunger, changes in glucose levels, changes in mood and difficulty in sleeping. The 11-C metomidate PET CT has been conducted in more than 200 patients, and there have been no reported side effects in these patients from either the dexamethasone or metomidate.

6.2 (18F) CETO

To date this has been administered to nine patients with secretory and non-secretory adrenocortical tumours. It has also been extensively studied ex vivo in a range of secretory adrenal adenomas and carcinomas. Specific [$_{18}$F]CETO uptake was elevated in aldosterone-producing adenomas of all three mutation types (KCNJ5, ATP2B3 and CACNAID). Specific uptake in human- and NHP spleen was lower than in normal adrenal gland and APAs, Specific uptake in PCC, small intestine and kidney was also lower than normal adrenal gland, as was liver uptake

(compared to normal, mutated as well as pathological adrenal gland). [18F]CETO tissue-to-adrenal gland ratio showed a threefold uptake in APAs compared to normal adrenal gland. (WBIC, 2019, Investigator Brochure, V1).

As with 11C-metomidate, its specificity in vivo for CYP11B2 (encoding the enzyme for aldosterone synthesis) is expected to require temporary suppression of cortisol synthesis, by 72 hours of pretreatment by dexamethasone. In this and most other respects the protocol for administration of 18F-CETO will be as for 11C-metomidate, as above. There are two exceptions:
[i] The longer half-life allows patients to be scanned (pictured) at multiple timepoints after a single injection of radioisotope; patients who are able to stay (upto 5 hours) will be invited to have more than one scan (picture), exploring the flexibility and convenience this may provide in the future. The radiation exposure is, of course, the same whether patients have one or several scans (pictures) after an injection
[ii] Serum cortisol will be measured both at the time of the scan (as for 11C-metomidate) and on the day after the scan, whenever this is logistically possible.

6.2 Adrenal Vein Sampling

This test is currently the gold standard diagnostic technique for diagnosing Conn's adenoma The test in carried out in the radiology department by a specialist radiologist. During the first two hours the patient will be cannulated and ACTH (Adrenocorticotrophic hormone) infused in 0.9%saline for 3 hours approximately, commencing half an hour before and continued during the procedure . This is routinely used in the AVS procedure to stimulate the adrenal glands. This maximises the chances of detecting a difference between the two glands and aldosterone production. This is a common clinical diagnostic test in clinical practice.

A small cannulae and catheter are introduced via the groin (usually into the right) to access the blood vessels by the radiologist. Through the needle the catheter is introduced and then positioned in adrenal gland veins. The blood samples from both adrenal veins, and the main vein (inferior vena cava) are used to measure the levels of adrenal hormones, aldosterone and cortisol, and metanephrines These are routine measurements in the AVS procedure, the current gold standard. [Extra blood for experimental measurements such as cell-free DNA, and DPP4 will be taken]. Blood which is not used for these assays will be kept frozen for the development of future tests which may improve our ability to predict outcome from adrenalectomy. These are likely to be additional steroids, secreted proteins, or cell-free RNA or DNA.

Post procedure, patients are to lay flat. During this time, the right groin is routinely inspected to make sure there is no oozing from the site or any significant bruising. Blood pressure and heart rates are monitored. Patient will be discharged by radiologist. The risk of the procedure we are looking to minimise here is excessive bleeding or haemorrhage afterward. This is rare. There is a potential, although very rare of an adrenal infarction.

7.0 Cardiac Magnetic Resonance Imaging Scan (CMR)

All patients will be invited to have a cardiovascular magnetic resonance scan (CMR) at baseline visit which will be repeated at the 6-12 months' time point, following baseline visit. There is an expectation that not all patients will consent to this optional measurement.
CMR uses a powerful magnet and radio waves to form pictures of the heart. Gadolinium-based intravenous MR contrast will be administered via an intravenous cannula for angiography, myocardial perfusion and viability. The incidence of developing adverse events following administration is very low. Most reactions are mild and include warmth, coldness and pain at cannula site, headache, vomiting, parathesias, dizziness and itching. Very rarely, between 0.001% to 0.01% an anaphylactoid reaction may occur. The CMR suite, at St Bartholomew's NHS Trust, have trained clinical staff to manage this condition is it should occur. Patients will have renal function blood tests prior to scan. Patients will be requested to arrive approximately 30 minutes before the actual scan appointment time to perform a brief assessment and change into a gown. The scan itself takes about forty minutes. It involves patient lying in supine position inside a large open-ended tube. Electrocardiogram monitoring will continue during the scan. Patients will be asked to hold breath between 2 and 15 seconds prior to image capture. Patients will be able to communicate the operator anytime via telecon.

8.0 Sub-study of Repeat Metomidate PET CT before and after Spironolactone/eplerenone therapy

In order to determine whether it will be necessary for the start of spironolactone/eplerenone treatment to be delayed in all patients until after both investigations are completed, we will perform a sub-study, early in MATCH, in which 6 patients have their PET CT repeated at least after 6 weeks treatment with spironolactone/eplerenone. (This is the conventional period of time for which spironolactone is withdrawn prior to AVS.)

9.0 Follow-up management and investigations

We anticipate ~50% of patients will be found on one or both investigations to have unilateral PA, and be recommended for adrenalectomy. The primary outcome measurements will be at 6 months after surgery.

9.1 Stopping Criteria

Withdrawal criteria for individual patients are shown in section 4.1 page 26 below. Criteria for the whole trial will be determined by the Trial Steering Committee (TSC), or delegated (if deemed appropriate by the TSC) to an independent DMEC (see [12]). Because neither technique is new, it is highly unlikely that any stringent safety boundary that is set will be crossed. Any stopping rules will need to acknowledge that there is a long lag between the interventions and the assessment of outcome: at least 9 months, given the 6-month post-operative timing of primary assessment, the interval between diagnostic intervention and surgery, and frequency of the endpoint committee assessments.

9.2 Study population

The potential constituency of patients at which MATCH is directed is the majority of all patients with PHA who indicate a desire to proceed to surgery if lateralisation can be confidently shown. The diagnostic criteria for PHA used in all participating centres will be drawn from the recently published updated Endocrine Society consensus guidelines (2016).(31) All patients will have had standard cross-sectional imaging by CT or MR scanning. If patients have had 11- C Metomidate scan and AVS with or without baseline visit they will be included in ITT analysis

10.0 Inclusion/Exclusion criteria*:*

10.1 Inclusion Criteria

- Male or female: Age >18 yrs.
- Diagnosis of PHA based on current published Endocrine Society consensus guidelines (Funder et al 2016)

- **Patients will be enrolled/consented when they have had each of the following:**

At least one paired measurement of plasma renin and aldosterone, measured off spironolactone/eplerenone and other polluting medications, showing an elevated ARR and.

1. *either* a plasma aldosterone >190 pmol/L after saline infusion
2. *or* 'spontaneous hypokalemia + plasma renin below detection levels + plasma aldosterone > 550 pmol/L)' (as per Endocrine Society guidance, 2016)
3. *or* failure to suppress plasma aldosterone by 30% + persistent PRA suppression after oral administration of captopril (as per Endocrine Society guidance, 2016)

and a CT or MRI scan of the adrenals with probable or definite adenoma(s) within the last five years

- Patients with elevated ARR can be put forward for consideration by the MDT as exceptional cases in whom spironolactone/eplerenone is not (fully) withdrawn, and/or saline suppression is not performed, IF**:**

1. Plasma Aldosterone > 450 pmol/L **AND** plasma renin <0.5 pmol/ml/hr (<9 mU/L) if measured on treatment with ACEI (Lisinopril >=20 mg or equivalent) or ARB (Losartan 100 mg or equivalent); OR

2. Age <40 **AND** definite adrenal adenoma on CT or MRI

Patients whose CT/MRI does not show probable or definite adenoma must also be reviewed by MDT before enrolment/consent

- All patients will have a positive Aldosterone renin ratio (ARR) serum measurement with another local diagnostic confirmatory test as specified from local specialised APA Guidelines. This is often standard cross-sectional imaging by CT or MR scanning. Any exception to recommended diagnostic criteria will be subject to approval by monthly MDT.

10.2 Exclusion Criteria

- Those patients who indicate that they are unlikely to proceed with surgery will not be recruited, because there will be no outcome change in blood pressure, restoration of normal renin/angiotensin physiology) against which to compare the accuracy of the two Investigations. Patients contraindicated for spironolactone/eplerenone therapy use.
- Any patients continuing on beta-blockers or direct renin blockers.
- Pregnant patients or women unable/unwilling to take secure contraceptive precautiions whilst undergoing investigation.
- Patients unwilling/unable to take the dexamethasone required to prepare for a metomidate PET-CT scan
- Patients unwilling to have both 11- C Metomidate PET CT/ scan and Adrenal Vein Sampling.
- Any illness, condition or drug regimen that is considered a contraindication by the PI

NB: Patients unable to have baseline cardiac MRI due to claustrophobia per se may still be to included in the MATCH study at the PI discretion.

All other patients with PHA however, are potential candidates for inclusion in MATCH, including those with apparently normal bilateral adrenal morphology, given that small APAs lying within the body of the adrenal may not be seen on even high resolution CT scanning.

10.3 Withdrawal Criteria

Unwillingness or inability to undergo both PET CT and AVS or at the PI discretion.

11.0 Diagnostic Investigations

The primary control intervention is AVS and will be performed according to an identical protocol

at all centres whose internal audit data indicate a successful bilateral cannulation rate of >75%. Briefly, the femoral vein is cannulated under local anaesthesia and catheters advanced under fluoroscopic guidance into each adrenal vein.(17) The procedure is performed under conditions of ACTH stimulation, which facilitates unequivocal confirmation of secure adrenal vein cannulation. Blood samples from both adrenal veins and the lower part of the inferior vena cava are analysed for aldosterone (A) and cortisol (C) and an aldosterone/cortisol (A/C) ratio calculated at each site. Lateralisation (providing evidence for an adrenalectomy to be recommended) is deemed to exist if the A/C value in one adrenal vein is at least 4-fold higher than the other. In preparation for AVS, treatment to control blood pressure is limited to a non-dihydropyridine calcium antagonist (e.g. verapamil) and doxazosin (together with potassium supplementation as needed), as the treatments least likely to interfere with interpretation of aldosterone/renin measurements. Although some rise in blood pressure is permitted in preparation for AVS, severely hypertensive patients may require additional therapy according to clinical judgement.

The experimental intervention is ^{11}C-metomidate PET-CT. There has been no change in the protocol used since the proof-of-concept study. Patients are treated with dexamethasone 0.5 mg 6-hourly for 3 days, then receive an injection of 250 mBq (on average) of ^{11}C-metomidate, with CT and PET scanning of the adrenal area 20-30 minutes later. A positive (lateralization) result is indicated by a maximum standardised uptake at least 25% greater in one adrenal gland compared to the other.

The assessment of the two diagnostic modalities will be undertaken at a monthly MDT of endocrinologists and radiologists. Patients will be offered surgery if one or both techniques indicate unilateral disease. For the very small number of cases (we estimate 1 in 25 on the basis of the proof-of-concept study), where AVS and ^{11}C-metomidate PET-CT each meet these criteria for lateralisation, but to opposite sides, we will defer to the Trial Steering Committee (TSC) (see section 11).

The primary outcome is cure of PHA by unilateral adrenalectomy and will be determined by an endpoint committee blind to results of the prior imaging tests.

The secondary intervention is a systematic evaluation of spironolactone eplerenone therapy, as a predictor of primary or secondary outcomes. After both diagnostic investigations (AVS and ^{11}C-metomidate PET-CT) have been performed, spironolactone/eplerenone will be prescribed in a standardised fashion with home blood pressure monitoring before and after four weeks of treatment (50 mg/day for two weeks, force-titrated to 100 mg/day after two weeks unless SBP is <130 mmHg on repeat home monitoring, and/or plasma K+ is >5.0).

In addition to the clinical interventions, we plan a number of laboratory procedures to confirm that the adenoma(s) removed at surgery were aldosterone-secreting, and to look for genetic and/or biochemical differences between APAs. These differences, if observed, will then be correlated with either prior differences in the ^{11}C-metomidate PET-CT signal and/or the subsequent outcome from surgery. The techniques for these laboratory procedures (immunohistochemistry for CYP11B1 and CYP11B2, qPCR for *CYP11B1*, *CYP11B2*, *CYP17A1*, and primary cell culture for measurement of aldosterone secretion) are all well-established in our laboratory. Targeted sequencing of genes with known somatic mutations will also be performed, as described above.

12.0 Requirements for Participating sites and Investigators.

All participating investigators must agree to conduct the trial in accordance with the European Clinical Trials Directive 2001/20/EC, local law, the declaration of Helsinki including all revisions, and the study protocol.

Participating sites must have the facilities and resources required for the conduct of the various defined study tests and procedures. For the specialised 11 C -metomidate scan patients will be referred to Addenbrookes Hospital Cambridge PET CT Unit.

Training materials will be provided for use of the eCRF system, plus any study-specific procedures as required.

12.1 Trial Sites and Numbers of Participants

A total of 165patients will be recruited into the study, from approx. three to six study sites within the UK depending on success on patient recruitment. Recruitment will continue until at least 70 patients have proceeded to adrenalectomy, even if we exceed a total of 165 patients being consented and undergoing investigations. This allows for some patients who may withdraw between consent and completion of investigations and/or surgery.

12.2 Expected duration of the clinical research.

In total, each participant will be in the study for 2.5 years (including follow up period of 24 months). It is estimated that the total duration of the trial will be 4 years.

13.0 Premature site closure

If protocol compliance is found to be poor at a particular site, or if the data quality is found to be unacceptable, efforts will be made to eradicate the problem (e.g. by additional training). However, if problems persist in spite of these efforts, premature closure of the site will be considered.

Premature closure of a site will also be considered if recruitment is particularly poor at that site.

Future follow-up and treatment of study participants from that site will be agreed by the Steering Committee.

13.1 Premature termination of the clinical research

Premature termination of the trial will be considered in the event of

- new insight from other trials
- insufficient recruitment rates

The decision to terminate the trial will be taken by the Trial Steering Committee.

14.0 Selection of the Participants

Patients will be identified from endocrine/ hypertension clinics
NB: All other patients with PHA however, are potential candidates for inclusion in MATCH, including those with apparently normal bilateral adrenal morphology, given that small APAs lying within the body of the adrenal may not be seen on even high resolution CT scanning.

14.1 Screening for eligibility

Examination of the inclusion and exclusion criteria of potentially eligible patients will be performed using a predefined screening log. All screened patients, whether eligible or not, should be included in the screening log, with reason for ineligibility recorded as appropriate.

14.2 Informed Consent

Written informed consent is required before any study-related procedures are carried out.

The patient will be sent the Patient Information Sheet in advance of their visit. The investigator/Research nurse will explain to the potential participant the purpose of the trial, any potential risks involved, the nature of the intervention, and the duration of trial participation. The patient will be informed that participation in the trial is voluntary, and that they may withdraw at any time without compromising either their future care or their relationship with their responsible physician.

The patient will be given time to consider their decision, and to discuss with the study doctor and nurse any questions about the study. If the patient is willing to participate in the trial, the study nurse will obtain the patient's informed consent: the patient will sign a consent form for study participation. The original signed consent form will be retained in the study site file, with copies added to the patient's medical notes and provided to the patient, together with the patient information sheet.
Patients unable to have cardiac MRI due to claustrophobia per se can still consent to MATCH study minus the Cardiac MRI.

14.3 Withdrawal of Informed Consent

In the event that a patient wishes to withdraw consent for participation in the trial, this information will be recorded on the study conclusion page, of the eCRF noting the date of withdrawal of consent, together with the reason for withdrawal (if the patient provides that information). The patient will be reassured that their future care will not be affected by their withdrawal, and that their relationship with their responsible physician remains unaltered. The patient will be informed that any of their study data already stored will be retained, and will be used, if necessary, in the evaluation of the study drugs and for adherence to the relevant regulatory requirements.

Patients may withdraw consent for the pharmacogenetic research without affecting their participation in the study as a whole. A pharmacogenetic consent withdrawal form will be completed and signed by the patient. It will record confirmation that the sample has been destroyed.

15.0 Enrolment

Before enrolment onto the study, the investigator will confirm that the patient meets all of the study inclusion criteria and none of the study exclusion criteria. Enrolment and allocation of patient ID will be carried out electronically via the eCRF and this system will prevent patients from being enrolling if they do not meet these inclusion/exclusion criteria requirements. This Patient ID will allow clinician to provide patient with order of procedures. The Patient ID acts as an identifier for return visits.

15.1 Violation of Eligibility criteria

In most cases, violation of eligibility criteria is not a reason for premature withdrawal from the study. However, if, post enrolment, it is found that the patient has not been eligible at the time this must be notified to the trial CI/co-ordinator as soon as possible, and, after detailed discussion regarding the particular patient, a decision will be taken about whether they should be withdrawn from the trial.

16.0 Trial Procedures

Home blood pressure measurements

At several points during the trial, home blood pressure measurements are recorded. These are to be measured seated after 5 minutes rest, using the non-dominant arm. Measurements will be made using an approved and validated monitor (Microlite watch BP) or equivalent. Blood pressure readings will be taken in the morning and the evening, and the timing of these should be consistent throughout the study, ideally ~ 8.0 am and 8.0 pm – or the nearest time to these that can be constant and convenient in the individual subject on 4 consecutive days. Participants will be asked to make triplicate seated readings after 5 minutes seated rest, and to record these on a pro-forma provided. All readings will also be captured automatically by the monitor.

16.1 Screening and enrolment visit

Informed Consent will be taken by physician or delegated to other members of the clinical research team. This will be taken following at least 24hrs of being in receipt of the patient information sheet. Demographic information will be recorded including gender, ethnicity, age, smoker, non-smoker, height weight. Medical history of cardiac, metabolic diseases and anything PI feels is related to hyperaldosteronism. Clinical examination. Concomitant hypertension medications will be recorded via class with a start and stop date where available.

During this visit the patient will have 3 blood pressures measured in the clinic following 5 minutes seated rest. Patients will be provided with a validated blood pressure monitor to measure home blood pressure. They will receive training on how to measure and record home blood pressures in blood pressure diary provided by a member of the clinical research team. This monitor will be used for clinic and home blood pressure measurements (Please see section above on Home blood pressure measurements)

A 12 lead electrocardiogram will be recorded. Followed by blood tests including electrolytes, , bicarbonate, creatinine, high troponin sensitivity test, brain natriuretic peptide (BNP), renin mass where available, renin activity, aldosterone and FBC. A serum HCG will be measured in child bearing potential females. Patients will be provided with x 1 24hr urine bottle for electrolytes which will be brought to baseline visit and a 10ml sample will be aliquoted for metabolomic analyses.

Inclusion and exclusion criteria will be checked with patient and recorded onto the eCRF. (Please see inclusion and exclusion criteria section 5.6)

Information and appointment details for 11C-metomidate PET CT scan and Adrenal Vein Procedures will be provided at this visit. The patient will then attend the PET CT scan and adrenal vein sampling procedures before next study visit , baseline visit.

NB Patients following review of PET CT scan and Adrenal Vein sampling procedure **who do not** meet the criteria for surgical intervention – adrenalectomy will attend for baseline visit, the post 2-week spironolactone /eplerenone50mg and the post 2 week spironolactone 100mg visit. Following on from this they will have a follow-up study visit at 9-12 months. During this visit clinic blood pressures, 12 lead ECG,

Prior to baseline visit

Home blood pressure measurements will be completed by the patient with 6 measurements for 4 days recorded in blood pressure diary and brought with them for the baseline visit. Patients will also be asked to attend a cardiovascular magnetic resonance (CMR) scan before or at baseline visit; this will require a washout period of 6 weeks for spironolactone/eplerenone and amiloride. The CMR will be repeated again at the 6 month visit +or- 1month. Patients not having surgical removal of an adrenal gland will have a follow- up CMR 9-12 months following their baseline visit. Patients will be asked to arrive approximately 30 minutes before the actual scan appointment time to perform a brief assessment, complete paper work and change into a gown. A small cannula will be inserted into the patient's vein, this allows a blood sample to be taken and administration of gadolinium; it is very widely used and usually has no side effects at all; very occasionally (less than 1/100 times) it causes a temporary mild headache or nausea. The scan itself takes about forty minutes. Patients will not be included if there are any contraindications to MR imaging such as cardiac pacemakers. Patients with a contraindication to gadolinium (i.e. GFR <30 ml/min/1.73m^2). No female patients will be scanned if pregnant.

16.2 Baseline Visit

During this visit the patient will have 3 blood pressures with heart rates measured in the clinic following 5 minutes seated rest. Home blood pressures taken from diary will be recorded onto eCRF

Bloods will be taken for electrolytes, bicarbonate, uric acid, creatinine, eGFR, high troponin sensitivity test, brain natriuretic peptide (BNP), renin mass, renin activity, aldosterone and serum magnesium. A serum HCG will be measured in child bearing potential females, 24 hours urine steroid profile, pharmacogenetics sample, plasma and serum samples, 24 hr Urine collection for sodium and potassium

Inclusion and exclusion criteria will be rechecked with any updates on concomitant medication.

During this visit the patient will be prescribed spironolactone/eplerenone 50mg once daily and have two return study visit at weeks 2 and 4 as below.

The aim is to have no longer than a 2 month run in period from screening visit to Baseline visit it will be up to PI interpretation and dependent on when 11 C Metomidate PET CT scan and AVS appointments. If a longer run in period is expected the CI should be consulted.

16.3 Spironolactone/Eplerenone Therapy visit (Baseline Visit)

(50mg) (post 2 week baseline visit)

- Clinic BP will be measured similarly to home readings (using the same monitor) - in triplicate, after 5 minutes seated rest
- Updated medical history
- Concomitant medications
- Home blood pressure recordings for 4 days (as above)
- Electrolytes and renal function

Spironolactone/Eplerenone (100mg) Visit week 4

- Clinic BP will be measured similarly to home readings (using the same monitor) - in triplicate, after 5 minutes seated rest
- Updated medical history
- Concomitant medications
- Home blood pressure recordings for 4 days (as above)
- Electrolytes and renal function.
- Following up titration patient will have recheck of electrolytes and renal function either at clinic or as arranged with GP.

16.4 Post-surgical and non-surgical follow up schedule visits at 1, 3,6,12 and 24 months.

Patients having adrenalectomy will be followed up at 1, 3, 6, 12 and 24 months. Final follow up visit will be up to two years following adrenalectomy. During these visits patients will have 3 blood pressures with heart rates measured in the clinic following 5 minutes seated rest. Home

blood pressures taken from diary will be recorded onto eCRF. Bloods will be taken for electrolytes, bicarbonate, creatinine, high troponin sensitivity test, brain natriuretic peptide (BNP), renin mass, renin activity, aldosterone and magnesium. Renin mass done at baseline and repeated post 6 months for adrenalectomy cohort and 6-9 months for aldosterone inhibitor medication cohort. (Please refer to schedule of events table). A serum HCG will be measured in child bearing potential females. Patients having a baseline CMR in this group will have a repeat at 6 -12 months following baseline CMR

For patients not requiring an adrenalectomy there will be at least one follow up visit at 9-12 following MDT. Patients having CMR in this group at baseline will have a repeat CMR at 6-12 months following MDT. Those recruited sufficiently early in the study, will have a second follow-up visit at about 2 years following MDT.

Patients from the adrenalectomy cohort will have a repeat or 24hr urine collection for metabolomics.

16.5 Unscheduled Visits

There will be provision in the eCRF for extra visits when considered necessary by the study team. This will not be considered a deviation of the protocol if these are not completed.

16.6 Premature termination of therapy or follow up and replacement of withdrawals.

Patients will be **withdrawn** from the study on safety grounds if any of the following occurs and at PI discretion:

- <u>Illness requiring hospitalisation that is likely to preclude further study participation</u>

- <u>Sustained Hypertension</u>
  Withdrawal will be up to PI discretion.

- <u>Hypotension</u>:
  If the patient has symptoms consistent with hypotension and systolic BP is <110mmHg or the change in BP from previous reading is >30mmHg, and in the opinion of the investigator there is no self-limiting reason for hypotension (e.g. episode of dehydration, climatic conditions) the patient will withdraw……

All study withdrawals will be recorded in the study conclusion page of the eCRF, indicating the date and reason for withdrawal. The sample size calculation allows for a small (10%) withdrawal rate, but patients will where possible be replaced until 149 patients have completed the study.

NB: It will be expected that patients return their blood pressure monitors at the end of the study or at withdrawal.

16.7 Plan of Further Treatment

Patients will be managed by their clinical physician.

17.0 Adverse events (AE/SAE)

Definition of Adverse Event
adverse event (ae) – any unfavourable and unintended sign, symptom or disease temporally associated with participation in the research project.
Definition of Serious Adverse Event
Serious adverse event (SAE) - an untoward occurrence that:
a. results in death
b. is life threatening
c. requires hospitalisation or prolongation of existing hospitalisation and. results in persistent or significant disability or incapacity
e. consists of a congenital anomaly or birth defect
f. is otherwise considered medically significant by the investigator
Recording and reporting Serious Adverse events

AEs will be identified by observation and /or enquiry at study visits. AEs that do not meet criteria for seriousness will be recorded in the medical notes only. Details of SAEs will be reported to the pharmacovigilance office at Queen Mary University of London (sponsor) using the QMUL Non CTIMP SAE form. Once aware of a SAE, scan and email/fax signed SAE form to the Research Governance & GCP Manager: **020 7882 7276 making sure subject number and site are added to form or email** research.safety@bartshealth.nhs.uk copying in MATCH trial coordinator via email.

Please send all unexpected and related SAEs to the QMUL sponsor immediately. SAEs will be followed until resolution. The relationship with the study procedures will be assessed for all SAEs. All ‘ study related’ SAEs will be forwarded to the CI and PI for assessment of the expectedness. All 'possibly' or 'definitely' related, unexpected SAEs will be reported to the REC on annual report.

All SAEs related to the study will be reported by the principal investigator (or designee) to sponsor as soon as reasonably practicable and in any event within 24 hours of first becoming aware of the event. Any follow up information should also be reported.

The SAE will need to be recorded onto MATCH eCRF in SAE section also for CI/PI review. All SAEs will be reviewed at TSC.

17.1 Reporting to research ethics committee

Any SAE occurring to a research participant will be reported to the main Rec (i.e. the rec that gave a favourable opinion of the study) where in the opinion of the chief investigator (CI), the event was:

• "related" – that is, it resulted from administration of any of the research procedures, and
• "unexpected" – that is, the type of event is not listed in the protocol as an expected occurrence.

Reports of related and unexpected SAEs should be submitted to the rec within 15 days of CI becoming aware of the event, using the 'report of serious adverse event form' for Non-CTIMPS published on the national research ethics service (nres) website. http://www.nres.npsa.nhs.uk/applications/after-ethical-review/safetyreports/safety-reports-for-all-other-research/ the form should be completed in typescript and signed by the CI (or designee). The sponsor's office will assist in the preparation and submission of the report. to the main rec who will acknowledge receipt of safety reports within 30 days.

17.2 Annual progress report

The CI or designee is also responsible for providing an annual progress report to the rec using an nres "annual progress report form for all other research". this form is available at: **http://www.nres.npsa.nhs.uk/applications/after-ethical-review/annual-progress-reports/** a section on the safety of participants is included in this report. The QMUL office will assist in the collation of the safety information required for the report. Reporting to local research and development (R&D) departments the principal investigator at each site is responsible for the provision of reports to their local R&D department per the conditions of management approval.

18.0 Laboratory aspect of the research

All blood biomarkers will be managed and reported at specific study sites were possible following their SOPs and guidelines in accredited biochemistry and haematology laboratories Guys and St Thomas's NHS Trust London, Addenbrookes Cambridge University Hospital, Cambridge and Barts Hospital NHS Trust, London and William Harvey research institute charterhouse square, London.

The 24 -hour urine steroid profile will be assayed and reported by the University of Birmingham as agreed by Sponsor's Technical Agreement devised by the sponsor. These will be transferred for analysis using accredited Human tissue transfer courier.

Plasma samples will require 2mls whole blood collected in EDTA spun at 300xG (RCF) and then aliquoted into 1.5ml Eppendorf tubes then frozen at - 80°C. For serum sample, 3mls of blood will be drawn and when clotted the serum will be aliquoted into 1.5 Eppendorf tubes and frozen -80°C. Both Plasma and serum samples will be transferred to Queen Mary University of London at the end of the study following the Sponsors Technical Agreement.

Following adrenalectomy tissue will be genotyped for somatic mutations in *KCNJ5, ATP1A1, ATP2B3, CACNA1D, CTNNB1* and phenotyping by histological grading, gene expression and immunohistochemistry. Routine analyses (histology, some of the immunohistochemistry) will

be performed by the endocrine pathologist in the hospital where the patient has surgery, and photographs retained for central, blinded re-analysis by the study endpoints committee. If the tests are not available in the hospital where surgery was performed, tissue will be transferred to either Addenbrookes Hospital Cambridge, or to Barts Hospital, or Queen Mary University London. Non-routine analyses (e.g. genotyping, gene expression) will be performed at William Harvey Research Institute QMUL Charterhouse Square London. Using fresh-frozen, RNAlater or paraffin section prepared at the time of surgery and stored at the local hospital until batched transfer to the site of analysis.

19.0 Statistical Aspect of the research

Patients will receive AVS and ^{11}C metomidate PET CT in ‘pseudo-random’ order. In order to allow for patients and situations where it is logistically easier to perform one investigation before the other – e.g. when the 11C-metomidate synthesis fails, and cannot be re-booked before an already scheduled AVS – we will use a minimization rather than randomisation procedure. This is a computer based programme which assigns order to each patient such that the difference in number of patients having one investigation first is minimized, but allows for the recommended order to be over-ridden. When eligibility has been confirmed, the order in which the diagnostic tests are to be performed will be obtained via the study web portal. The minimisation schedule will be stratified by study site. The minimisation schedule will be stored in a secure area of the Robertson Centre for Biostatistics network, with access restricted to those responsible for the randomisation system. Researchers at study sites will not know the order of tests until after baseline assessments have been carried out, and statisticians at the Robertson Centre will not have access to the minimisation schedule until the end of the study, when all analysis programs have been written and verified.

20.0 Study Outcomes

20.1 Primary Outcome(s)

1. Change in ARR from baseline to 6 months after surgery, or 9-12 months after MDT in patients not proceeding to surgery. (For patients whose surgery takes place less than six months prior to overall study-end, data will be used from the 3 month post-op visit, or the six-month visit will be brought forward. This qualification applies to all other reference to the six-month visit and data in this protocol.)
2. Change in SBP (mean of at least 12measurements from 3 days home monitoring), from baseline to 6 months after treatment, or 9-12 months after MDT in patients not proceeding to surgery

20.2 Secondary Outcomes

A number of pre-specified pre-operative radiological parameters, and post-operative histological, immunohistochemical, molecular and genetic measurements will be compared between patients

whose [i] ARR and [ii] blood pressure are normalized by surgical or medical treatment.

1. Cure of hypertension, defined as the proportion of patients in whom average home blood Pressure measurements 6 months post adrenalectomy are <140/90 mmHg on no treatment.

2. Prediction of cure of hypertension, by one or more of:
a) The ratio of SUVmax between tumour and normal adrenal;
b) Blood pressure fall on spironolactone/eplerenone (50 mg daily for 2 weeks, 100 mg daily for 2
weeks), measured as the difference in home systolic blood pressure
c) Post-operative analyses of the APA, to include genotyping for somatic mutations in KCNJ5, CACNA1D, ATP1A1, ATP2B3, CTNNB1 and phenotyping by histological grading (zona fasciculata versus zona glomerulosa), gene expression and immunohistochemistry.
3. Change in home blood pressure from baseline at follow-up visits other than 6 months post-adrenalectomy.
4. Reduction in number of antihypertensive drugs
5. Change from baseline in blood levels of Troponin, Brain Natriuretic Peptide.
6. Cardiac MRI changes in heart structure, anatomy and blood flow from baseline to six months.
7. Changes in Quality of life from baseline to six months.
8 Change in baseline serum cortisol and salivary cortisol measurements at 3 months post adrenalectomy.

The judgement as to which patients have met primary and secondary outcomes will be made by a critical endpoints committee meeting every 3-6 months throughout the study.

21.0 Planned Methods of Analysis

The planned study analyses will be detailed in the study-specific Statistical Analysis Plan (SAP), which will be finalised in advance of study closure.

The primary outcomes will be analysed in a hierarchical manner:

1. Change in ARR, between baseline and 6 months post-surgery (or equivalent time-point relative to baseline if no surgery), comparing patients who have surgery with patients who do not have surgery.

2. Blood pressure cure rates (home SBP < 135 mmHg on no antihypertensive treatment), comparing patients who have surgery with patients who do not have surgery.

3. ARR in patients who have surgery but have discordant AVS and PET-CT results, comparing patients whose surgery was indicated by high probability PET CT with patients whose surgery was indicated by high-probability AVS.

4. Blood pressure cure-or-control rates (home SBP =20 mm Hg or DBP =10 mm Hg lower than prespironolactone/eplerenone, i.e. baseline) in patients with discordant AVS and PET-CT results, comparing those whose surgery was indicated by high-probability PET CT with patients whose surgery was indicated by high-probability AVS.

5. Blood pressure cure rates (home SBP < 135 mmHg on no antihypertensive treatment) in patients with discordant AVS and PET-CT results, comparing those whose surgery was indicated by high-probability PET CT with patients whose surgery was indicated by high-probability AVS.

22.0 Safety Analysis.

Safety evaluation will include all patients in the Safety Population, and will be descriptive in nature.

22.1 Interim Analysis

There are no planned interim analyses.

22.2 Final Analysis

The final study analysis, as detailed in the SAP will be carried out after study closure and the study databases have been finalised and locked.

23.0 Sample Calculation

The calculations on sample size are based on our stated intention that AVS will only be performed in centres whose robust audit data indicate a bilateral cannulation rate of ≥75%, as the testing of the hypothesis will have maximum clinical value if ^{11}C metomidate scanning is compared with current best practice AVS. It also assumes that 100% of metomidate scans will provide a technically satisfactory result (we consider this to be reasonable, based on the Cambridge experience since the investigation was first introduced in May 2009).

The design of the study (and therefore the statistical analysis) presupposes that there will be a group of patients for whom the results from metomidate scanning and AVS will differ, but that the former is correct more often and that this difference is statistically significant. Given the results of the proof-of-concept study, it is inevitably the case that there will be a large group of patients for whom the two tests are concordant. The different scenarios of concordant/discordant, unilateral/bilateral, together with their estimated frequencies, are shown in Appendix, and it is these figures that have been used by the Clinical Trials Unit in Glasgow as the model for the sample size calculation and proposed analysis.

Recruitment of 165 patients across 3 centres over 3 years (<1/centre/month), with an estimated 70 proceeding to adrenalectomy, permits 90% power at alpha=0.01 of detecting non inferiority of PET CT in relation to AVS within a margin 16.7%. Based on the current size of referral practices at each centre, we are confident of meeting the recruitment target.

There will be a sub-study of 6 patients who will have a 11-C metomidate PET CT scan before and following spironolactone/eplerenone therapy. In our previous study, the uptake of metomidate into tumour was 60% greater than contralateral normal adrenal (mean(SD) = 21.7(8.0) vs 13.8 (3.0)). The study of six patients on two occasions will give us 90% power to detect just 30% increase in normal adrenal uptake, even if there is no parallel increase in tumour uptake of metomidate (alpha=0.05, one-sample test).

24.0 Ethical, Legal and Administrative Aspects

24.1 Ethics Committee

The study can only start after full and written approval of the protocol and all addenda has been obtained from the local Independent Ethics Committee. The Independent Ethics Committee must be informed of all subsequent, substantial protocol amendments issued by the sponsor. The investigator will provide reports to the Ethics Committee at intervals stipulated in the Ethics Committee guidelines. The MATCH study has been reviewed by the Dulwich Research Ethics Committee.

The study as detailed within this research protocol **(Version 4.0 dated 19 Nov 2019)**, or any subsequent amendments will be conducted in accordance with the UK Policy Framework for Health and Social Care Research, the World Medical Association Declaration of Helsinki (1996) and the current applicable regulatory requirements and any subsequent amendments of the appropriate regulations"

24.2 Data Monitoring and Ethics Committee (DMEC):

A DMEC is not planned for this study as the Trial Steering Committee consider that this Observational non-interventional study is at low risk of identifying any new unexpected events. All patients are receiving current standard clinical care, and the metomidate PET CT is non-invasive, and has been previously performed, without serious adverse events, in >300 patients during the last 10 years. All SAE's will be reported to the Trial Steering Committee.

24.3 Critical Endpoints Committee

This will consist at least one physician (Endocrinologist and/or Hypertension specialist), at least one Pathologist and a PET Nuclear Medicine Specialist. They will be independent of those involved in the patients' care, including the pre-operative and post-operative assessments. They will assess the pre-operative investigations (AVS and PET CT) without knowledge of outcome from surgery, and vice versa. This committee will meet every 3-6 months.

24.4 The Steering Committee

Trial Steering Committee (TSC): is chaired by Sir Christopher Edwards and includes three other independent members, Professor Bryan Williams, Professor Stephen Ball. The three lead investigators Prof Brown, Prof Drake and Dr Gurnell are also members. Observers from the EME Programme are invited to attend in person or by teleconference. A patients' representative has been identified and will be included. The TSC will aim to meet twice yearly.

25.0 Data Handling

The Robertson Centre for Biostatistics at Glasgow University will act as the Data Centre for the trial. It will be responsible for the development of a web-based electronic case report form (eCRF) system for recording trial data at the study sites.

The eCRF will include sections relating to each trial contact with the patient.

25.1 Data Capture

Study data will be is recorded via remote data entry into a web-based electronic case report form (eCRF) developed for the study. A manual for eCRF use will be provided for all trial sites. Data entry into the eCRF will be performed either by the investigator or documented designee. The investigator will confirm electronically the completeness and correctness of eCRF completion.

A reason will be supplied for any subsequent changes made to submitted eCRF data.

25.2 Data Collection and eCRF

A Patient Identification List will be created containing personal data such as the name, gender and date of birth of all study subjects, together with their assigned study number. This list does not form part of the eCRF, and will be stored securely at the study site. This is to enable later identification of the study subjects. However, the eCRF data will be anonymous, identifying study subjects by only their assigned study number.

Section 6 details the various data recorded during the course of the trial. These data will all be entered directly into the eCRF, with the exception of the following, which will be recorded electronically and transmitted separately to the Data Centre.

- Baseline visit 12-lead ECG result

25.3 Data Management.

Whenever possible, data will be entered into the eCRF directly online. If this is not possible, data will be entered and stored in password protected shared area. Where USB sticks are used to store information they will need to be encrypted and stored by CI delegated MATCH researcher. All information electrocardiograms etc will be stored by subject number only. At the end of the study all data will be archived according to sponsor SOP for up to 20 years.

The Robertson Centre for Biostatistics, University of Glasgow is responsible for all data management for the trial data. Data management will be in accordance with the Robertson Centre's SOPs.

25.4 Record Retention

ICH/GCP guidelines require that the investigator or the institution maintains all Case Report Forms and all source documents that support the data collected from each participant plus all trial documentation. Measures will be taken to prevent accidental or premature destruction of these documents. Essential documents must be retained for at least 20 years.. It is the responsibility of the sponsor to inform the investigator when these documents no longer need to be retained. If the responsible investigator retires or leaves the institution responsibility for the documentation must be transferred to a person who will accept their custody.

25.5 Record Archival

The study data and documentation will be archived in accordance with the relevant regulatory requirements and site SOPs.

26.0 Quality Control and Assurance

This clinical study will be conducted in compliance with the protocol, GCP and the applicable regulatory requirements.

26.1 Direct Access to Source Data and Documents

According to GCP, the trial investigators will permit authorised third parties access to the trial site and medical records relating to trial subjects. This will include, but not necessarily be restricted to, access for trial-related monitoring, audits, Ethics Committee review and regulatory inspections.

26.2 Monitoring

Monitoring of centre may be undertaken by a trained study monitor or monitors, to verify the completeness and correctness of the study documentation.

26.3 Audits

The sponsor reserves the right to audit trial sites, subject to giving a suitable period of notice (minimum period of notice 10 working days).

26.4 Inspections

Any study site may be audited by the relevant legal/ regulatory authorities.

27.0 Data Protection

Study data will be protected in accordance with the UK data protection legislation. All study data held electronically from participating study sites will be anonymised. Data will only be identified by the subjects unique study identifier.

The Patient Identification List (linking study number to patient identity) will be stored securely at the study site.

27.1 Administrative agreements and protocol adherence

This observational study will be conducted in accordance with the declaration of Helsinki including all revisions.

Protocol violations comprise any deviation from the procedures defined in the protocol, any missed assessments, and non-compliance with study medication. The investigator should make considerable effort to ensure protocol adherence, or to correct any protocol violations that occur.

All protocol violations should be documented, and any major protocol violations should be reported immediately to the study co-ordinator and Chief Investigator.

If a protocol deviation is required in order to eliminate an immediate hazard to a study participant, this should be documented, indicating the form of deviation, and the reason that this was necessary.

27.2 Protocol Amendments

Neither the investigators nor any personnel involved in the study conduct may alter the study protocol. Protocol amendments will only be made under exceptional circumstances, and only with full agreement of the study Trial Steering Committee. Any protocol amendments agreed will be recorded in writing. All protocol amendments will be notified to all study sites and involved study personnel.

28.0 Funding

The MATCH study is supported by funding from the NIHR EME project number **EME Project number: 14/145/09.**

28.1 Insurance

Queen Mary University of London is sponsoring this study and has arranged for suitable indemnity cover to be in place. Cambridge University as the manufacturer of11-C metomidate will insure the product. Cambridge University Hospitals NHS Foundation Trust at which the 11-C metomidate PET CT scan is to be undertaken will provide suitable indemnity cover for the procedure.

28.2 Publication Policy

Publications will be supervised by the British Hypertension Society Research Committee. This committee will determine the authorship of each paper using CONSORT guidelines.

28.3 Case Payments

Investigators will only be reimbursed for any study-related expenses.

29.0 CONFIRMATION OF THE FINAL PROTOCOL

The signatories confirm that they agree to conduct their study-related responsibilities in accordance with local law, the declaration of Helsinki including all revisions, and the study protocol

Chief investigator
(Professor M J Brown) Signature__________________ Date_________

29.1 PROTOCOL AGREEMENT

The signatory declares that:

- he/she will conduct his/her study responsibilities in accordance with law, the declaration of Helsinki including all revisions, and the study protocol.

- he/she has read the study protocol and agrees to it in its entirety

Principal investigator

NAME (block capitals): ________________________________

Signature: ________________________________

Date: ________________________________

Affiliation and address: ________________________________

____

____

____

____

30.0 APPENDICES

Abbreviations

AE	Adverse event
ACTH	Adrenal
ARB	Angiotensin receptor blocker
ARR	Aldosterone renin ratio
ACEI	Angiotensin converting enzyme inhibitor
AVS	Adrenal Vein Sampling
BAH	Bilateral adrenal Hyperplasia
BP	Blood pressure
BHS	British Hypertension Society
BHF	British Heart Foundation
CETO [18F]	
CCB	Calcium Channel Blocker
eCRF	electronic Case Report/Record Form
DBP	Diastolic blood pressure
ECG	Electrocardiogram
HCG	Human chorionic gonadotrophin
ICH	International Conference on Harmonization
IEC	Independent Ethics Committee
IRB	Institutional Review Board
IVRS	Interactive voice response system
IV	Intravenous
MDT	Multidisciplinary Team
MR	Mineralocorticoid receptor
NICE	National Institute for Clinical Excellence
o.d.	Omnia die/once a day
PI	Principal Investigator
PET	Positron electron tomography
PH	Primary hyperaldosteronism
PHA	Primary Hyperaldosteronism
p.o.	Per os/by mouth/orally
SAE	Serious adverse event
SBP	Systolic blood pressure
SUSAR	Suspected unexpected serious adverse drug reaction

31.0 PHARMACOGENETIC RESEARCH

DNA repositories from studies so patients may gain early benefits from the rapid advances in DNA technology in tailoring therapy or reducing side effects. It is already possible to study more than a million markers or single nucleotide polymorphisms across the genome. By the time our study is finished, it may be financially as well as technically feasible to sequence every gene in the genome.

32.0 Study Population and Assessment

Prior to subject participation, specific informed consent for this procedure will be signed by the patient. This research is voluntary and if a patient refuses pharmacogenetic participation they will not be excluded from the clinical study.
A 10ml sample of whole blood will be taken into an EDTA tube following informed consent. Although it is best to take the sample at the beginning of the study, it may be taken any time throughout the study. The tube label will identify subjects only by study number. No personal details will be recorded on the tube, and only designated study members will be able to link DNA analyses to the anonymised clinical data collected during the study. Samples will be stored securely at study sites before shipment to St Bartholomew's London and other sites will be invited during progress of study if required and ethics will be updated .

The named study team members at each site will hold a link between study number and patient name, as is required to permit monitoring and audit of patient records. This link will be used if a subject wishes to withdraw their sample from analysis, subjects can request destruction of their sample and any data extracted from it and pharmacogenetic consent withdrawal form will be completed and signed by the patient. It will record confirmation that the sample has been destroyed following site destruction Trust policy..

Pharmacogenetic research is likely to continue following completion of the clinical study. As is standard for most large-scale genetic studies, samples will be held indefinitely in view of the large amount of potential data they contain and the length of time it might take to mine this data. As the potential uses of these samples cannot be fully predicted at this point, the Informed Consent Document stipulates that samples might also be used for research purposes unrelated to the focus of this clinical study.
If a subject withdraws from the study other than not attending follow up and has not asked for pharmacogenetic destruction, research will continue as stated in their informed consent.

33.0 CALCULATION OF PROPOSED SAMPLE SIZE

The table below shows potential outcomes from the two separate investigations in patients recruited to MATCH:

**Diagnostic Tests**		**Normalisation of plasma renin & aldosterone/renin ratio after surgery (i.e. confirmed unilateral PHA)**	
**AVS**	**PET-CT**	**No**	**Yes**
Unsuccessful	Bilateral	A (=9%)	B (=0%)
Unsuccessful	Unilateral	C (=1%)	D (=10%)
Bilateral	Bilateral	E (=35%)	F (=0%)
Bilateral	Unilateral	G (=1%)	H (=9%)
Unilateral	Bilateral	I (=0.5%)	J (=3.5%)
Unilateral (c)	Unilateral	K (=1%)	L (=29%)
Unilateral (d)	Unilateral	M (=0.25%)	O (=0.25%)
Unilateral	Unilateral (d)	P (=0.25%)	Q (=0.25%)

Key: c = concordant; D = discordant (i.e. PET-CT and AVS lateralise to opposite sides)

The accuracy of PET CT will be estimated in patients having surgery, given by:
(D+H+I+L+O+Q) / n
The accuracy of AVS will be estimated by (G+J+L+M+P) / n
A test of whether these accuracies differ will therefore be a test of whether
(D+H+I+L+O+Q) = (G+J+L+M+P)
or whether
(D+H+I+O+Q) = (G+J+M+P)
This can be achieved by a one sample binomial test of whether
p = (D+H+I+O+Q) / (D+H+I+G+J+M+O+P+Q) = 50%

Given the estimated proportions given above, the anticipated value of p is 80%. To have 90% power at a 1% significance level to show that p is different to 50%, assuming the true value of p to be 80%, we require the number of participants in the subgroup (A+D+H+I+G+J+M+O+P+Q) to be 32 (based on an exact binomial test). Since this subgroup is an estimated 25% of the study population, we would need to recruit 128 participants
Assuming missing data of 10% of people recruited, our target recruitment will be 140. This original sample size calculation assumed that 50% of patients would proceed to medical therapy, and 50% to surgery. In the light of an observed asymmetry between these two group sizes, the total sample will be increased to 165."

34.0 References

1. Rossi GP, Bernini G, Caliumi C, Desideri G, Fabris B, Ferri C, Ganzaroli C, Giacchetti G, Letizia C, Maccario M, Mallamaci F, Mannelli M, Mattarello MJ, Moretti A, Palumbo G, Parenti G, Porteri E, Semplicini A, Rizzoni D, Rossi E, Boscaro M, Pessina AC, Mantero F. A prospective study of the prevalence of primary aldosteronism in 1,125 hypertensive patients. J Am Coll Cardiol 2006; 48:2293-2300
2. Mulatero P, Stowasser M, Loh KC, Fardella CE, Gordon RD, Mosso L, Gomez-Sanchez CE, Veglio F, Young WF, Jr. Increased diagnosis of primary aldosteronism, including surgically correctable forms, in centers from five continents. J Clin Endocrinol Metab 2004; 89:1045-1050
3. Stowasser M, Gordon RD, Gunasekera TG, Cowley DC, Ward G, Archibald C, Smithers BM. High rate of detection of primary aldosteronism, including surgically treatable forms, after 'non-selective' screening of hypertensive patients. J Hypertens 2003; 21:2149-2157
4. Rossi GP. A comprehensive review of the clinical aspects of primary aldosteronism. Nat Rev Endocrinol 2011; 7:485-495
5. Calhoun DA, Nishizaka MK, Zaman MA, Thakkar RB, Weissmann P. Hyperaldosteronism Among Black and White Subjects With Resistant Hypertension. Hypertension 2002; 40:892-896
6. Milliez P, Girerd X, Plouin PF, Blacher J, Safar ME, Mourad JJ. Evidence for an increased rate of cardiovascular events in patients with primary aldosteronism. J Am Coll Cardiol 2005; 45:1243-1248
7. Falaschetti E, Mindell J, Knott C, Poulter N. Hypertension management in England: a serial cross-sectional study from 1994 to 2011. Lancet 2014; 383:1912-1919
8. Funder JW. Primary aldosteronism: clinical lateralization and costs. J Clin Endocrinol Metab 2012; 97:3450-3452
9. Rutherford JC, Taylor WL, Stowasser M, Gordon RD. Success of surgery for primary aldosteronism judged by residual autonomous aldosterone production. World J Surg 1998; 22:1243-1245
10. Pang TC, Bambach C, Monaghan JC, Sidhu SB, Bune A, Delbridge LW, Sywak MS. Outcomes of laparoscopic adrenalectomy for hyperaldosteronism. ANZ J Surg 2007; 77:768-773
11. Lim V, Guo Q, Grant CS, Thompson GB, Richards ML, Farley DR, Young WF, Jr. Accuracy of adrenal imaging and adrenal venous sampling in predicting surgical cure of primary aldosteronism. J Clin Endocrinol Metab 2014; 99:2712-2719
12. Hood SJ, Picton DE, Taylor KP, Brown MJ. Value of routine plasma renin assay in the detection, treatment-titration or cure of low-renin patients with salt-dependent hypertension. Journal of Human Hypertension 2009; 23:693
13. Brown MJ. Clinical value of plasma renin estimation. Am J Hypertens 2014; 10.1093/ajh/hpu113
14. Lau JH, Sze WC, Reznek RH, Matson M, Sahdev A, Carpenter R, Berney DM, Akker SA, Chew SL, Grossman AB, Monson JP, Drake WM. A prospective evaluation of postural stimulation testing, computed tomography and adrenal vein sampling in the differential diagnosis of primary aldosteronism. Clin Endocrinol (Oxf) 2012; 76:182-188
15. Young WF, Jr. Clinical practice. The incidentally discovered adrenal mass. N Engl J Med 2007; 356:601-610

16. Rossi GP, Barisa M, Allolio B, Auchus RJ, Amar L, Cohen D, Degenhart C, Deinum J, Fischer E, Gordon R, Kickuth R, Kline G, Lacroix A, Magill S, Miotto D, Naruse M, Nishikawa T, Omura M, Pimenta E, Plouin PF, Quinkler M, Reincke M, Rossi E, Rump LC, Satoh F, Schultze Kool L, Seccia TM, Stowasser M, Tanabe A, Trerotola S, Vonend O, Widimsky J, Jr., Wu KD, Wu VC, Pessina AC. The Adrenal Vein Sampling International Study (AVIS) for identifying the major subtypes of primary aldosteronism. J Clin Endocrinol Metab 2012; 97:1606-1614
17. Rossi GP, Auchus RJ, Brown M, Lenders JW, Naruse M, Plouin PF, Satoh F, Young WF, Jr. An expert consensus statement on use of adrenal vein sampling for the subtyping of primary aldosteronism. Hypertension 2014; 63:151-160
18. Preda VA, Sen J, Karavitaki N, Grossman AB. Etomidate in the management of hypercortisolaemia in Cushing's syndrome: a review. Eur J Endocrinol 2012; 167:137-143
19. Soh LM, Gunganah K, Akker SA, Jones P, Khachi H, Dodzo K, Drake WM. Etomidate in the emergency management of hypercortisolemia. Eur J Endocrinol 2012; 167:727-728; author reply 729
20. Bergstrom M, Bonasera TA, Lu L, Bergstrom E, Backlin C, Juhlin C, Langstrom B. In vitro and in vivo primate evaluation of carbon-11-etomidate and carbon-11-metomidate as potential tracers for PET imaging of the adrenal cortex and its tumors. J Nucl Med 1998; 39:982-989
21. Bergstrom M, Juhlin C, Bonasera TA, Sundin A, Rastad J, Akerstrom G, Langstrom B. PET imaging of adrenal cortical tumors with the 11beta-hydroxylase tracer 11C-metomidate. J Nucl Med 2000; 41:275-282
22. Hennings J, Lindhe O, Bergstrom M, Langstrom B, Sundin A, Hellman P. [11C]metomidate positron emission tomography of adrenocortical tumors in correlation with histopathological findings. J Clin Endocrinol Metab 2006; 91:1410-1414
23. Powlson AS, Gurnell M, Brown MJ. Nuclear imaging in the diagnosis of primary aldosteronism. Curr Opin Endocrinol Diabetes Obes 2015; 22:DOI:10.1097/MED.0000000000000148
24. Burton TJ, Mackenzie IS, Balan K, Koo B, Bird N, Soloviev DV, Azizan EA, Aigbirhio F, Gurnell M, Brown MJ. Evaluation of the sensitivity and specificity of (11)C-metomidate positron emission tomography (PET)-CT for lateralizing aldosterone secretion by Conn's adenomas. J Clin Endocrinol Metab 2012; 97:100-109
25. Powlson AS, Kolouri O, Challis BG, Cheow HK, Buscombe J, Koo B, Brown MJ, Gurnell M. 11C-metomidate PET–CT in primary hyperaldosteronism: a valuable alternative to AVS. Endocrine Abstracts 2014; 34:365
26. Mulatero P, Bertello C, Sukor N, Gordon R, Rossato D, Daunt N, Leggett D, Mengozzi G, Veglio F, Stowasser M. Impact of different diagnostic criteria during adrenal vein sampling on reproducibility of subtype diagnosis in patients with primary aldosteronism. Hypertension 2010; 55:667-673
27. Azizan EA, Poulsen H, Tuluc P, Zhou J, Clausen MV, Lieb A, Maniero C, Garg S, Bochukova EG, Zhao W, Shaikh LH, Brighton CA, Teo AE, Davenport AP, Dekkers T, Tops B, Kusters B, Ceral J, Yeo GS, Neogi SG, McFarlane I, Rosenfeld N, Marass F, Hadfield J, Margas W, Chaggar K, Solar M, Deinum J, Dolphin AC, Farooqi IS, Striessnig J, Nissen P, Brown MJ. Somatic mutations in ATP1A1 and CACNA1D underlie a common subtype of adrenal hypertension. Nat Genet 2013; 45:1055-1060
28. Azizan EA, Lam B, Newhouse S, Zhou J, Clarke J, Happerfield L, Marker A, Hoffman G, Brown MJ. Microarray, qPCR and KCNJ5 sequencing of aldosterone-producing adenomas reveal differences in genotype and phenotype between zona glomerulosa- and zona fasciculata-like tumors. J Clin Endocrinol Metab 2012; 97:E819-829

29. Brown MJ. Ins and outs of aldosterone-producing adenomas of the adrenal: from channelopathy to common curable cause of hypertension. Hypertension 2014; 63:24-26

30. Forshew T, Murtaza M, Parkinson C, Gale D, Tsui DW, Kaper F, Dawson SJ, Piskorz AM, Jimenez-Linan M, Bentley D, Hadfield J, May AP, Caldas C, Brenton JD, Rosenfeld N. Noninvasive identification and monitoring of cancer mutations by targeted deep sequencing of plasma DNA. Science translational medicine 2012; 4:136ra168

31. Funder JW, Carey RM, Mantero F, Murad MH, Reincke M, Shibata H, Stowasser M, Young WF, Jr. The Management of Primary Aldosteronism: Case Detection, Diagnosis, and Treatment: An Endocrine Society Clinical Practice Guideline. J Clin Endocrinol Metab 2016:jc20154061

Is Metomidate PET-CT superior to Adrenal venous sampling in predicting outcome from adrenalectomy in patients with primary Hyperaldosteronism

(MATCH)

STATISTICAL ANALYSIS PLAN (SAP)

Study Title:	Is Metomidate PET-CT superior to Adrenal venous sampling in predicting ouTCome from adrenalectomy in patients with primary Hyperaldosteronism		
Short Title:	MATCH		
IDs:	Research Identification Number: 011149<br>ClinicalTrials.gov Identifier: NCT02945904<br>Funder ID: EME 14/145/09		
Sponsor:	Queen Mary University of London (QMUL)		
Funded by:	NIHR EME		
Protocol Version:	6.0	Date:	25/11/2020
SAP Version:	v1	Date:	22/02/2021

		Signature	Date
Written by:	Prof Alex McConnachie<br>Assistant Director of Biostatistics, Robertson Centre for Biostatistics, University of Glasgow		22/02/2021
	Alasdair McIntosh<br>Junior Biostatistician, Robertson Centre for Biostatistics, University of Glasgow		22/02/2021
Authorised by:	Michele Robertson<br>Assistant Director Commercial Biostatistics, Robertson Centre for Biostatistics, University of Glasgow		22/02/2021
	Professor M J Brown<br>Professor of Endocrine Hypertension, The Barts Heart Centre, William Harvey Research Institute, Queen Mary University of London, Charterhouse Square, London EC1M 6BQ		22/02/2021

CONTENTS

1. Introduction 3
1.1. Study Background 3
1.2. Study Objectives 3
1.3. Study Design 4
1.4. Randomisation 4
1.5. Sample Size and Power 4
1.6. Interim Analysis and Stopping Rules 5
1.7. Study Population 5
1.8. Outcomes 6
1.9. PASO Criteria 7
1.10. Statistical Analysis Plan (SAP) 9
2. Analysis Populations 11
2.1. Subject Disposition 11
2.2. Baseline Characteristics 11
2.3. Efficacy Outcomes 19
2.4. Safety Outcomes 21
3. Data Conventions 21
4. Document History 21
5. Tables 22
6. Figures 22
7. Listings 22

1. Introduction

1.1. Study Background

Hypertension is a major risk factor for heart attacks and stroke. Primary hyperaldosteronism (PHA) is an important cause of hypertension and is characterised by excessive aldosterone production by one or both adrenal glands. PHA carries significant cardiometabolic risk over and above that conferred by elevated blood pressure alone. Stroke, myocardial infarction and atrial fibrillation are increased 4 to 12 fold compared to age, gender and blood pressure-matched subjects with essential hypertension.

Detection rates of PHA have improved, largely due to greater availability of high throughput renin assays. However, in current UK clinical practice, only a tiny proportion of patients with PHA complete the investigational pathway and are referred for potentially curative surgery.

One of the main barriers is the difficulty of proving that only one adrenal gland is the culprit. The test widely considered to be most accurate in distinguishing unilateral (surgically-treated) from bilateral (managed medically) disease is adrenal venous sampling (AVS). This diagnostic procedure is difficult, time-consuming, invasive, available in only a few centres, and has variable success rates, depending on technical expertise and the diagnostic criteria employed.

A non-randomised proof-of-concept study showed that 11C-metomidate PET CT scanning is comparable to AVS in distinguishing unilateral from bilateral PHA. Since then, considerable clinical experience with 11C-metomidate PET-CT scanning has accumulated, although robust data are lacking to endorse its widespread use as the investigation of choice.

1.2. Study Objectives

1.2.1. Primary Objective

To determine whether 11C-metomidate PET-CT is superior to adrenal vein sampling in diagnosing unilateral PA, by measuring normalisation of aldosterone/renin ratio and potassium, and change in blood pressure, following adrenalectomy.

1.2.2. Secondary Objectives

The secondary objectives as listed in the protocol are:

- To investigate whether outcomes differ between those patients who progress to surgery on the basis of PET-CT and/or AVS results, and those patients who do not.

- To investigate whether measurements during 11C-metomidate PET-CT or AVS improve prediction of outcome after surgery.
- To investigate whether blood pressure response to therapy with spironolactone/eplerenone predicts outcome after surgery.
- To investigate whether demographic, genotypic, and pathological data, improve prediction of outcome after surgery.

1.2.3. Assessment of Efficacy and Safety

Efficacy will be determined from measurements of Aldosterone Renin Ratio (ARR), Potassium, Blood Pressure (BP), and BP medication, each at baseline and following treatment. Differences in outcome between those who undergo surgery on the basis of either 11C-metomidate PET-CT or AVS, but not both, will be used to compare performance.

Safety will be assessed by monitoring symptoms, BP, renal function and serious adverse events at each visit.

1.3. Study Design

This is an observational multi-centre prospective cohort study.

1.4. Randomisation

The order in which diagnostic tests were to be applied was randomised, but this was not always logistically feasible. Where applicable, data may be summarised in relation to the randomised order of the tests, or the order in which the tests were received.

1.5. Sample Size and Power

The original sample size calculations carried out at the design stage are reported in section 33.0 of the study protocol, and is reproduced below:

The table below shows potential outcomes from the two separate investigations in patients recruited to MATCH:

Diagnostic Tests		Normalisation of plasma renin & aldosterone/renin ratio after surgery (i.e. confirmed unilateral PHA)	
**AVS**	**PET-CT**	**No**	**Yes**
Unsuccessful	Bilateral	A (=9%)	B (=0%)
Unsuccessful	Unilateral	C (=1%)	D (=10%)
Bilateral	Bilateral	E (=35%)	F (=0%)
Bilateral	Unilateral	G (=1%)	H (=9%)
Unilateral	Bilateral	I (=0.5%)	J (=3.5%)
Unilateral (c)	Unilateral	K (=1%)	L (=29%)
Unilateral (d)	Unilateral	M (=0.25%)	O (=0.25%)
Unilateral	Unilateral (d)	P (=0.25%)	Q (=0.25%)

Key: c = concordant; D = discordant (i.e. PET-CT and AVS lateralise to opposite sides)

The accuracy of PET CT will be estimated in patients having surgery, given by:

(D+H+I+L+O+Q) / n

The accuracy of AVS will be estimated by (G+J+L+M+P) / n

A test of whether these accuracies differ will therefore be a test of whether

(D+H+I+L+O+Q) = (G+J+L+M+P)

or whether

(D+H+I+O+Q) = (G+J+M+P)

This can be achieved by a one sample binomial test of whether

p = (D+H+I+O+Q) / (D+H+I+G+J+M+O+P+Q) = 50%

Given the estimated proportions given above, the anticipated value of p is 80%. To have 90% power at a 1% significance level to show that p is different to 50%, assuming the true value of p to be 80%, we require the number of participants in the subgroup (A+D+H+I+G+J+M+O+P+Q) to be 32 (based on an exact binomial test). Since this subgroup is an estimated 25% of the study population, we would need to recruit 128 participants. Assuming missing data of 10% of people recruited, our target recruitment will be 140.

1.6. Interim Analysis and Stopping Rules

Not applicable.

1.7. Study Population

The full list of inclusion and exclusion criteria are given in the study protocol, section 10.0.

1.8. OUTCOMES

Outcomes for statistical analysis will be assessed at 6 months after surgery, or 9-12 months after MDT decision, in those who do not undergo surgery. These time points are considered equivalent, occurring at approximately the same time since baseline. Where follow-up data is not available at this time point, data from a later visit (up to 12 months) will be used; if this is not available, an earlier visit (closest available) will be used.

Outcomes for statistical analysis will be referred to as collected "at 6 months" throughout this SAP.

1.8.1. PRIMARY OUTCOME

The following outcomes will be considered as primary outcomes. These outcomes relate to cure of hypertension at 6 months. Definitions of biochemical and clinical success according to the PASO criteria will be used (see Section 1.9). These will be analysed hierarchically, with each analysis being considered as part of the primary analysis if all preceding analyses demonstrate statistical significance at $p<0.05$. Otherwise, analyses of subsequent outcomes will be considered to be secondary analyses.

- At least partial biochemical success
- Complete biochemical success
- At least partial clinical success
- Complete clinical success

1.8.2. SECONDARY OUTCOMES

The secondary outcomes are:

- Biochemical success (PASO criteria, Section 1.9) at 6 months, analysed as a 3-level categorical variable (complete, partial, none);
- Clinical success (PASO criteria, Section 1.9) at 6 months, analysed as a 3-level categorical variable (complete, partial, none);
- Modified clinical success (based on PASO criteria, Section 1.9) at 6 months, analysed as a 3-level categorical variable (complete, partial, none);
- Change in serum potassium from baseline to 6 months;
- Changes in $ARR_{activity}$ and ARR_{mass} from baseline to 6 months;
- Changes in home SBP and DBP from baseline to 6 months;
- Changes in clinic SBP and DBP from baseline to 6 months;

- Change in defined daily doses of antihypertensive drugs from baseline to 6 months;
- Change in number of antihypertensive drugs from baseline to 6 months;
- Change in number of classes of antihypertensive drugs from baseline to 6 months;
- Change in blood levels of Troponin, Brain Natriuretic Peptide from baseline to 6 months;
- Changes in cardiac MRI measures of heart structure, anatomy and blood flow from baseline to 6 months;
- Changes in quality of life from baseline to 6 months.

1.9. PASO CRITERIA

The following criteria will be used to define cure of hypertension, from the PASO criteria [Williams TA, et al. Lancet Diabetes Endocrinol. 2017 Sep; 5(9): 689-699], six months post-adrenalectomy.

Clinical success:

- Complete clinical success

  Complete clinical success will be declared when both (a) and (b) have occurred:

  (a) Normalisation of BP, defined as SBP<135 and DBP<85 mmHg on home BP; if home BP not available, then defined as SBP<140 and DBP<90 mmHg on clinic BP

  (b) Not taking any antihypertensive medications

- Partial clinical success

  Partial clinical success will be declared when the criteria for complete success are not met, and either (a) or (b) has occurred:

  (a) reduction in BP, with no increase in antihypertensive medication, i.e. both of (i) and (ii) have occurred:

  (i) BP has reduced, or has normalised, since baseline (based on home BP if available, otherwise based on clinic BP); reduction in BP defined as

  (1) a reduction in SBP of ≥20mmHg, or
  (2) a reduction in DBP of ≥10mmHg, with a change in SBP of <20mmHg

(ii) the defined daily dose of antihypertensive medication being used is <150% of baseline

(b) reduction in antihypertensive medication, with no increase in BP, i.e.

(i) BP is unchanged since baseline (based on home BP if available, otherwise based on clinic BP); unchanged BP defined as

(1) a change in SBP of <20mmHg, and
(2) a change in DBP of <10mmHg

(ii) the defined daily dose of antihypertensive medication being used is ≤50% of baseline

- Absent clinical success

  An absence of clinical success will be declared when neither partial nor complete clinical success can be declared.

Biochemical success:

- Complete biochemical success

  Complete biochemical success will be declared when both of the following occur:

  - Serum potassium ≥3.6 mmol/l
  - Normalisation of ARR ($ARR_{activity}$ <1000 pmol/L per nmol/L/hr, if renin activity is recorded, otherwise ARR_{mass} <91 mIU/L)

- Partial biochemical success

  Partial biochemical success will be declared when the criteria for complete success are not met, and both of the following occur:

  - Serum potassium ≥3.6 mmol/l
  - ≥50% reduction in serum aldosterone concentration

- Absent biochemical success

  An absence of biochemical success will be declared when neither partial nor complete clinical success can be declared.

In addition, a modified definition of clinical success (complete, partial, or absent) will be defined as follows:

Modified clinical success:

- Complete modified clinical success

  Complete modified clinical success will be declared when both (a) and (b) have occurred:

  (a) Normalisation of SBP, defined as SBP<135mmHg on home BP; if home BP not available, then defined as SBP<140mmHg on clinic BP

  (b) Not taking any antihypertensive medications

- Partial modified clinical success

  Partial modified clinical success will be declared when the criteria for complete success are not met, and either (a) or (b) has occurred:

  (a) reduction in SBP, with no increase in antihypertensive medication, i.e. both of (i) and (ii) have occurred:

  (i) SBP has reduced by ≥20mmHg, or has normalised, since baseline (based on home BP if available, otherwise based on clinic BP)

  (ii) the number of classes of antihypertensive medication being used is no more than 1 more than at baseline

  (b) reduction in antihypertensive medication, with no increase in SBP, i.e.

  (i) SBP is unchanged since baseline (based on home BP if available, otherwise based on clinic BP); unchanged SBP defined as a change in either direction of <20mmHg

  (ii) the number of classes of antihypertensive medication being used has reduced by 2 or more since baseline, or the use of antihypertensive medication has been stopped

- Absent modified clinical success

  An absence of modified clinical success will be declared when neither partial nor complete modified clinical success can be declared.

1.10. Statistical Analysis Plan (SAP)

1.10.1. SAP Objectives

The objective of this SAP is to describe the statistical analyses to be carried out for the MATCH Study.

1.10.2. General Principles

Continuous data will be summarised as the number of observations and number of missing values, mean, standard deviation, median, quartiles, and range. Categorical data will be summarised as the number of observations and number of missing values, frequencies, and percentages.

Continuous measures collected at baseline and follow-up will be summarised at each time point separately, and as changes from baseline. Missing follow-up values may be carried forward from earlier visits for selected analyses; in such instances, summary data will be presented both without, and with imputation.

Groups will be compared using appropriate independent samples tests, with results presented as estimated between-group difference (or other comparative statistic), with 95% confidence interval, and p-value. Regression-based methods will also be used, adjusting for baseline values where appropriate, and for other baseline characteristics. Details of adjustment variables will be reported as part of the statistical outputs.

1.10.3. Current Protocol

The current study protocol at the time of writing is version 6.0, dated 25/11/2020. Following discussions with the Chief Investigator, and with input from the Trial Steering Committee, the analyses outlined in this SAP differ from those in the protocol; it is planned that the protocol will be amended to reflect these changes.

1.10.4. Deviations To Those Specified In Study Protocol

This SAP does not cover the analyses of data relating to 18F-CETO PET CT. A separate plan will be developed for this part of the study.

The primary outcomes specified in this SAP relate to the PASO criteria for biochemical and clinical cure. These are similar to, but more scientifically relevant than the primary outcomes specified in the current protocol.

1.10.5. Additional Analyses To Those Specified In Study Protocol

No major additional analyses to those outlined in the protocol are planned.

1.10.6. Software

Data will be analysed using R Version 3.6.2 or later. The final report will be provided as a Word document.

2. ANALYSIS POPULATIONS

The "Enrolled Population" (EP) will consist of those who gave consent and met all inclusion/exclusion criteria.

The "Full Analysis Set" (FAS) will consist of those members of the EP who underwent both diagnostic tests (AVS and PET CT).

The "Substudy Population" (SP) will consist of those members of the EP who had one PET scan on spironolactone and one PET scan off spironolactone.

The numbers in each population will be reported. Reasons for exclusion from the FAS will be summarised. For those in the FAS, the numbers of participants who were randomised to each scan order, and who received the scans in each order, will be reported.

2.1. SUBJECT DISPOSITION

For those in the FAS, the number and percentage of participants who completed each study visit will be reported.

2.2. BASELINE CHARACTERISTICS

2.2.1. PARTICIPANT CHARACTERISTICS AT SCREENING

The following information collected at screening will be summarised for all participants in the Enrolled Population, in the Full Analysis Set, and in the Substudy Population; for those in the FAS, data will be presented according to whether the participant later underwent surgical treatment:

- Age, sex
- Height, weight, BMI
- Ethnicity
- Smoking history, alcohol consumption
- Medications
    - Number of different classes of antihypertensive medications being used, and whether using each class of medication
    - Total defined daily doses of antihypertensive medications being used, and defined daily doses of each class of medication
- Medical history
    - coded by MedDRA system organ class
- Physical examination
    - Presence of cardiovascular, respiratory, abdominal, neurological, genitourinary, musculoskeletal, and other abnormalities
- Clinic readings of systolic blood pressure, diastolic blood pressure, heart rate (mean of last two measurements to be used)
- ECG
    - presence of atrial fibrillation, and U-wave abnormailities

- Blood biomarkers
    - Sodium, potassium, creatinine, eGFR, urea, bicarbonate, troponin (hs-TnT), brain natriuretic peptide, renin mass, renin activity, aldosterone, aldosterone renin ratio (ARR) calculated with renin activity ($ARR_{activity}$) and with renin mass (ARR_{mass})

2.2.2. AVS RESULTS

For the FAS, whether ACTH was administered will be summarised, as will reasons for not administering ACTH. Serum ACTH will be summarised as a whole and in relation to whether ACTH was administered.

For the FAS, the following AVS measures will be summarised as a whole, and in relation to whether the results indicated bilateral adrenal hyperplasia (BAH), unilateral aldosterone-producing adenoma (APA) (left), unilateral APA (right), or were inconclusive:

- Right Adrenal Vein
    a. Cortisol (maximum value recorded per patient)
    b. Aldosterone (value corresponding to maximum cortisol)
    c. Aldosterone/Cortisol Ratio (b/a)
- Left Adrenal Vein
    a. Cortisol (maximum value recorded per patient)
    b. Aldosterone (value corresponding to maximum cortisol)
    c. Aldosterone/Cortisol Ratio (b/a)
- Lateralisation Index (ratio of higher to lower A/C ratio)
- Low IVC
    a. Cortisol (mean value recorded per patient)
    b. Aldosterone (mean value recorded per patient)
    c. Aldosterone/Cortisol Ratio (maximum value recorded per patient)
- Selectivity Index (Right) – ratio of A/C ratios between right adrenal vein and low IVC
- Selectivity Index (Left) – ratio of A/C ratios between left adrenal vein and low IVC

2.2.3. 11C-METOMIDATE PET-CT

For the FAS, whether dexamethasone was taken for 3-5 days prior to the scan will be summarised; where this has not occurred, the reasons will be summarised and/or listed.

For the FAS, the following laboratory measures will be summarised as a whole, and in relation to whether the 11C-Metomidate PET-CT results indicated BAH, unilateral APA (left), unilateral APA (right), or were inconclusive:

- Sodium, potassium, creatinine, eGFR, urea, bicarbonate, renin mass, renin activity, aldosterone, aldosterone renin ratio (ARR), serum cortisol, serum ACTH

For the FAS, the following 11C-Metomidate PET-CT measures will be summarised as a whole, and in relation to whether the results indicated BAH, unilateral APA (left), unilateral APA (right), or were inconclusive:

- Right Adrenal
    - Number of possible tumours
    - Mean tumour diameter
    - Maximum standardised uptake value (SUVmax) over all tumours, (calculated by TOF and SharpIR)
    - SUVmax over the normal adrenal (TOF and SharpIR)
    - Ratio of SUVmax over tumour to SUVmax over normal adrenal (TOF)
    - Ratio of SUVmax over tumour to SUVmax over normal adrenal (SharpIR)
    - Probability of adrenal adenoma
- Left Adrenal
    - Number of possible tumours
    - Mean tumour diameter
    - Maximum standardised uptake value (SUVmax) over all tumours, (calculated by TOF and SharpIR)
    - SUVmax over the normal adrenal (TOF and SharpIR)
    - Ratio of SUVmax over tumour to SUVmax over normal adrenal (TOF)
    - Ratio of SUVmax over tumour to SUVmax over normal adrenal (SharpIR)
    - Probability of adrenal adenoma
- SUVmax over liver (TOF and SharpIR)
- Overall Probability of Unilateral Aldesterone Producing Adenoma

For the Substudy Population, these same measures will be summarised for scans performed whilst taking spironolactone, and when off spironolactone. Bland-Altman plots and cross-tabulations will be used to assess the level of agreement between scans.

2.2.4. PARTICIPANT CHARACTERISTICS AT BASELINE

The following information collected at baseline (after study scans have been completed) will be summarised for all participants in the Full Analysis Set, and in the Substudy Population; for those in the FAS, data will be presented according to whether the participant later underwent surgical treatment:

- Medications
    - Number of different classes of antihypertensive medications being used, and whether using each class of medication
    - Total defined daily doses of antihypertensive medications being used, and defined daily doses of each class of medication
- Clinic readings of systolic blood pressure, diastolic blood pressure, heart rate
- Home readings of systolic blood pressure, diastolic blood pressure, heart rate (mean of all available readings; minimum 6 readings required)
- ECG
    - presence of atrial fibrillation, and U-wave abnormailities
- Blood biomarkers
    - Sodium, potassium, magnesium, creatinine, eGFR, urea, bicarbonate, troponin, brain natriuretic peptide, renin mass, renin activity, aldosterone, aldosterone renin ratio (ARR)
- 24-hour urine biomarkers
    - Sodium, potassium
- Spironolactone/Eplerenone therapy
- Quality of life
    - SF36
        - Physical functioning
        - Role limitations due to physical health
        - Role limitations due to emotional problems
        - Energy or fatigue
        - Emotional wellbeing
        - Social functioning
        - Pain
        - General health
        - Health change
    - Hospital Anxiety and Depression (HAD)
        - Depression
        - Anxiety
        - Total
    - Perceived Stress Score
    - Pittsburgh Sleep Quality Index
    - Fatigue Symptom Inventory
        - Level of Fatigue on Most Fatigued Day
        - Level of Fatigue on Least Fatigued Day
        - Level of Fatigue on Average
        - Level of Fatigue Now

- Days Fatigued in Week
- How Much of Day Spent Fatigued
- Pattern of Fatigue
- Disruption Index

2.2.5. Week 2

The following information collected at 2 weeks will be summarised for all participants in the Full Analysis Set, as a whole and according to whether the participant later underwent surgical treatment:

- Medications
  - Number of different classes of antihypertensive medications being used, and whether using each class of medication
  - Total defined daily doses of antihypertensive medications being used, and defined daily doses of each class of medication
- Clinic readings of systolic blood pressure, diastolic blood pressure, heart rate
- Home readings of systolic blood pressure, diastolic blood pressure, heart rate
- Blood biomarkers
  - Sodium, potassium, creatinine, eGFR, urea, bicarbonate
- Spironolactone/Eplerenone therapy

2.2.6. Week 4

The following information collected at 4 weeks will be summarised for all participants in the Full Analysis Set, as a whole and according to whether the participant later underwent surgical treatment:

- Medications
  - Number of different classes of antihypertensive medications being used, and whether using each class of medication
  - Total defined daily doses of antihypertensive medications being used, and defined daily doses of each class of medication
- Clinic readings of systolic blood pressure, diastolic blood pressure, heart rate
- Home readings of systolic blood pressure, diastolic blood pressure, heart rate
- Blood biomarkers
  - Sodium, potassium, creatinine, eGFR, urea, bicarbonate

2.2.7. MDT Decision

MDT decisions (proceed to surgery, or medical management) will be summarised in relation to the diagnosis (BAH, APA, or inconclusive) from each test modality, and combinations of test results (see Section 1.5).

2.2.8. SURGERY

The following information, regarding surgery, will be summarised for those participants who undergo surgery:

- Location of surgery (left/right)
- Method of surgery (open/laparoscopic)
- Number of adenomas/nodules
- Whether immunohistochemistry was performed
  - if yes, results for CYP11B1, CYP11B2, KCNJ5, and NPNT (negative/+/++/+++)
- Presence of hyperplasia in the normal (adjacent) adrenal

For all adenomas/nodules, the following data will be summarised:

- Size (mm)
- Cell type
- CYP11B1, CYP11B2, KCNJ5, and NPNT (negative/+/++/+++)
- Somatic mutation (KCNJ5/ATP1A/ATP2B3/CTNNB1/CACNAID/CDM1/Not Performed/Other)

2.2.9. 4 WEEKS POST-SURGERY

The following information, collected 4 weeks after surgery, will be summarised for those participants who undergo surgery:

- Medications
  - Number of different classes of antihypertensive medications being used, and whether using each class of medication
  - Total defined daily doses of antihypertensive medications being used, and defined daily doses of each class of medication
- Clinic readings of systolic blood pressure, diastolic blood pressure, heart rate
- Home readings of systolic blood pressure, diastolic blood pressure, heart rate
- Blood biomarkers
  - Sodium, potassium, creatinine, eGFR, urea, bicarbonate, renin mass, renin activity, aldosterone, aldosterone renin ratio (ARR)

2.2.10. 3 MONTHS POST-SURGERY

The following information, collected 3 months after surgery, will be summarised for those participants who undergo surgery:

- Medications
  - Number of different classes of antihypertensive medications being used, and whether using each class of medication
  - Total defined daily doses of antihypertensive medications being used, and defined daily doses of each class of medication

- Clinic readings of systolic blood pressure, diastolic blood pressure, heart rate
- Home readings of systolic blood pressure, diastolic blood pressure, heart rate
- Blood biomarkers
    - Sodium, potassium, creatinine, eGFR, urea, bicarbonate, renin mass, renin activity, aldosterone, aldosterone renin ratio (ARR)

2.2.11. 6 Months Post-Surgery / 9-12 Months Post-MDT Decision

The following information, collected 6 months after surgery, or 9-12 months after the MDT decision, will be summarised for those all participants and in relation to whether they underwent surgery:

- Medications
    - Number of different classes of antihypertensive medications being used, and whether using each class of medication
    - Total defined daily doses of antihypertensive medications being used, and defined daily doses of each class of medication
- Clinic readings of systolic blood pressure, diastolic blood pressure, heart rate
- Home readings of systolic blood pressure, diastolic blood pressure, heart rate
- ECG
    - presence of atrial fibrillation, and U-wave abnormailities
- Blood biomarkers
    - Sodium, potassium, magnesium, creatinine, eGFR, urea, bicarbonate, troponin, brain natriuretic peptide, renin mass, renin activity, aldosterone, aldosterone renin ratio (ARR)
- Cardiac Magnetic Resonance imaging
    - whether performed
- Quality of Life
    - SF36
        - Physical functioning
        - Role limitations due to physical health
        - Role limitations due to emotional problems
        - Energy or fatigue
        - Emotional wellbeing
        - Social functioning
        - Pain
        - General health
        - Health change
    - Hospital Anxiety and Depression (HAD)
        - Depression
        - Anxiety
        - Total
    - Perceived Stress Score

- Pittsburgh Sleep Quality Index
- Fatigue Symptom Inventory
  - Level of Fatigue on Most Fatigued Day
  - Level of Fatigue on Least Fatigued Day
  - Level of Fatigue on Average
  - Level of Fatigue Now
  - Days Fatigued in Week
  - How Much of Day Spent Fatigued
  - Pattern of Fatigue
  - Disruption Index

2.2.12. 12 Months Post-Surgery

The following information, collected 12 months after surgery, will be summarised for those participants who undergo surgery:

- Medications
  - Number of different classes of antihypertensive medications being used, and whether using each class of medication
  - Total defined daily doses of antihypertensive medications being used, and defined daily doses of each class of medication
- Clinic readings of systolic blood pressure, diastolic blood pressure, heart rate
- Home readings of systolic blood pressure, diastolic blood pressure, heart rate
- Blood biomarkers
  - Sodium, potassium, creatinine, eGFR, urea, bicarbonate, renin mass, renin activity, aldosterone, aldosterone renin ratio (ARR)

2.2.13. 24 Months Post-Surgery / Post-MDT Decision

The following information, collected 24 months after surgery / MDT decision, will be summarised for those all participants and in relation to whether they underwent surgery:

- Medications
  - Number of different classes of antihypertensive medications being used, and whether using each class of medication
  - Total defined daily doses of antihypertensive medications being used, and defined daily doses of each class of medication
- Clinic readings of systolic blood pressure, diastolic blood pressure, heart rate
- Home readings of systolic blood pressure, diastolic blood pressure, heart rate
- Blood biomarkers
  - Sodium, potassium, creatinine, eGFR, urea, bicarbonate, renin mass, renin activity, aldosterone, aldosterone renin ratio (ARR)

2.3. Efficacy Outcomes

2.3.1. Primary Outcomes

The primary outcomes will be analysed hierarchically. If the first primary analysis is statistically significant at p<0.05, then the second primary outcome will be analysed; if the second primary analysis is statistically significant at p<0.05, then the second primary outcome will be analysed; and so on. If any primary analysis fails to reach p<0.05, then subsequent analyses will be considered secondary analyses.

Each primary outcome will be analysed in the subgroup of FAS patients who undergo surgery.

- Cure of hypertension at 6 months (non-inferiority analyses)
  - comparing accuracy of AVS and 11C-Metomidate PET-CT
  - each scan judged to be accurate if it indicated unilateral disease and cure of hypertension was achieved after surgery, or if the scan did not indicate unilateral disease and cure was not achieved after surgery
  - report estimated difference in accuracy (Newcombe-Wilson method for paired data) between 11C-Metomidate PET-CT and AVS, with 95% CI
  - non inferiority of 11C-Metomidate PET-CT will be declared if the lower limit of the above 95% CI is greater than -17%
  - each measure of cure (see Section 1.8) will be analysed in turn

- Cure of hypertension at 6 months (superiority analyses)
  - in addition to above, report p-value from exact McNemar test
  - superiority of 11C-Metomidate PET-CT will be declared if p<0.05, and the lower limit of the 95% CI is greater than 0%

2.3.2. Secondary Outcomes

Secondary (and Primary) outcomes will be compared between participants who underwent surgery and those who did not, and for those who underwent surgery, between those for whom surgery was indicated by AVS only, 11C-Metomidate PET-CT only, or both tests.

Linear, binary logistic, or ordinal logistic regression models will be used, as appropriate. Models will be adjusted for age and sex, and the baseline measure of the outcome (where appropriate). To assess differences between those who underwent surgery and those who did not, a categorical variable for whether surgery was performed will be included. To assess differences between AVS and 11C-Metomidate PET-CT, a 3-level categorical variable for whether surgery

was guided by AVS only, 11C-Metomidate PET-CT only, or both tests, will be included.

Similar models will be used to investigate other predictors of outcome after surgery, in the subset of participants who underwent surgery. In line with the secondary objectives, there is particular interest in the following predictors:

- BP response to therapy with spironolactone/eplerenone, measured as:
    - achievement of home SBP <135 mmHg (or clinic SBP <140 mmHg) at week 4
    - achievement of home SBP <135 mmHg (or clinic SBP <140 mmHg) at week 4, without an increase in other BP medication (as per PASO criteria)
    - change in SBP (home SBP if available, or clinic SBP) between baseline and week 4
    - change in SBP (home SBP if available, or clinic SBP) between baseline and week 4, correcting for changes in other BP medication
- Age, sex, ethnicity
- Baseline BP
- Number of classes of antihypertensive medication at baseline
- Tumour genotype, individually and grouping KCNJ5 mutations vs all other mutations
- Tumour immunohistochemical classification
- AVS parameters
    - Contralateral suppression (aldosterone/cortisol in the contralateral adrenal vein < 50% of aldosterone/cortisol ratio in IVC)
    - Contralateral suppression (aldosterone/cortisol in the contralateral adrenal vein < 100% of aldosterone/cortisol ratio in IVC)
- PET parameters
    - number of nodules on each side
    - 'Probability of adrenal adenoma' on contralateral side'
    - SUVmax (TOF) for [hottest] tumour/normal on side of tumour
    - SUVmax (TOF) for [hottest] tumour/liver
    - SUVmax ratio (tumour /contralateral adrenal) using SharpIR;

- 24h-urine steroid profile

Univariate, and multivariable models will be fitted to explore these associations.

2.3.3. EXPLORATORY OUTCOMES

Not applicable.

2.3.4. Additional Analyses

It is likely that additional analyses will be carried out after the analyses specified in this SAP have been completed.

We shall investigate the use of Receiver Operator Characteristic (ROC) curve analyses, under the assumption that for subjects where neither test indicated surgery, both tests had accurately ruled out unilateral disease. We shall investigate alternative thresholds for continuous measures from AVS and 11C-Metomidate PET-CT. These analyses will be performed for all participants, and within subgroups defined by whether the alternative test was positive, negative, or ambiguous.

2.3.5. Sub-group Analyses

The study is not powered to detect subgroup effects. Any subgroup analyses that are performed after the analyses specified in this SAP have been completed will be exploratory only.

2.3.6. Sensitivity Analyses

The primary analyses will be repeated after the exclusion of subjects for whom AVS was a technical failure.

2.4. Safety Outcomes

2.4.1. Serious Adverse Events

The characteristics of all serious adverse events (SAEs) recorded in the study will be summarised, including severity, relationship to study procedures, outcome, and duration. The number and percentage of participants experiencing at least one SAE will be reported as a whole and by MedDRA system organ class and preferred term. Summaries will be provided as a whole and in relation to whether the participant underwent surgery. For those who underwent surgery, summaries will be provided according to whether the onset date was prior to or after the date of surgery.

3. Data Conventions

Any data rules (e.g. handling of partial dates, missing data imputation) will be documented as part of the Statistical Analysis Validation Plan (SAVP) for the project, in a separate analysis dataset specification document.

4. Document History

This is version 1 of the SAP for MATCH, dated 22/02/2021, the initial creation.

5. TABLES

A full list of data tables will be developed and agreed prior to the final analysis.

6. FIGURES

No figures will be produced as part of the statistical outputs for this study, but may be generated in support of future manuscript preparation.

7. LISTINGS

No formal data listings will be produced. The analysis datasets will be made available to the Chief Investigator after the final analysis.
